# Deep transcriptome profiling reveals limited conservation of A-to-I RNA editing in *Xenopus*

**DOI:** 10.1186/s12915-023-01756-2

**Published:** 2023-11-09

**Authors:** Tram Anh Nguyen, Jia Wei Joel Heng, Yan Ting Ng, Rui Sun, Shira Fisher, Gokce Oguz, Pornchai Kaewsapsak, Shifeng Xue, Bruno Reversade, Adaikalavan Ramasamy, Eli Eisenberg, Meng How Tan

**Affiliations:** 1 School of Chemistry, Chemical Engineering and Biotechnology, Nanyang Technological University, Singapore, Singapore; 2 Genome Institute of Singapore, Agency for Science Technology and Research, Singapore, Singapore; 3 School of Biological Sciences, Nanyang Technological University, Singapore, Singapore; 4 Faculty of Life Sciences, The Mina and Everard Goodman, Bar-Ilan University, Ramat Gan, Israel; 5 Department of Biochemistry, Faculty of Medicine, Chulalongkorn University, Bangkok, Thailand; 6 Institute of Molecular and Cell Biology, Agency for Science Technology and Research, Singapore, Singapore; 7 Department of Biological Sciences, National University of Singapore, Singapore, Singapore; 8 Yong Loo Lin School of Medicine, National University of Singapore, Singapore, Singapore; 9 Department of Medical Genetics, School of Medicine (KUSoM), Koç University, Istanbul, Turkey; 10 Raymond and Beverly Sackler School of Physics and Astronomy, Tel Aviv University, Tel Aviv, Israel; 11 HP-NTU Digital Manufacturing Corporate Lab, Nanyang Technological University, Singapore, Singapore

**Keywords:** RNA editing, ADAR, *Xenopus*

## Abstract

**Background:**

*Xenopus* has served as a valuable model system for biomedical research over the past decades. Notably, ADAR was first detected in frog oocytes and embryos as an activity that unwinds RNA duplexes. However, the scope of A-to-I RNA editing by the ADAR enzymes in *Xenopus* remains underexplored.

**Results:**

Here, we identify millions of editing events in *Xenopus* with high accuracy and systematically map the editome across developmental stages, adult organs, and species. We report diverse spatiotemporal patterns of editing with deamination activity highest in early embryogenesis before zygotic genome activation and in the ovary. Strikingly, editing events are poorly conserved across different *Xenopus* species. Even sites that are detected in both *X. laevis* and *X. tropicalis* show largely divergent editing levels or developmental profiles. In protein-coding regions, only a small subset of sites that are found mostly in the brain are well conserved between frogs and mammals.

**Conclusions:**

Collectively, our work provides fresh insights into ADAR activity in vertebrates and suggest that species-specific editing may play a role in each animal’s unique physiology or environmental adaptation.

**Supplementary Information:**

The online version contains supplementary material available at 10.1186/s12915-023-01756-2.

## Background

*Xenopus* is one of the key model organisms used in developmental and cell biology studies [1]. It offers several advantages as an experimental system. First, large abundant eggs are readily available from a single mating pair, and the embryos are easily injected, can withstand extensive surgical manipulation, and provide a rich source of material for biochemical studies. Second, embryogenesis is rapid, robust, and occurs outside the body, allowing for easy access to all developmental stages. Third, many cellular pathways are conserved between *Xenopus* and mammals, which enables the use of the frog to model human disease. Over the past decades, research on *Xenopus* has led to breakthroughs in our understanding of nuclear reprogramming [2], embryonic induction and patterning [3, 4], regeneration [5], electrophysiology [6, 7], and the cell cycle [8–10]. Furthermore, the genomes of *Xenopus laevis* and *Xenopus tropicalis*, two frog species which are most commonly used by researchers, have been sequenced [11, 12] and their gene expression profiles have also been studied by DNA microarrays [13] and Illumina RNA sequencing (RNA-seq) [12, 14, 15].

Adenosine-to-inosine (A-to-I) RNA editing is a widespread RNA modification in higher eukaryotes and is catalyzed by the adenosine deaminase acting on RNA (ADAR) family of enzymes [16]. ADARs were initially reported as enzymes that unwound double-stranded RNAs (dsRNAs) in the oocytes and embryos of *X. laevis* before researchers realized that a base had been chemically modified [17–20]. Three members of this family are encoded in the vertebrate genome, namely ADAR1 (also known simply as ADAR), ADAR2 (also known as ADARB1), and ADAR3 (also known as ADARB2). Only ADAR1 and ADAR2 are catalytically active, as several mutations in the deaminase domain of ADAR3 render it inactive [21]. Inosine preferentially base pairs with cytidine and is recognized by cellular machineries primarily as guanosine (G). Hence, A-to-I editing effectively results in an A-to-G nucleotide change and can give rise to different outcomes, including the generation of new protein isoforms [22–24], alteration of splicing patterns [25–28], and modulation of microRNA (miRNA) targeting [29, 30].

Various algorithmic approaches have been developed to uncover RNA editing sites from high throughput sequencing data in both vertebrates and invertebrates. Expectedly, most efforts have been focused on human, with millions of A-to-I editing sites identified to date [31–36]. Nevertheless, the RNA editome of some other animals has also been systematically examined, including mouse [37–40], cephalopods [41–43], zebrafish [44, 45], *Drosophila* [36, 46–50], and *Caenorhabditis elegans* [51–54]. Diverse patterns of RNA editing are frequently observed [44, 55], suggesting intricate regulation of ADAR activity. Furthermore, a common theme that emerges is that most editing occurs in non-coding regions of the transcriptome, even in cephalopods where recoding events are more prevalent than other organisms. Repetitive elements are particularly susceptible to editing because they often exist as inverted pairs and form ideal dsRNA substrates for the ADAR enzymes. Notably, in long dsRNA targets, the deaminases are known to install clusters of edits, which can be missed by conventional bioinformatic approaches that allow only a small number of mismatches between the sequencing reads and the reference genome. Consequently, a hyper-editing analysis pipeline was developed to identify extensively edited regions [56]. Application of this pipeline on mostly brain samples from 21 eukaryotes uncovered abundant clusters of editing sites that correlated with the extent of dsRNA formation in each species [57].

Despite the major role that *Xenopus* plays in biomedical research, the RNA editing landscape in frogs is yet unknown. Our previous hyper-editing analysis on a single *Xenopus* brain dataset revealed a large number of clustered A-to-G mismatches that was second only to octopus, in large part due to the Harbinger repeat family, which has a palindromic consensus sequence and thus can fold to create a stable dsRNA structure [57]. Here, we expand on our earlier work by comprehensively mapping the A-to-I editing landscape in *Xenopus* across development, multiple adult organs, and species. Through several computational pipelines, we identify hundreds of thousands of editing sites in *X. laevis* and *X. tropicalis* using datasets from six different studies. We observe high ADAR activity during early embryogenesis and in the ovary and provide evidence that many edited transcripts in the 1-cell zygote might be maternally deposited. Unexpectedly, most editing events are poorly conserved within the *Xenopus* genus, even in protein-coding sequences. Many conserved coding sites are more extensively edited in one species than the other and the same genes can be targeted at distinct sets of positions within their protein-coding region in different organisms. Collectively, our work provides not only a useful resource for the scientific community but also novel insights into species-specific adaptation of A-to-I RNA editing.

## Results

### Discovery of editing sites in *X*. *laevis*

**Fig. 1:**
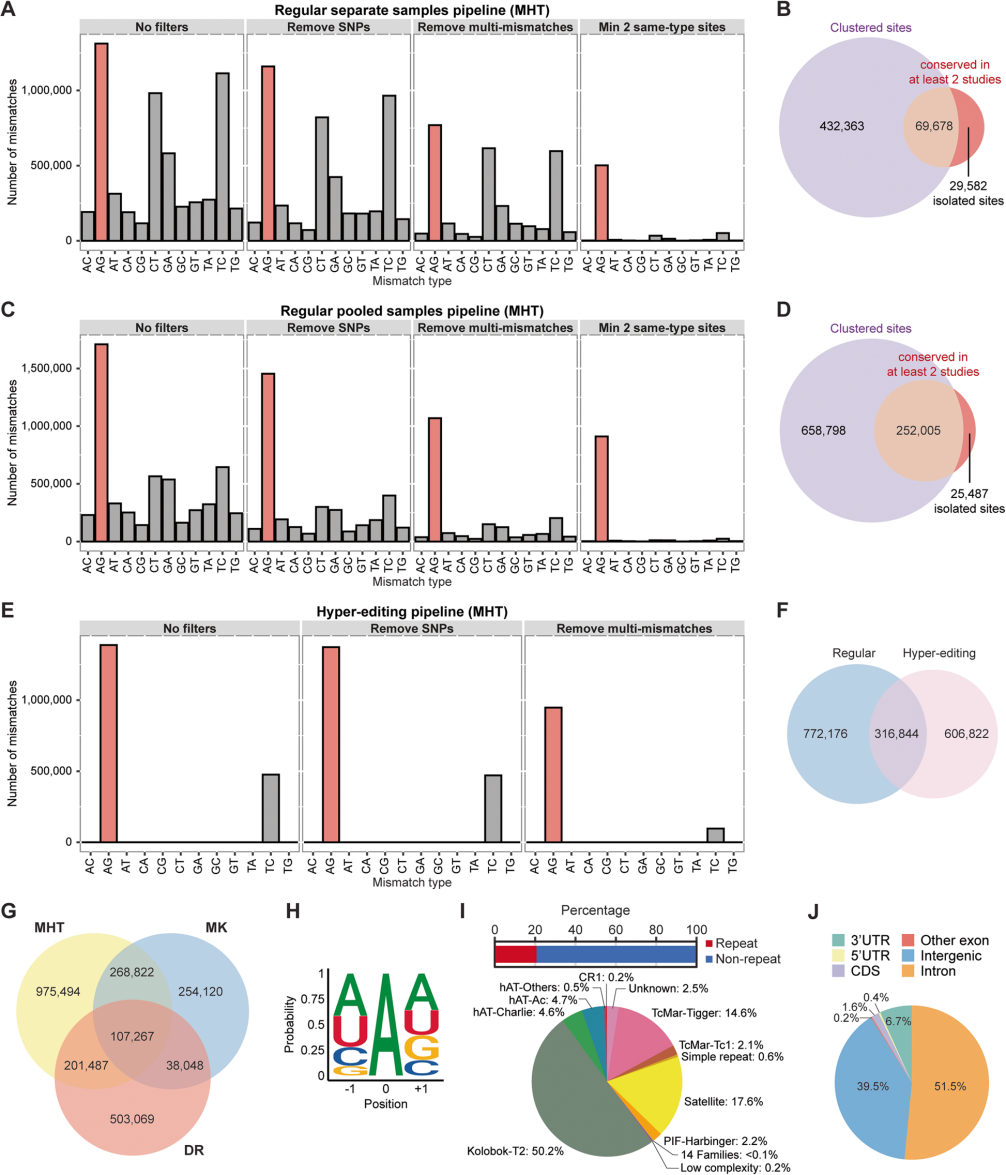
Identification of A-to-I editing sites with various analysis pipelines. **A** Distribution of mismatch types at different steps of the separate samples analysis workflow. Each two-letter combination, XY, indicates X-to-Y mismatch. Altogether, there are 12 possible mismatch types. The first histogram shows the raw output from REDItools, while the second histogram shows the distribution after elimination of genomic SNPs. “Remove multi-mismatches” means that variants with other types of mismatches in their vicinity are discarded. The last filter, labeled as “min 2 same-type sites”, selects for clustered editing events, which are characteristic of ADAR targets. MHT refers to the datasets generated in this study. A total of 36 samples were analyzed. **B** Venn diagram indicating the number of isolated editing sites recovered in the separate samples analysis workflow due to their detection in at least two different studies. **C** Distribution of mismatch types at different steps of the pooled samples analysis workflow. **D** Venn diagram indicating the number of isolated editing sites recovered in the pooled samples analysis workflow due to their detection in at least two different studies. **E** Distribution of mismatch types at different steps of the hyper-editing analysis workflow. The last filter, “min 2 same-type sites”, is unnecessary here as hyper-edited loci often occur in clusters. **F** Venn diagram showing the number of editing events detected using regular read alignment and REDItools or the hyper-editing pipeline where mapping was done with all As converted to Gs. **G** Venn diagram showing the number of A-to-I editing sites found in each of the three studies, which are indicated by the initials of the corresponding authors. DR refers to the datasets reported by Daniel Rokhsar [12], while MK refers to the datasets reported by Marc Kirschner [15]. **H** ADAR motif in *X. laevis* based on our curated list of editing sites. **I** Editing in repetitive regions of the *X. laevis* genome. Unlike human and mouse, minority of the frog ADAR targets were found in repeats. The pie chart shows the distribution of A-to-I editing sites in various repeat families. Fourteen annotated repeat families contained comparatively few editing events and thus were grouped together in a single slice of the pie chart. **J** Genomic locations of editing sites in *X. laevis*. Most sites resided in non-coding regions of the genome, such as introns and 3’UTRs

To build a frog RNA editing atlas, we generated stranded Illumina RNA-seq libraries for *X. laevis* embryos at various developmental stages. After mapping the sequencing reads to the reference genome (xenLae2), we searched for editing sites in our samples individually using REDItools [58], imposing a minimum total coverage of 10 × , a minimum variant coverage of 3 × , and a minimum editing rate of 1% (Fig. 1A). The number of A-to-G mismatches was the highest among all possible DNA-RNA mismatches, but other transitions (T-to-C, C-to-T, and G-to-A) were also detected at excessive levels, indicating a high false discovery rate (FDR). To eliminate single nucleotide polymorphisms (SNPs) and DNA mutations that might confound our RNA editing analysis, we called variants in publicly available whole genome sequencing (WGS) data for *X. laevis* [12] and sequenced and analyzed the genomic DNA from each of our clutches. This step marginally improved the A-to-G percentage from 22.7% to 25.1%. Subsequently, to further reduce the false detection rate, we filtered for sites that were not flanked by mismatches of other types within a ± 20 nucleotide (nt) window, since such clusters of sites with multiple substitution types are indicative of mapping errors. Additionally, since A-to-I editing events often occur in clusters, we discarded isolated sites with no other mismatch of the same type within a ± 20 nt window. Both filters helped improve the A-to-G percentage to 80.3%. The FDR was estimated to be 2.7% (the G-to-A percentage divided by the A-to-G percentage multiplied by 100).

In addition, we examined Illumina RNA-seq data from another two studies, which included embryos and adult tissues [12, 15]. Every sample was analyzed individually by REDItools with the same set of filters implemented (Additional file 1: Fig. S1). Overall, each study yielded over a hundred thousand A-to-I editing sites. However, due to the last filter, only clustered sites had been identified so far. To recover isolated sites, we removed the last filter and looked for sites present in at least two different studies, since bona fide editing events are more likely to reoccur than genomic polymorphisms. We further required a more stringent minimum variant coverage of 5 × in each study. From this approach, tens of thousands of isolated sites were uncovered, 76.4% of which were A-to-G mismatches (Fig. 1B and Additional file 1: Fig. S1-S2). The FDR was estimated to be 5.8%.

Some authentic RNA editing sites might have avoided detection due to low sequencing coverage. To achieve higher sensitivity for such sites, we pooled the reads from all samples within the same study and analyzed the combined dataset in the same way as before with REDItools. More editing sites could be discovered in this manner with a larger percentage of them being the A-to-G type (Fig. 1C and Additional file 1: Fig. S3). For our own dataset, using the pooled samples approach, 92.4% of the variants were A-to-G mismatches with an estimated FDR of 1.1%. We also identified isolated sites by requiring them to be present in at least two studies and be covered by at least 5 variant reads in each study (Fig. 1D and Additional file 1: Fig. S3-S4). Here, the A-to-G percentage was 85.1% and the FDR was estimated to be 3.3%. The extra step of recovering isolated sites is necessary to alleviate the issue of bona fide coding sites being removed by the last filter because they often do not occur in clusters (Additional file 1: Fig. S5). Overall, compared to the separate samples approach, the pooled samples approach gave a higher detection accuracy.

ADAR enzymes often target multiple adenosines within the same dsRNA substrate, resulting in numerous mismatches between sequencing reads and the reference genome that would prevent the reads from being mapped. To search for such hyper-editing sites, we implemented a previously reported pipeline that transformed all As to Gs in unmapped reads and the reference genome before realignment [56]. The original sequences were then retrieved to identify dense clusters of mismatches. With this hyper-editing pipeline, we discovered hundreds of thousands of editing events, most of which were not detected by the regular approach using REDItools (Fig. 1E-F and Additional file 1: Fig. S6).

In total, we identified 2,348,307 distinct A-to-I RNA editing sites in *X. laevis* from the three studies (Fig. 1G). Notably, 98.3% of these sites were homozygous reference in WGS data with at least 10 supporting “A” reads, suggesting that we successfully filtered out genomic polymorphisms. Examination of the nucleotide identity flanking all editing sites revealed the familiar suppression of G one base upstream, which is common of the ADAR motif in every metazoan studied so far (Fig. 1H). Interestingly, we did not observe an overrepresentation of G one base downstream, which is the case in human, mouse, and zebrafish [31–36, 39, 44, 59]. Instead, we found an enhancement of A and a depletion of C, which has previously been reported in several other animals as well [57].

Next, we examined the genomic locations of the editing sites. In human and mouse, most editing events occur within repeat elements like Alu and B1/B2 SINEs (short interspersed nuclear elements), respectively. However, in *X. laevis*, we observed that most editing sites did not fall in annotated repeat regions (Fig. 1I). Among the repeat sites, a sizeable percentage (50.2%) occurred in Kolobok-T2 elements, which could fold into stable dsRNA structures (Additional file 1: Fig. S7). Furthermore, a substantial percentage (39.5%) of the editing sites resided in intergenic regions (Fig. 1J). For the sites in genic regions, vast majority of them occurred in introns and 3’ untranslated region (UTR), and most edited genes contained more than one editing site (Additional file 1: Fig. S8), consistent with the notion that ADARs can target multiple adenosines within a dsRNA structure. Collectively, the large number of ADAR target sites in *X. laevis* suggests that RNA editing is likely to serve as a key gene regulatory mechanism in frogs.

### A-to-I editing during development of *X*. *laevis*

**Fig. 2:**
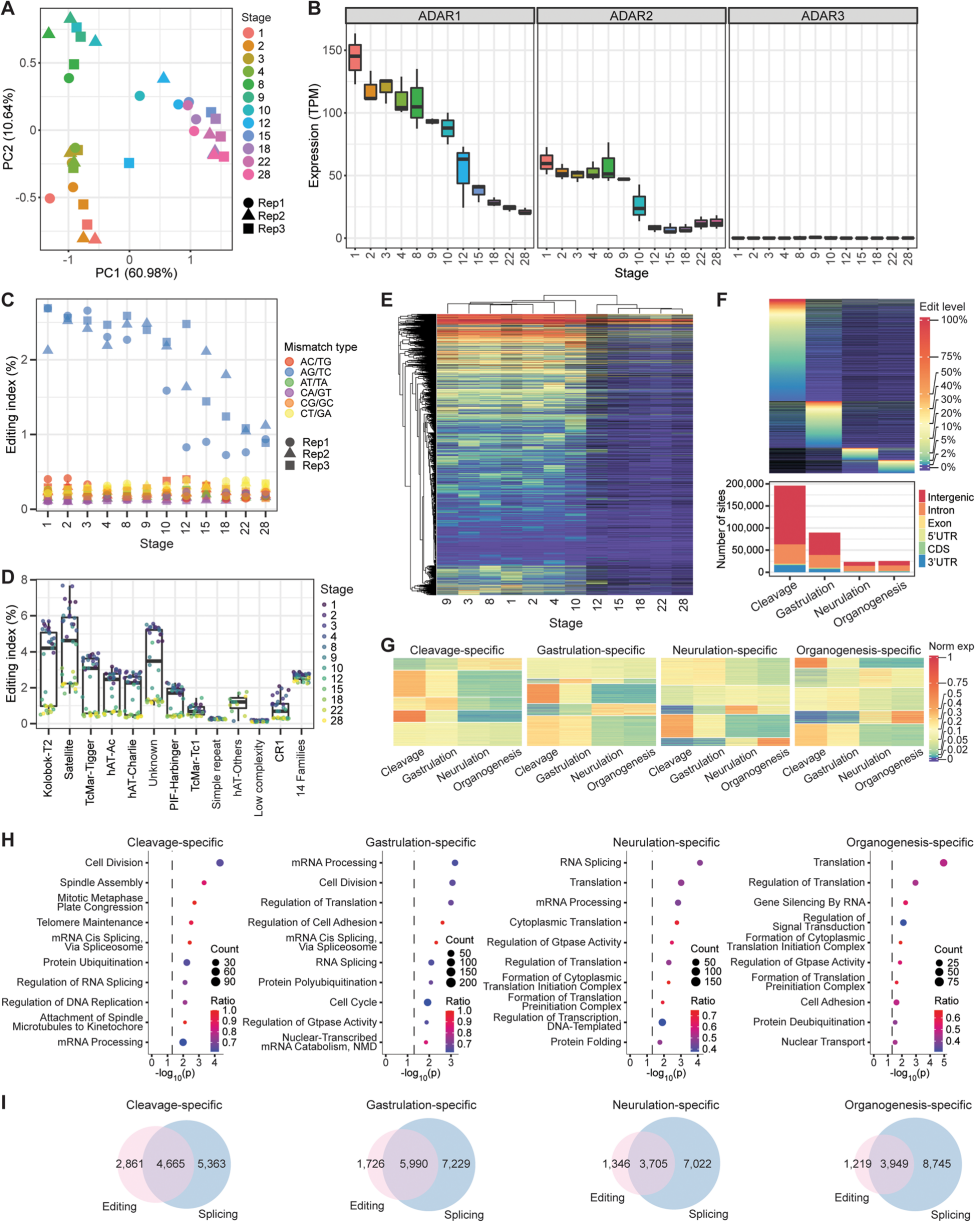
A-to-I editing landscape across development of *X. laevis*. **A** PCA plot based on gene expression values showing segregation of our embryonic samples according to developmental stages. **B** Transcript levels of ADAR enzymes across development as quantified from our RNA-seq data. The ADAR expression values are provided in Additional File 2. **C** Global editing index measured across all repeat families in our study. **D** Editing index for each individual repeat family in our study. While ADAR activity was variable across the repeat families, it was consistently higher during the beginning stages of development regardless of repeat type. Fourteen annotated repeat families contained comparatively few editing events and thus were grouped together for calculation of the index. **E** Hierarchical clustering of editing levels. Each row is a different editing site, while each column is a different developmental stage interrogated in our study. **F** Many transcriptomic loci were targeted by ADARs in only a single developmental process. Top: Heatmap depicting the editing rates of these process-specific sites. Bottom: Genomic locations of the process-specific sites. **G** Expression heatmaps of genes containing process-specific editing sites. **H** Top 10 GO terms associated with each set of process-specific editing sites. Dotted line indicates the *p*-value threshold of 0.05. NMD refers to nonsense-mediated decay. **I** Venn diagrams showing the numbers of edited genes and alternatively spliced genes for each developmental process. All the overlaps between editing and splicing were greater than expected (*P* < 2.2e-16, hypergeometric test), with representation factors ranging from 3.9 to 4.6

To gain deeper insights into the RNA editome, we sought to study the dynamics of editing over the development of *X. laevis*. Based on principal component analysis (PCA) of expression levels, our embryonic samples segregated clearly by developmental timepoints (Fig. 2A), indicating proper staging of the samples. Interestingly, ADAR1 and ADAR2 were highly expressed in early embryos and their expression levels only declined in later stages after zygotic genome activation (Fig. 2B), a pattern that was also observed in zebrafish [44]. To quantify the global deaminase activity in each sample, we calculated the editing index over all repeats, which was modeled after the human Alu editing index (AEI) [60], and observed that the index mirrored the trend of ADAR1 and ADAR2 expression over the course of development (Fig. 2C). We further quantified the editing index for each specific repeat family and found that it varied substantially across repeat families, with satellite repeats exhibiting an overall highest level of editing (Fig. 2D). Nevertheless, within each family, editing activity was again highest in early embryos and declined to much lower levels in later stages of development. Subsequently, we examined individual editing sites at every stage. Notably, while the number of detected sites was positively correlated with sequencing depth as expected, we also observed that samples for earlier stages gave a higher slope than samples for later stages (Additional file 1: Fig. S9A). Hence, more sites could be detected per million mapped reads in earlier developmental stages than the later stages (Additional file 1: Fig. S9B). Moreover, although the number of sites declared as being edited would naturally depend on the editing level cutoff, the earlier stages always yielded more sites than the later stages regardless of this threshold (Additional file 1: Fig. S9C). Additionally, our curated sites were deaminated at substantially higher levels in earlier stages than in the later stages during and after gastrulation (Additional file 1: Fig. S9D). Consistently, clustering analysis showed that the early developmental stages grouped together with a more active editing profile than the later stages (Fig. 2E). Similar results were obtained with datasets from the other two studies (MK and DR) (Additional file 1: Fig. S10-S13), confirming that editing activity is highest early in development before tapering off as embryogenesis progresses, which aligns with the shifts in the expression of ADAR1 and ADAR2.

We wondered about the functions of A-to-I editing in the major developmental processes, namely cleavage (stages 0–8), gastrulation (stages 8.5–12), neurulation (stages 13–21), and organogenesis (stages 22–40). To this end, we first combined datasets from the three studies (MHT, MK, and DR) since expression and editing level measurements were reproducible across the studies (*r* > 0.7) (Additional file 1: Fig. S14-S15). We then obtained process-specific editing sites by filtering for positions that were covered in two or more processes by at least 10 reads each, were edited at a minimum level of 1%, and exhibited at least threefold higher editing rate in one process over the other developmental phases. To be more confident of our context-specific sites, we chose a threefold cutoff to ensure that the mean editing levels in the other non-targeted processes were sufficiently low (Additional file 1: Fig. S16). Consistently, the number of process-specific editing events was highest during cleavage followed by gastrulation (Fig. 2F). Notably, intergenic sites accounted for a sizeable proportion of such editing events, indicating the presence of many unannotated transcripts in the *X. laevis* genome. We next asked if editing correlated with gene expression. Clustering analysis of the expression of genes edited in a process-specific manner revealed a complex relationship (Fig. 2G). For example, while the editing of some sites correlated positively with gene expression, the editing of other sites exhibited an inverse relationship with expression, suggesting that A-to-I editing can both enhance and reduce transcript levels in a context-specific manner (Additional file 1: Fig. S17-S18). Subsequently, we performed gene ontology (GO) analysis of the process-specific sites to gain deeper functional insights. Interestingly, translation was among the top two GO terms in neurulation and organogenesis, while cell division featured more prominently in cleavage and gastrulation (Fig. 2H). Additionally, terms related to RNA processing emerged repeatedly in the analysis. Moreover, since A-to-I editing had previously been shown to regulate RNA splicing [25–28], we detected alternative splicing events using MAJIQ [61] and intersected the process-specific edited genes with the alternatively spliced genes. Most genes that were deaminated in a process-specific manner also contained splicing variations in the same transcript (Fig. 2I). Importantly, all the overlaps in gene sets were statistically significant (*P* < 2.2e-16, hypergeometric test). Taken together, our results suggest that editing may perform multiple functions over the course of vertebrate development.

### Editing landscape in adult tissues of *X*. *laevis*

**Fig. 3:**
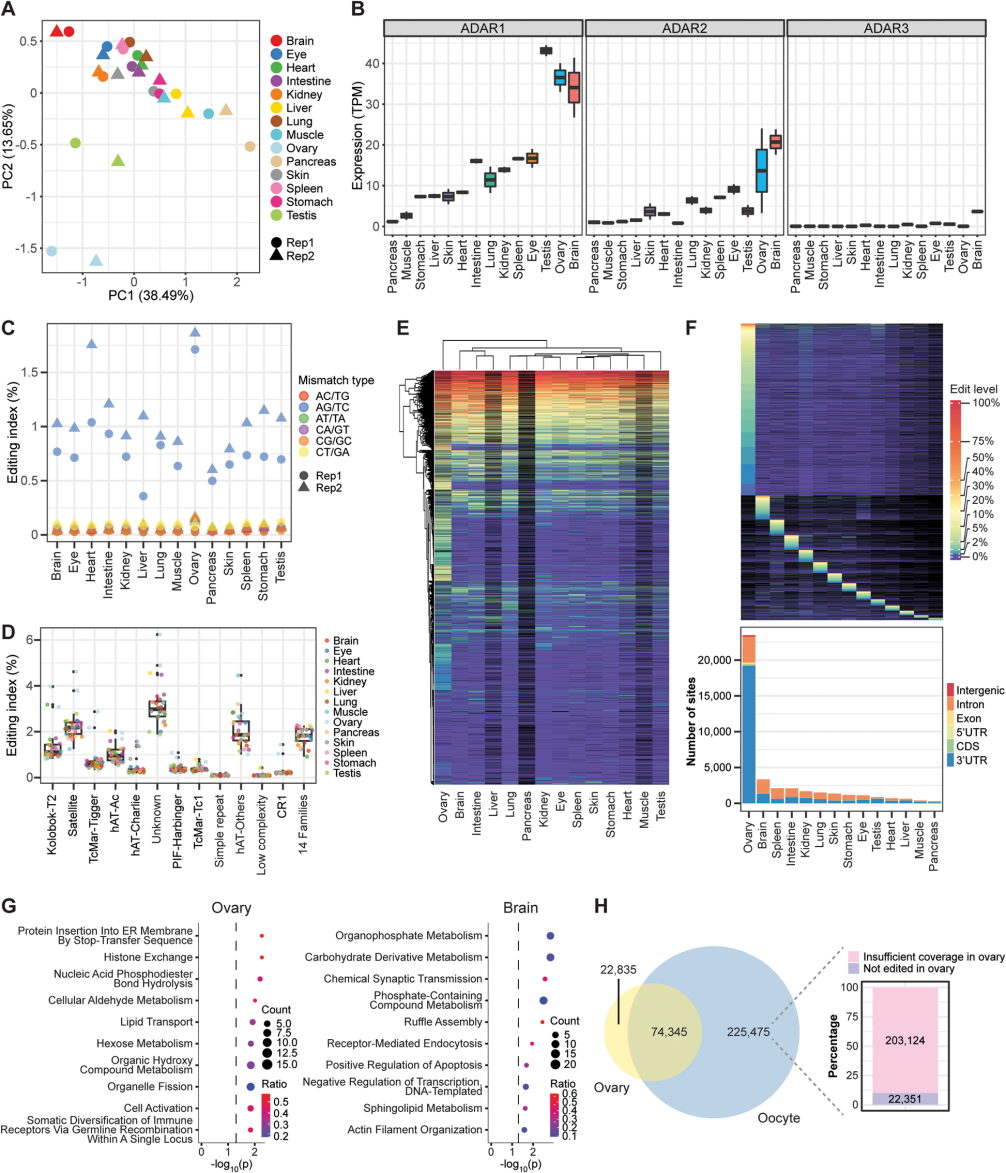
A-to-I editing landscape in *X. laevis* tissues. **A** PCA plot based on gene expression values showing clear separation of gonad samples. **B** Transcript levels of ADAR enzymes in various adult tissues. The ADAR expression values are provided in Additional File 2. **C** Global editing index measured across all repeat families in various adult tissues. **D** Editing index for each individual repeat family in various adult tissues. Fourteen repeat families contained relatively few editing events and thus were grouped together for calculation of the index. **E** Hierarchical clustering of editing levels. Each row is a different editing site, while each column is a different adult tissue. **F** Many loci were targeted by ADARs in only a single adult tissue. Top: Heatmap depicting the editing rates of these tissue-specific sites. Bottom: Genomic locations of the tissue-specific sites. **G** Top 10 GO terms associated with ovary-specific and brain-specific editing sites. Dotted line indicates the *p*-value threshold of 0.05. ER refers to endoplasmic reticulum. **H** Comparison of editing events identified in oocytes with those identified in the ovary

The RNA editome has been extensively studied by us and others in adult tissues of different vertebrates like human [55, 62], mouse [37–39, 55], and zebrafish [44], but not yet the frog. To address this gap, we examined RNA-seq data generated from 14 adult tissues of *X. laevis* [12]. PCA based on gene expression profiles revealed that the gonads, namely ovary and testis, were most distinct from the other organs (Fig. 3A). Brain and pancreas also segregated out along the first principal component. Notably, ADAR1 expression peaked in the brain and the gonads but was lowest in the pancreas (Fig. 3B). In addition, ADAR2 expression was highest in the brain and ovary too, while ADAR3 was expressed mainly in the brain. Consistent with the ADAR expression patterns, deaminase activity as quantified by the editing index over all repeats was strongest in the ovary (Fig. 3C), which was previously observed in zebrafish as well [44]. However, the brain index appeared to be similar to that of most other organs, potentially due to inhibition by ADAR3 and other editing repressors [55]. We further calculated the editing index for individual repeat families and observed that editing activity was again strongest in the ovary for most repeat families (Fig. 3D). Moreover, satellite transcripts showed an overall highest level of editing among all known repeats, just like the results obtained from developmental datasets (Fig. 2D and Additional file 1: Fig. S10D and S12D).

Subsequently, we examined the editing of individual sites. Overall, more editing events could be detected with deeper sequencing (Additional file 1: Fig. S19A), but the brain and spleen always gave the largest number of editing sites per million mapped reads (Additional file 1: Fig. S19B), regardless of the minimum editing level set for a particular genomic position to be declared as being edited (Additional file 1: Fig. S19C). Nevertheless, considering all sites detected in at least one tissue, the median editing level in the ovary was clearly the highest (Additional file 1: Fig. S19D) because many of the sites were not edited in the brain or spleen and thus were not identified by our pipelines in these two organs. Clustering analysis also revealed that the ovary was an outlier with more highly edited sites than other tissues (Fig. 3E).

Next, we sought to study the tissue-specific sites. To this end, we filtered for positions that were covered in at least two tissues by 10 or more reads each, could be edited at a minimum level of 1%, and exhibited at least threefold higher editing rate in one particular tissue over the others. Consistently, the ovary emerged as the organ with the most tissue-specific editing sites followed by the brain (Fig. 3F). Interestingly, most tissue-specific sites were in the 3’UTR with only a small minority in intergenic regions, unlike developmental process-specific sites (Fig. 2F). This suggests that many new unannotated transcripts are expressed during embryogenesis. Importantly, tissue-specific editing is largely not due to tissue-specific expression since editing levels do not correlate well with expression levels for majority of the genes (Additional file 1: Fig. S20). For the ovary-specific sites, GO analysis revealed a functional enrichment for genes involved in histone exchange and nucleic acid phosphodiester bond hydrolysis, processes that are pertinent to meiosis, as well as endoplasmic reticulum (ER) physiology (Fig. 3G). Notably, ER stress has been found to play diverse roles in the ovary, including pathological states like the polycystic ovary syndrome [63, 64]. Additionally, GO analysis of brain-edited transcripts showed strong enrichment for genes involved in energy production (organophosphate metabolism and carbohydrate derivative metabolism), neuronal function (chemical synaptic transmission, ruffle assembly, receptor-mediated endocytosis, and actin filament organization), as well as lipid metabolism. Notably, the brain contains large amounts of sphingolipids and cholesterol, which are important for development and maintenance of the organ [65–68], while disruption of lipid metabolism and energy homeostasis are often observed in neurological diseases like amyotrophic lateral sclerosis, Alzheimer’s disease, and Parkinson’s disease. Hence, our results suggest a link between RNA editing and tissue functions.

Earlier in our study, we observed low ADAR expression in oocytes (Additional file 1: Fig. S12B), but high deaminase activity (Additional file 1: Fig. S12C) in the same samples. To resolve the apparent contradiction, we compared the editing events detected in oocytes with those identified in the ovary (Fig. 3H). Most of the editing events in the ovary were found in oocytes too. Importantly, most of the A-to-I editing sites in oocytes with adequate coverage in the ovary were also observed to be deaminated in the female gonads as well. Hence, our analyses suggest that many of the edited transcripts present in oocytes are maternally deposited, thereby accounting for the high editing index despite the relatively low ADAR expression in the germ cells.

### Analysis of RNA editing with long-read sequencing

**Fig. 4:**
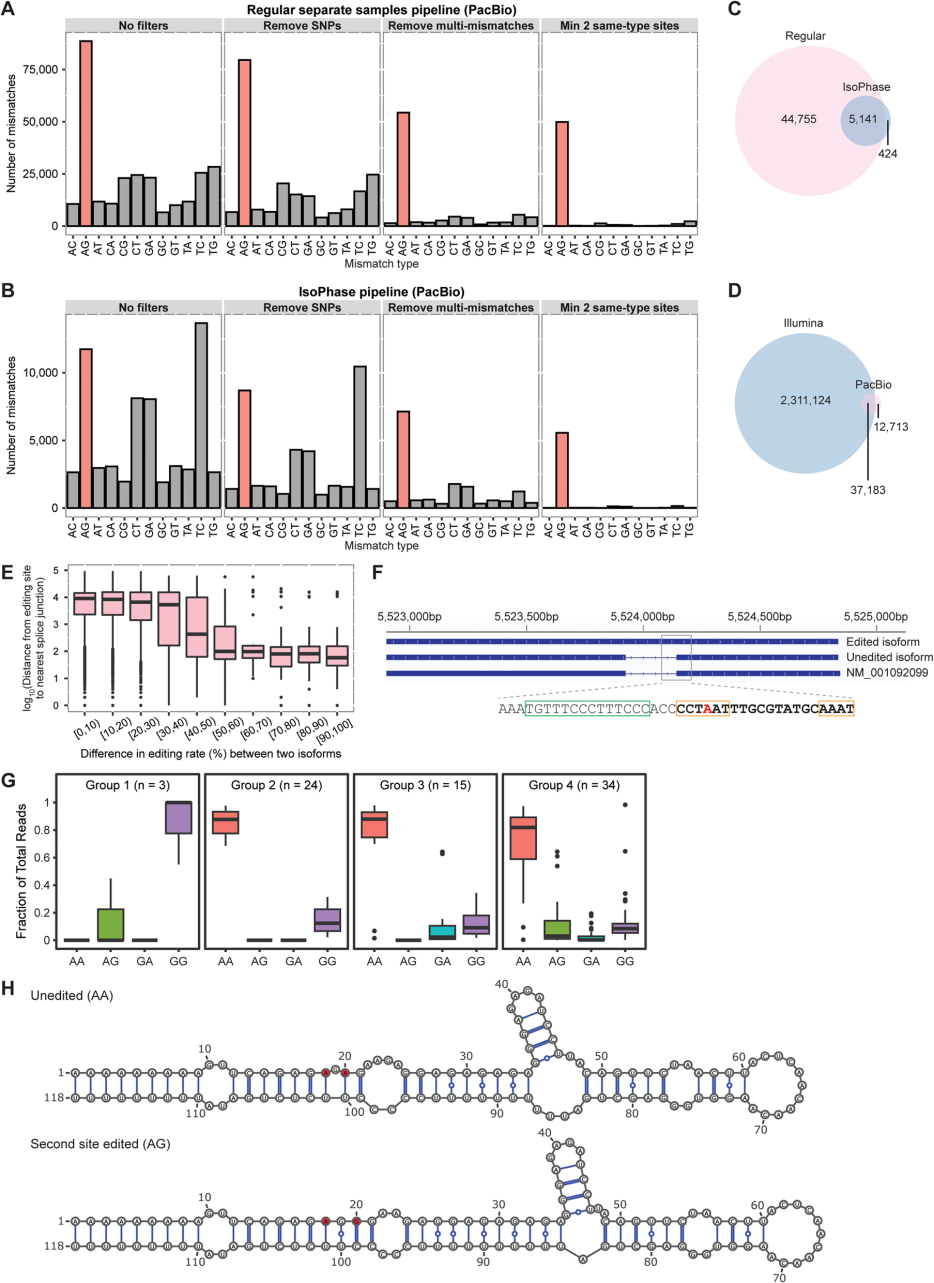
Analysis of RNA editing by long-read sequencing. **A** Distribution of mismatch types at different steps of our regular separate samples analysis workflow with REDItools. **B** Distribution of mismatch types at different steps of a modified separate samples analysis workflow where variant calling was performed with the IsoPhase tool. **C** Venn diagram comparing the number of editing sites detected by each computational pipeline. **D** Venn diagram comparing the number of editing sites identified through Illumina short-read sequencing analysis and PacBio long-read sequencing analysis. **E** Box plot showing the difference in editing rate of a site between two isoforms and the distance of that site to the closest splice junction in the gene. **F** An example in the 3’UTR of cdc27 illustrating how editing may regulate splicing. The ADAR target is at chr9_10S:5,524,147 (xenLae2) and is highlighted in red, while the 3’ splice site is at chr9_10S: 5,524,143–5,524,144. We found that the editing rate of the unspliced isoform was 25.0%, while that of the spliced isoform was 0.0%. The polypyrimidine tract is boxed in green, while the putative QKI binding site, whose consensus motif is ACUAAY-N_1–20_-UAAY, is boxed in orange. Based on studies in mammals, the consensus sequence of the 3’ splice site consists of a stretch of at least 12 pyrimidines followed by an AG dinucleotide. Here, the requisite polypyrimidine tract is followed by a non-canonical CC dinucleotide. **G** Boxplots showing the editing patterns of genes with two target sites. **H** Secondary structure predictions of the 5’UTR of xarp when it is unedited and when the downstream target site in the gene is edited. In all boxplots, the box depicts the first to last quartiles, whiskers indicate 1.5 times the interquartile range, the center line represents the median, and points represent the outliers

Studies of RNA editing are typically performed using Illumina sequencing data, but the short reads can present mapping issues, lack isoform-specific information, and does not readily permit phasing of editing events. Hence, we leveraged on PacBio single-molecule long-read sequencing to supplement our Illumina-based survey of the RNA editome in *X. laevis*. Given the high deamination activity in early development, we sequenced stage 1 and stage 9 embryonic samples on the PacBio platform and searched for editing sites using both REDItools [58] as before (Fig. 4A) and the IsoPhase tool [69] (Fig. 4B). Similar filters were imposed in both analysis pipelines to remove genomic variants, clusters of sites with multiple mismatch types, and isolated sites, which resulted in A-to-G mismatches standing out clearly as the most dominant type of RNA–DNA differences. The A-to-G percentages were 88.7% and 91.6%, while the FDRs were estimated to be 0.9% and 1.9% for REDItools and IsoPhase, respectively. Overall, IsoPhase was substantially more conservative than REDItools in variant calling, and 92.4% (5141 out of 5565 A-to-G mismatches) of A-to-I editing events detected by the former was also identified by the latter (Fig. 4C). Hence, we focused primarily on the editing sites obtained from REDItools. Altogether, our analysis of the PacBio sequencing data revealed another 12,713 editing sites that had not been identified from Illumina sequencing data so far (Fig. 4D).

Next, we sought to study the impact on splicing by ADAR-mediated editing. To this end, we plotted the difference in modification rate between any two isoforms at each edited position against the distance of that editing site from the nearest splice junction (Fig. 4E). Notably, we observed that the larger the difference in editing rate between two isoforms, the closer is the target site to a splice junction, suggesting that A-to-I editing may promote or suppress the formation of a specific isoform. For example, an editing site was detected in the 3’UTR of the cdc27 gene, which could form a dsRNA structure with bulges (Fig. 4F and Additional file 1: Fig. S21). Our PacBio data showed that unedited RNA molecules were spliced to give the annotated transcript, while edited molecules remained unspliced to produce another transcript with a longer 3’UTR in the mature mRNA. Examination of the genomic sequence around the splice junction revealed a polypyrimidine tract important for spliceosome assembly [70] followed by a putative motif for the alternative splicing regulator QKI [71, 72], which overlapped with the editing site; ADAR-mediated editing would disrupt an invariant adenosine within the core of the motif (CUAA), and potentially prevent QKI binding and splicing from taking place.

A-to-I editing sites often occur in clusters. Hence, we asked how different editing sites in the same transcript could influence one another. To address the question, we focused mainly on genes containing two edited positions each, since the number of combinations would grow exponentially large with bigger cluster sizes. We found that 76 genes exhibited significant association in editing between both their sites (*P* < 0.01, *χ*^2^-test). Subsequently, hierarchical clustering revealed that these genes could be separated into four groups (Fig. 4G and Additional file 1: Fig. S22). In the first group, both sites were highly amenable to editing with most sequencing reads showing a “G” at both positions, unlike the other clusters where most sequencing reads were unedited at both positions. Next, in the second group, the two sites could not be individually deaminated and instead appeared to be edited together or not at all. Finally, in the third and fourth groups, one of the sites was more readily deaminated, which then seemed to promote the editing of the other position. An example of the fourth group was observed in the 5’UTR of the xarp gene, where two editing sites resided close to each other (Fig. 4H). Although both sites were flanked by guanosines, the nucleotide 3’ of the first site was predicted to be mismatched, which might prevent it from being edited. However, when the second site was deaminated, the mismatched guanosine could then form a wobble base pair with uracil, potentially allowing the first site to be edited as well. Collectively, our results suggest that in *Xenopus*, A-to-I editing may influence other RNA metabolic processes like splicing and editing events in the same transcript are frequently not independent of one another like what has been previously observed in other metazoans [73, 74].

### RNA editing landscape in *X*. *tropicalis*

Although *X. laevis* is the first frog species to be widely used for biological research, its relatively long life cycle and allotetraploid genome make genetic and genomic analyses more challenging [1]. Hence, *X. tropicalis* was introduced in the 1990s as an alternative vertebrate model organism. Over the years, it has gained increasing popularity due to its shorter generation time and diploid genome.

**Fig. 5:**
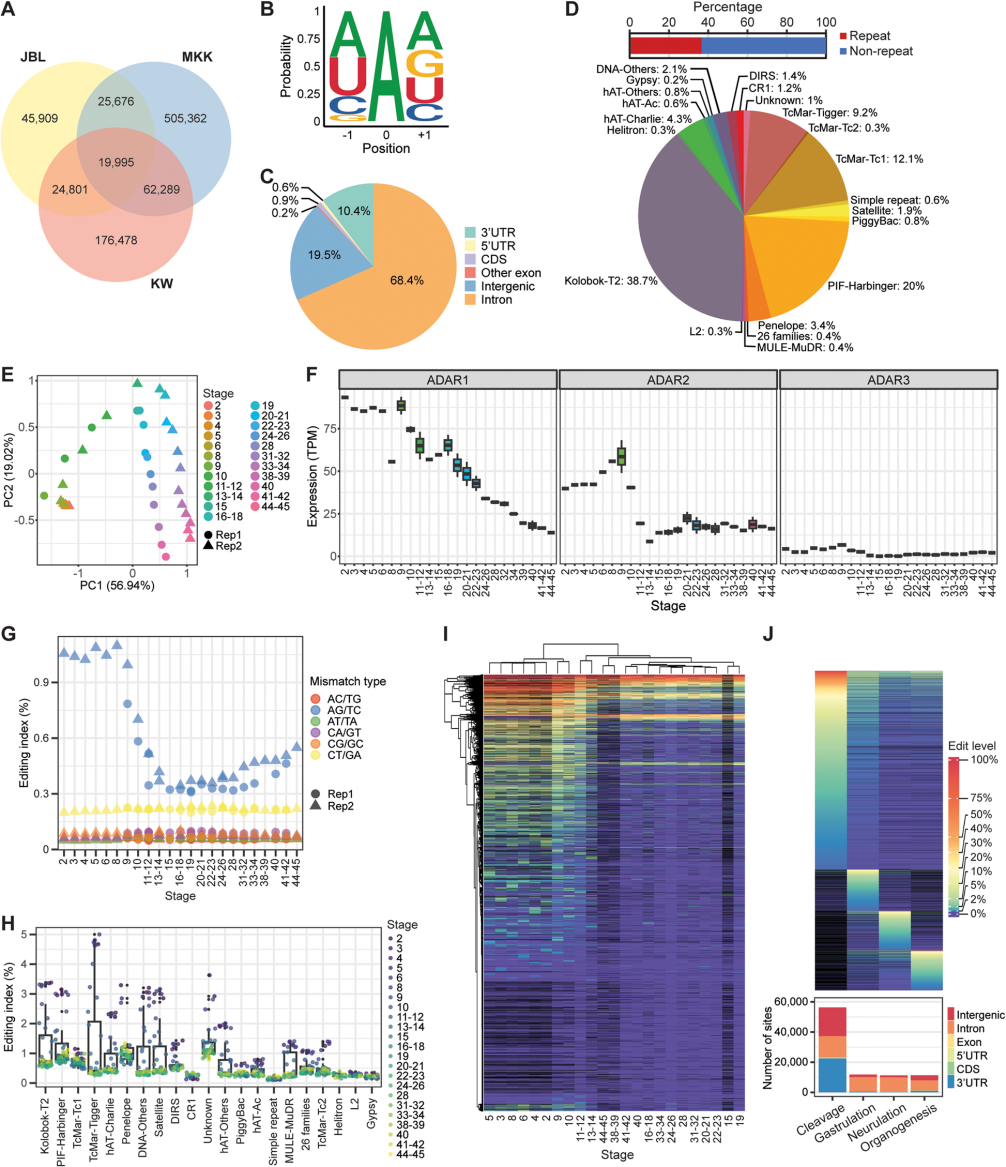
RNA editing landscape in *X. tropicalis*. **A** Venn diagram showing the number of A-to-I editing sites found in three different studies, which are indicated by the initials of the last authors. JBL refers to the datasets reported by Jin Billy Li [14], KW refers to the datasets reported by Karl Wotton [75], and MKK refers to the datasets reported by Mustafa K. Khokha [76]. **B** ADAR motif in *X. tropicalis* based on our curated list of editing sites. **C** Genomic locations of editing sites in *X. tropicalis*. Expectedly, most editing events were found in non-coding regions of the genome. An appreciable percentage also lie within intergenic regions. **D** Editing in repetitive regions of the *X. tropicalis* genome. Like *X. laevis*, minority of the ADAR targets in *X. tropicalis* were found in repeats. The pie chart shows the distribution of A-to-I editing sites in various repeat families. Twenty-six annotated repeat families contained comparatively few editing events and thus were grouped together in a single slice of the pie chart. **E** PCA plot based on gene expression values from the JBL study showing segregation of embryonic samples according to developmental stages. **F** Transcript levels of ADAR enzymes across development in the JBL study. The ADAR expression values are provided in Additional File 2. **G** Global editing index measured across all repeat families in the JBL study. **H** Editing index for each individual repeat family in the JBL study. While ADAR activity was variable across the repeat families, it was consistently higher during the beginning stages of development regardless of repeat type. Twenty-six annotated repeat families contained comparatively few editing events and thus were grouped together for calculation of the index. **I** Hierarchical clustering of editing levels. Each row is a different editing site, while each column is a different developmental stage interrogated in the JBL study. **J** Many transcriptomic loci were targeted by ADARs in only a single developmental process. Top: Heatmap depicting the editing rates of these process-specific sites. Bottom: Genomic locations of the process-specific sites

Here, we sought to characterize the A-to-I RNA editome in *X. tropicalis* to complement our analyses in *X. laevis*. To this end, we analyzed our previously published Illumina RNA-seq data on *X. tropicalis* embryogenesis [14] as well as RNA-seq datasets from another two studies [75, 76]. After mapping to the reference genome (xenTro9), we identified editing sites using the same computational pipelines as before, namely separate samples approach with REDItools, pooled samples approach with REDItools, and the hyper-editing methodology (Additional file 1: Fig. S23-S26). DNA SNPs were called from publicly available WGS data for *X. tropicalis* [11, 77–79] and filtered off. In every study, the A-to-G percentage was over 80% for each pipeline with a corresponding FDR of 5% or less. We again observed that many coding sites were eliminated by the last filter requiring at least two same-type sites as editing in coding regions tends to be site-selective (Additional file 1: Fig. S27), necessitating the recovery of isolated events. Altogether, 860,510 editing loci were uncovered from the three studies (Fig. 5A). The motif for *X. tropicalis* sites was similar to that for *X. laevis* sites, with a depletion of G upstream but no obvious enrichment of G downstream of the target adenosine (Fig. 5B). Expectedly, most sites were found in non-coding sequences (Fig. 5C) and most targeted genes could be deaminated at multiple positions (Additional file 1: Fig. S28). Additionally, consistent with our earlier observations in *X. laevis*, majority of the editing events did not occur in annotated repeat regions of the genome (Fig. 5D) and among the repeat sites, a substantial percentage (38.7%) resided in Kolobok-T2 elements, which could fold into long dsRNA structures (Additional file 1: Fig. S29).

Subsequently, we examined the RNA editing landscape in *X. tropicalis* over development. Expression-based PCA of our previously published data [14] showed that the embryonic samples segregated by developmental progression as expected (Fig. 5E). Like *X. laevis*, ADAR1 and ADAR2 transcript levels were substantially higher in earlier than later developmental stages, while ADAR3 was barely expressed during embryogenesis (Fig. 5F). Correspondingly, editing activity as measured by the overall repeat editing index was clearly elevated in earlier embryos before declining after zygotic genome activation (Fig. 5G). Across individual repeat families that were annotated, the editing index varied appreciably with Penelope and Harbinger elements being the most highly targeted in *X. tropicalis* (Fig. 5H). This is unlike *X. laevis* where satellite repeats were most extensively edited instead. Nevertheless, editing activity in each repeat type was consistently strongest earlier in development. We further inspected individual editing sites at every stage. Substantially more editing events could be detected in early-stage samples than later-stage samples regardless of sequencing depth and the activity threshold used to call editing sites (Additional file 1: Fig. S30). Clustering analysis also showed that the early embryonic stages grouped together and displayed a more active editing profile than later stages (F ig. 5I). Similar results were observed with data from another developmental study (MKK) (Additional file 1: Fig. S31-S34). Consequently, we found considerably more cleavage-specific editing events than the other three processes, which were predominantly in introns, 3’UTRs, and intergenic regions (Fig. 5J). Taken together, our results unveil the strong deaminase activity present during the beginning stages of *Xenopus* development, suggesting that the ADAR enzymes may play a major role in regulating the maternal-to-zygotic transition in frogs.

### Comparison of A-to-I editing across *Xenopus* species

**Fig. 6:**
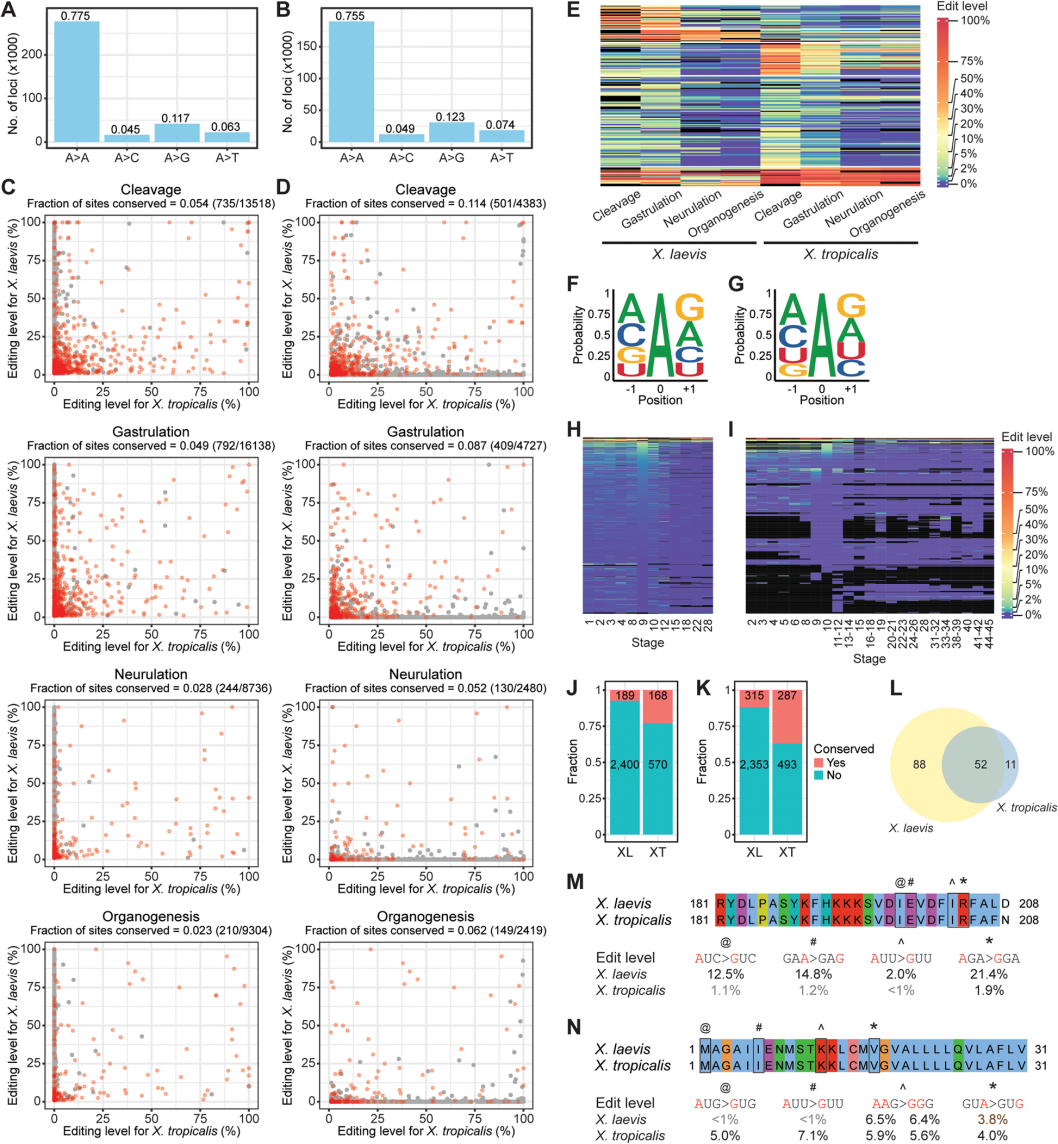
Conservation of RNA editing between *X. laevis* and *X. tropicalis*. **A** Nucleotide identity of genomic loci in *X. tropicalis* that had been lifted over from our list of editing sites in *X. laevis*. **B** Nucleotide identity of genomic loci in *X. laevis* that had been lifted over from our list of editing sites in *X. tropicalis*. **C** Scatterplots showing the modification rates of curated *X. laevis* editing sites and the corresponding lifted over positions in *X. tropicalis*. Only sites found in the same genes or gene families in both frog species were plotted here. **D** Scatterplots showing the modification rates of curated *X. tropicalis* editing sites and the corresponding lifted over positions in *X. laevis*. Only sites found in the same genes or gene families in both frog species were plotted here. **E** Heatmap showing the editing patterns of sites that exhibited a difference in deamination rate of at least 10% between *X. laevis* and *X. tropicalis* in some developmental process. **F** ADAR motif of high-confidence coding sites in *X. laevis*. **G** ADAR motif of high-confidence coding sites in *X. tropicalis*. **H** Self-organizing map of editing rates in the MHT study. Each row is a different high-confidence coding site, while each column is a different developmental stage of *X. laevis*. **I** Self-organizing map of editing rates in the JBL study. Each row is a different high-confidence coding site, while each column is a different developmental stage of *X. tropicalis*. **J** High-confidence coding sites identified separately in *X. laevis* (XL) or *X. tropicalis* (XT) were mostly not found in the other species. The number of conserved sites is not identical between the two frog species because two homeologous genes (one on the L chromosome and one on the S chromosome) can be targeted in *X. laevis* for each gene that is edited in *X. tropicalis*. **K** The high-confidence list was expanded with coding sites that were filtered off due to isolation or the presence of other mismatch types, but there was still an overall lack of conservation in editing between *X. laevis* (XL) and *X. tropicalis* (XT). **L** Comparison of genes whose protein-coding regions were edited in *X. laevis* or *X. tropicalis*. **M** Alignment of partial METTL5 protein sequences from *X. laevis* and *X. tropicalis*, with the targeted amino acid residues boxed in black and the corresponding codon changes indicated below. The mettl5 gene was edited at four positions in *X. laevis*, but at only one position in *X. tropicalis*. **N** Alignment of partial WLS protein sequences from *X. laevis* and *X. tropicalis*, with the targeted amino acid residues boxed in black and the corresponding codon changes indicated below. The wls gene was edited at five positions in *X. tropicalis*, but at only two positions in *X. laevis*. For the fifth position, although its modification rate in *X. laevis* was comparable to that in *X. tropicalis*, it was not in the high-confidence list of coding sites for *X. laevis* because there were only two variant reads, which did not pass our threshold of three variant reads

The extensive RNA-seq data on the two model frog species provided us with an opportunity to evaluate how the editing landscape varies within the *Xenopus* genus. To this end, we lifted over the *X. laevis* sites to the *X. tropicalis* genome and vice versa using chain files (Additional file 1: Fig. S35-S36). Most editing sites could not be lifted over likely because greater than 90% of the unconvertable sites were found in introns and intergenic regions, which are often poorly conserved across species. The poorly assembled state of both genomes could also have contributed to the low liftover rate. Of the lifted over sites, majority maintained an A in the target genome, with a small fraction already containing a G fixed in the DNA (Fig. 6A-B). We then compared the deamination rates of individual editing sites between the two *Xenopus* species at different developmental processes, focusing mainly on the positions that remained as an A in the genome after conversion. Unexpectedly, most sites appeared to be species-specific, being edited only in the original frog (Additional file 1: Fig. S37). To assess the accuracy of the chain files, we inspected the mapping between the two genome assemblies (Additional file 1: Fig. S35-S36). Under 10% of the matched loci were in genes with conflicting symbols, so we discarded them. However, most of the remaining editing events still occurred only in the original frog (Additional file 1: Fig. S38). Subsequently, we removed sites in intergenic regions or uncharacterized genes with generic names but again observed that even matched loci found in the same genes or gene families were largely species-specific in their editing (Fig. 6C-D). Moreover, many sites that were conserved between *X. laevis* and *X. tropicalis* exhibited distinct editing patterns over development (Fig. 6E).

To confirm the highly species-specific nature of A-to-I editing in the *Xenopus* genus, we converted the editing sites in *X. laevis* or *X. tropicalis* to the transcriptome of *X. andrei*, an octoploid frog [80], and asked if the corresponding positions were modified in this third species. Most of the mapped positions in the *X. andrei* transcriptome were also adenosines (Additional file 1: Fig. S39A-B). However, due to the sparse sequencing coverage and our requirement of at least 10 reads to ascertain the editing level of a site, we could only examine a few thousand positions in the *X. andrei* transcriptome (Additional file 1: Fig. S39C-D). Even then, there was still a lack of editing at most of these lifted over positions in the octoploid frog across development (Additional file 1: Fig. S39E-F). Taken together, our results indicate that editing events are largely not conserved across *Xenopus* species.

### Coding sites in *Xenopus*

Editing events in protein-coding regions can generate unique isoforms and diversify the proteome, but their detection has always been challenging as they are much fewer in numbers than those in non-coding regions. Almost all previous searches for editing events in protein-coding regions have yielded large numbers of false positive sites [81], and special care must be taken to accurately identify bona fide coding sites. Hence, we checked the set of coding variants identified by our different pipelines and found that they were not predominantly A-to-G mismatches (Additional file 1: Fig. S40-S41). Instead, all four transitions were detected at high levels in our regular workflow with REDItools, indicating that the list of coding sites was contaminated by many DNA mutations and SNPs, since transition mutations in the genome are produced at a much higher frequency than transversions. Additionally, we observed persistent A-C and T-G mismatches from hyper-editing analysis, which might be caused by misalignment of reads due to reduction in the number of bases for mapping.

To address the issue, we focused on editing events that were detected by both our regular workflow with REDItools and hyper-editing analysis. We rationalized that in stable dsRNA structures, multiple adenosines were likely to be targeted by ADARs in a promiscuous manner but only a few of them were expected to be deaminated in a site-selective manner. These primary sites would be present in sequencing reads without other variants and thus could be mapped to the original genome and would also be present in hyper-edited reads and thus could only be mapped to the transformed genome. Encouragingly, when we examined such coding variants, we found a clear enrichment of A-to-G mismatches for all studies (Additional file 1: Fig. S42-S43). The A-to-G percentage ranged from 80.9 to 98.6% for studies with stranded RNA-seq libraries and 43.2 to 67.5% for studies with non-stranded libraries. We also observed clear enrichment of T-to-C mismatches for the studies with non-stranded libraries, suggesting that there might be considerable unannotated antisense transcripts emerging from the coding regions. In contrast, the highest G-to-A percentage among all the studies was only 4.3%, indicating high detection accuracy of coding sites. Hence, we classified the A-to-G mismatches that were identified through both regular read alignment and hyper-editing analysis as our set of high-confidence coding sites. To further augment this list for each frog species, we included all other coding sites in the same genes, many of which were detected through hyper-editing analysis, as well as sites whose orthologous positions were deemed to be high-confidence site in the other species. In total, we identified 3244 and 1034 highly accurate coding sites in *X. laevis* and *X. tropicalis*, respectively.

Subsequently, we examined these high-confidence sites in greater detail. Inspection of the neighboring nucleotides revealed that while G was still disfavored one base upstream of the target adenosine, it was now enriched one base downstream like the preferred sequence motif of ADAR in human, mouse, and zebrafish [31–36, 39, 44, 59] (Fig. 6F-G). Additionally, the coding sites were edited more prominently in early embryogenesis compared to later developmental stages, although the deamination rates were generally low (Fig. 6H-I and Additional file 1: Fig. S44-S45). Nonetheless, 642 and 260 sites could still be modified at a rate of at least 10% in *X. laevis* and *X. tropicalis*, respectively (Additional file 1: Fig. S46A, S47A). Majority of editing events in coding regions yielded amino acid changes (Additional file 1: Fig. S46B, S47B), highlighting their potential to diversify the proteome, and most targeted genes could undergo both non-synonymous and synonymous editing (Additional file 1: Fig. S46C, S47C). The two most common types of recoding events in *X. laevis* and *X. tropicalis* involved altering a lysine to either arginine or glutamate (Additional file 1: Fig. S46D-E, S47D-E). Unsurprisingly, due to the way we identified high-confidence sites, the bulk of the targeted genes contained two or more coding sites (Additional file 1: Fig. S46F, S47F), as exemplified by the tmem62 gene in *X. laevis*, which harbored a cluster of 14 editing sites in the last coding exon before the stop codon that could all be edited at a level higher than 10% (Additional file 1: Fig. S46G), and the crkl gene in *X. tropicalis*, which harbored five major sites in the last coding exon with maximum deamination rates greater than 10% (Additional file 1: Fig. S47G).

Next, we investigated the conservation of high-confidence coding sites between *Xenopus* species. Most editing events in the protein-coding regions of *X. laevis* were not detected in *X. tropicalis* and vice versa (Fig. 6J). Notably, they were not identified in the second species mostly due to a lack of variant reads and not because of insufficient sequencing coverage (Additional file 1: Fig. S48). It is possible that some bona fide sites might have been prematurely discarded in our workflows. Hence, we recovered sites that were filtered off in one *Xenopus* species either because they were isolated or because other types of mismatches were present in the vicinity but were deemed to be high-confidence in the other species. However, even with these rescued sites, most coding events were still not conserved between *X. laevis* and *X. tropicalis* (Fig. 6K). We then examined the editing levels of the conserved sites but found that they were largely modified to different extents between the two frogs (Additional file 1: Fig. S49 and Additional file 3). Interestingly, despite the lack of conservation at the site level, we observed a larger overlap in editing between *X. laevis* and *X. tropicalis* at the gene level (Fig. 6L). Nevertheless, although the same genes could be edited within their coding regions in both frogs, they were typically modified more extensively in one species. For example, in the mettl5 gene, four coding sites in *X. laevis* but only one in *X. tropicalis* were identified, and even the single common site was more strongly deaminated in *X. laevis* (Fig. 6M). In contrast, the wls gene in *X. tropicalis* contained more ADAR targets near the start of the coding sequence than that in *X. laevis* (Fig. 6N). Taken together, our results reveal divergent A-to-I RNA editing landscapes in the two frogs in both non-coding and coding regions of the genome.

### Conservation of editing between *Xenopus* and mammals

**Fig. 7:**
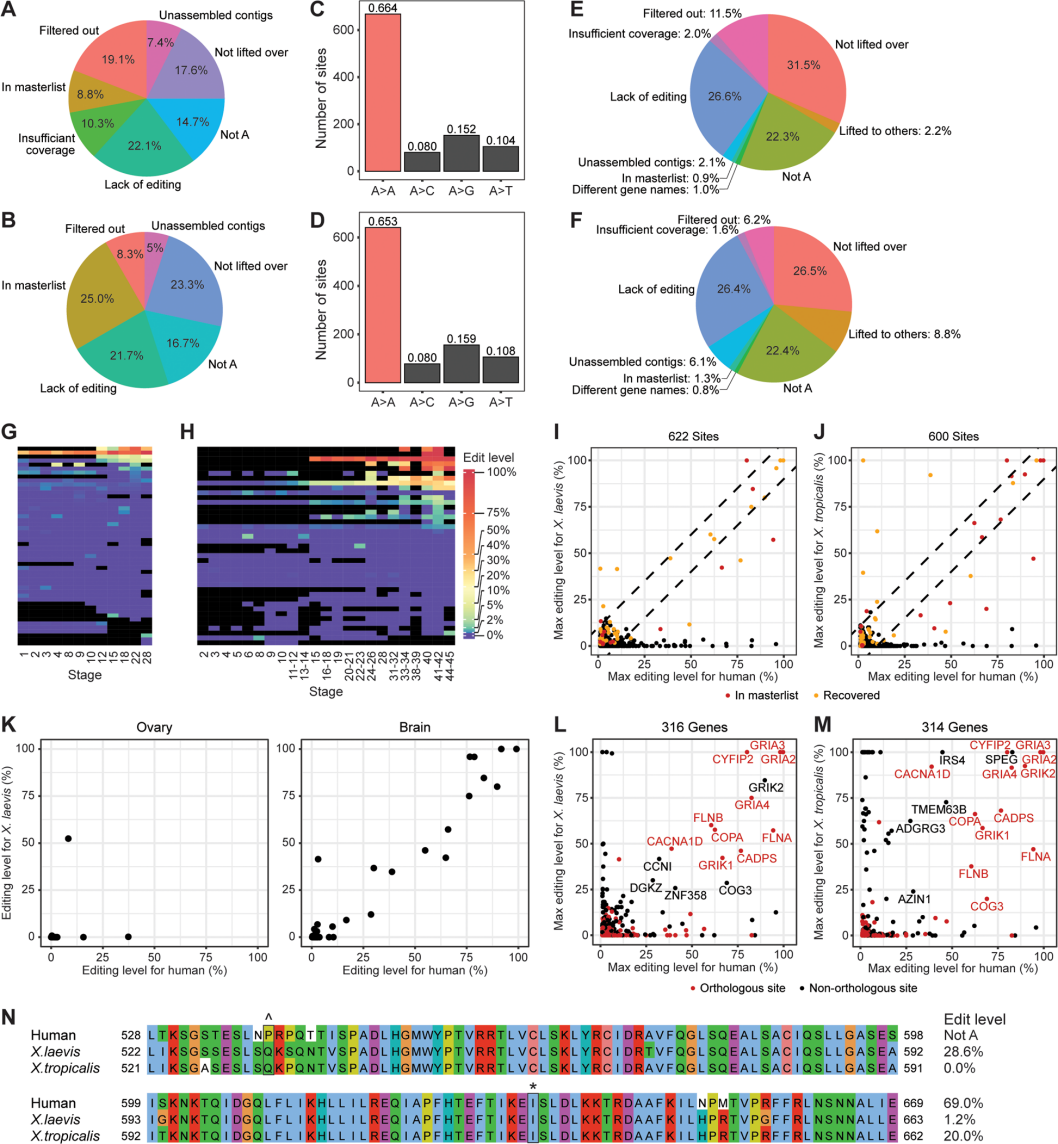
Evaluation of mammalian editing sites in *Xenopus*. **A** Assessment of 58 conserved mammalian ADAR targets in *X. laevis*. The mammalian targets were lifted over from both the human and mouse genome assemblies and those that could not be converted were largely found in non-coding regions. Additionally, one mammalian site could give rise to two lifted over positions in *X. laevis* because the human sequence mapped to one homeolog while the mouse sequence mapped to the other homeolog. **B** Assessment of 58 conserved mammalian ADAR targets in *X. tropicalis*. **C** Nucleotide identity of genomic loci in *X. laevis* that had been successfully converted from our recently published list of human coding sites. **D** Nucleotide identity of genomic loci in *X. tropicalis* that had been successfully converted from our recently published list of human coding sites. **E** Pie chart summarizing our analysis of the 1517 human coding sites in *X. laevis*. **F** Pie chart summarizing our analysis of the 1517 human coding sites in *X. tropicalis*. **G** Self-organizing map of editing rates in the MHT study. Each row is a different vertebrate conserved coding site, while each column is a different developmental stage of *X. laevis*. **H** Self-organizing map of editing rates in the JBL study. Each row is a different vertebrate conserved coding site, while each column is a different developmental stage of *X. tropicalis*. **I** Scatterplots showing the modification rates of human coding sites and the corresponding lifted over positions in *X. laevis*. Sites found in genes with conflicting symbols between the two species or with insufficient sequencing coverage were omitted. Dotted lines indicate 10% difference in editing between human and *Xenopus*. **J** Scatterplots showing the modification rates of human coding sites and the corresponding lifted over positions in *X. tropicalis*. **K** Scatterplots showing the modification rates of conserved coding sites in two adult tissues of human and *X. laevis*. Each plotted site was covered by at least 10 sequencing reads per sample. **L** Scatterplots showing the maximum editing levels of coding DNA sequence (CDS)-targeted genes in human and their counterparts in *X. laevis*. **M** Scatterplots showing the maximum editing levels of CDS-targeted genes in human and their counterparts in *X. tropicalis*. Some well-known ADAR substrates were edited in human and both *Xenopus* species, such as transcripts encoding subunits of the glutamate receptor. However, several well-characterized mammalian ADAR targets were not edited in both frogs, such as the serotonin 5-HT2C receptor. **N** Alignment of partial COG3 protein sequences from human, *X. laevis*, and *X. tropicalis*, with the targeted amino acid residues boxed in black. Notably, the cog3 gene was differentially edited between the three species. The first editing event occurs only in *X. laevis* and is silent as there is no change in amino acid, while the second editing event is a conserved mammalian ADAR target and converts an isoleucine codon to a valine codon. Curiously, the second site is edited at much lower levels in *Xenopus*

Previous work has shown that only a handful of RNA editing events are conserved in mammals [82]. Of these conserved mammalian sites, 1 is a C-to-U editing site targeted by APOBEC1, while the remaining 58 are A-to-I editing sites targeted by ADARs. We asked if the 58 mammalian ADAR targets could also be found in frogs; 54.4% and 61.7% could not be lifted over, were not encoded by a genomic A, or lacked editing in the transcriptome of *X. laevis* and *X. tropicalis*, respectively (Fig. 7A-B). Moreover, only a quarter or less were present in our list of editing sites for either frog, indicating poor conservation between mammals and *Xenopus*. Nevertheless, some of the missing sites were originally detected by REDItools but were filtered off due to low variant coverage or editing rate, presence of other mismatch type in the vicinity, or because the editing events were isolated in a single study. Additionally, *Xenopus* genomes are not completely assembled to date. We did not analyze the hundreds of loose contigs earlier due to the intensive compute resources needed. A few of the mammalian editing sites could be recovered from these unassembled contigs. In total, we found 15 and 19 of the conserved mammalian sites in *X. laevis* and *X. tropicalis*, respectively (Additional file 4).

We recently curated a comprehensive list of 1517 human editing sites in the coding region [81]. To determine if they were targeted in frogs as well, we lifted them over to the *X. laevis* or *X. tropicalis* genome using chain files. Majority of the human sites were successfully mapped to corresponding protein-coding loci in the frog (Additional file 1: Fig. S50A-B). Some sites mapped to intergenic regions, but they might contain unannotated coding exons. Hence, we proceeded with both groups of lifted over sites. Of these, around two-thirds maintained an A in the *Xenopus* genome, while around 15% were already encoded by a G in the DNA (Fig. 7C-D). We focused on the loci with a genomic A and also omitted from further analysis a small number of sites that had conflicting gene symbols between human and frog (Additional file 1: Fig. S50C-D). Only a few human coding sites were present in our lists of ADAR targets for *X. laevis* and *X. tropicalis*, and about a quarter of them had sufficient sequencing coverage at the lifted over positions but lacked evidence of editing (Fig. 7E-F). Nevertheless, a notable percentage (6–12%) had been detected by REDItools but did not pass the filters in our pipelines, while another set of human sites had been lifted over to the loose contigs. Some of these editing events could be recovered as they showed evidence of editing at a rate of at least 1% in the frog. Altogether, we identified 54 and 56 of the human coding sites in *X. laevis* and *X. tropicalis*, respectively (Additional file 5). Interestingly, unlike our previous results, editing of these conserved sites mostly occurred in later developmental stages (Fig. 7G-H and Additional file 1: Fig. S51), including the well characterized GRIA2 gene whose editing increases during mammalian brain development [83] and is essential for proper nervous system function [84, 85].

Subsequently, we compared the editing rates between human and frog. Expectedly, most of the matched genomic positions with adequate sequencing coverage lie along the x-axis since they were not targeted in either *Xenopus* species (Fig. 7I-J). Notably, out of all the human coding sites that could be found in the frog, several were differentially edited by over 10% between the two genera. We then inspected the modification rates of the conserved sites in the ovary and brain, which stood out in our earlier analysis of adult organs. Remarkably, while editing was poorly correlated between human and frog ovary (*r* = 0.155), it was very well correlated between human and frog brain (*r* = 0.963) (Fig. 7K). Moreover, among all the adult organs, editing of the conserved coding sites was most readily detected in the brain of *X. laevis* (Additional file 1: Fig. S52). These results suggest that brain coding sites are highly conserved during the evolution of vertebrates, while editing events in ovary and early embryogenesis tend to be innovations of individual species.

We further examined editing at the transcript level since some genes were targeted by ADAR at multiple sites. Specifically, we asked what the maximal editing level observed in each gene was. By and large, many of the transcripts that were strongly deaminated in both human and *Xenopus* were well-known ADAR substrates, including various ion channels and filamins (Fig. 7L-M). Interestingly, however, the most highly edited position in some genes were not identical between human and frog, as exemplified by cog3 (Fig. 7N). Collectively, our results indicate that although some functionally important editing events are conserved between mammals and *Xenopus*, there is also species-specific adaptation of editing in protein-coding sequences.

## Discussion

ADAR-mediated RNA editing is a fundamental post-transcriptional gene regulatory mechanism with critical roles in vertebrate development and physiology. However, much of our understanding of its functions are derived from a few model organisms like mouse [37–39] and zebrafish [44, 45]. Hence, the extent to which ADAR functions and editing events are conserved between different vertebrate species is still not fully understood. Here, we comprehensively mapped the A-to-I RNA editing landscape in both *X. laevis* and *X. tropicalis*. We constructed the editing atlas by examining multiple RNA-seq datasets from six different studies, including ours, using various computational pipelines such as separate samples and pooled samples approaches and hyper-editing analysis. Expectedly, we uncovered hundreds of thousands of A-to-I editing sites in each species mostly within non-coding regions of the genome. Like zebrafish [44], editing activity was highest in the cleavage stages of development and decreases after zygotic genome activation. Furthermore, a substantial number of edited transcripts in the 1-cell zygote was likely to be maternally deposited. Curiously however, ADAR activity in mammals increases over the course of development instead [83, 86, 87]. The reason for this difference is currently unknown. It is unlikely to arise during the split of tetrapods from fishes in the evolutionary tree. Instead, we speculate that the need for RNA editing during early embryogenesis might depend on whether development occurs inside or outside the female’s body. Most fishes and amphibians are oviparous, while majority of mammals are viviparous. When embryos develop outside the body, they are exposed to the environment and RNA editing might help them to respond to unexpected changes in conditions like how it enables insects and cephalopods to adapt to their surroundings [88–91].

A-to-I editing and splicing are known to be closely intertwined during the processing of RNA transcripts in the nucleus [25–28]. Indeed, we observed that numerous genes in *Xenopus* underwent both editing and alternative splicing over the course of development (Fig. 2I). Using long-read sequencing, we also found that the larger the difference in modification rate between two isoforms, the closer was the editing site to a splice junction (Fig. 4E), suggesting a regulatory effect of editing on splicing in frogs as well. Future work can involve deciphering the precise mechanism of this regulation in different genes and its phenotypic consequences.

Our work highlights the challenges in identifying editing sites in protein-coding sequences. Previously, we leveraged on thousands of RNA-seq datasets from the GTEx consortium to curate 1517 coding sites in human [81]. Due to the large number of datasets, we could filter out mismatches present in only a few donors to achieve a high detection accuracy. However, the number of datasets available for *Xenopus* is only a small fraction of that for human and the number of unique mating pairs is even less. Consequently, there was minimal improvement in the detection accuracy when we tried to require sites to be present in multiple individuals or clutches (data not shown). Furthermore, frogs are outbred with poorly annotated SNPs, unlike the inbred mice that are used in biological studies. The unknown DNA variants can serve to confound RNA editing analysis. We also tried to correct for mis-mapping with BLAT, but the A-to-G percentage did not increase appreciably (data not shown). Consequently, we could only confidently identify a smaller set of coding sites that are presumably in stable dsRNAs. We envision that as more genomic studies are performed on *Xenopus* in the future, the greater availability of WGS and RNA-seq datasets will enable additional coding sites to be detected accurately in the frog.

An interesting result that emerged from our analyses was the apparent lack of conservation in editing across *Xenopus* species. Such a result might have been expected in non-coding sequences, which are poorly conserved in general. Indeed, we observed that editing within repetitive elements was species-species. Transcripts derived from satellite DNA showed an overall highest level of editing in *X. laevis* (Fig. 2D), whereas Penelope and Harbinger elements were most strongly targeted in *X. tropicalis* (Fig. 5H). Nevertheless, we also discovered that protein-coding regions in *X. laevis* and *X. tropicalis* were differentially edited by ADAR to a large extent as well. Majority of our high-confidence sites were not conserved between the two species (Fig. 6J-K), and even the commonly targeted sites or genes were often more extensively modified in one species than the other (Fig. 6M-N and Additional file 1: Fig. S49). The only conserved coding sites that were strongly edited in both *X. laevis* and *X. tropicalis* were well-known ADAR substrates, such as ion channels and filamins, which were found in human too (Fig. 7L-M). The divergent RNA editomes suggest that individual frog species might have co-opted A-to-I editing for their own unique physiologies.

## Conclusions

In summary, the work presented here has deepened our understanding of RNA editing in vertebrates. Not only do we have a comprehensive atlas of A-to-I editing in *Xenopus*, but we can also better appreciate the diversity of editing in different species. Furthermore, the resources we have generated can aid in future studies of the immune and non-immune roles of ADAR-mediated editing in developmental patterning, tissue homeostasis, and diseased states.

## Methods

### Isolation of total RNA

RNA was extracted from *Xenopus laevis* embryos as follows. The embryos were lysed in 200 µL lysis buffer (250 µg/mL proteinase K, 0.5% SDS, 5 mM EDTA, 50 mM Tris pH 7.5, and 50 mM NaCl) with 5 µL RNaseOUT. After centrifugation at 8000 g for 5 min at 4℃, 100 µL lysis buffer was added to 100 µL supernatant followed by 200 µL acid phenol: chloroform. After 2 min of incubation at room temperature, the mixture was centrifuged at 12,000* g* for 15 min at 4℃. The colorless upper aqueous layer was transferred to a new tube and 200 µL isopropanol was added. After 10 min incubation at room temperature, RNA was pelleted by centrifugation at 12,000* g* for 10 min at 4℃. The RNA pellet was washed twice with 500 µL of 70% ethanol. The dried pellet was resuspended in 45 µL RNase-free water. The residual DNA was digested by adding 5 µL DNase I buffer and 1 µL DNase I (New England Biolabs). After 1 h incubation at 37℃, the reaction was cleaned up using RNA Clean and Concentrator (Zymo Research).

### Isolation of genomic DNA

Genomic DNA was extracted from *Xenopus* embryos as follows. After lysis and centrifugation (as above), the mixture was extracted with 200 µL of 1:1 phenol:chloroform. The top aqueous layer was transferred to a new tube and extracted again with 200 µL chloroform. The DNA in the top aqueous layer was precipitated using 0.3 M sodium acetate and 0.6 volume of isopropanol. The mixture was incubated at − 20℃ for 30 min and then centrifuged at maximum speed for 10 min. The pellet was washed once with 70% ethanol and resuspended in TE buffer.

### Preparation of Illumina sequencing libraries

Illumina libraries were prepared with kits from New England Biolabs. RNA-seq libraries were prepared using NEBNext UltraII Directional RNA library Prep kit according to manufacturer’s protocol. For WGS libraries, 2 μg of genomic DNA was first diluted to 50 μL using TE buffer before the DNA was sheared to roughly 300 bp using Covaris with the following settings: intensity 5, 10% duty cycle, 200 cycles per burst, and 50 s treatment time. The fragmented DNA was then prepared for Illumina sequencing using NEBNext UltraII DNA library Prep Kit according to manufacturer’s protocol.

### Analysis of Illumina RNA-seq data

#### Identification of SNPs in WGS data

Raw reads were trimmed with fastp (v0.20.0) with the following parameters to remove adapters and low-quality reads: –detect_adapter_for_pe \ –qualified_quality_phred 25 \ –unqualified_percent_limit 10 \ –length_required 50 \ –trim_poly_x \ –correction. Trimmed reads that passed were mapped to the UCSC xenLae2 (for *X. laevis*) or xenTro9 (for *X. tropicalis*) genome with BWA-MEM (v0.7.17) using default settings [92]. Next, variants were called from the reads that mapped to chromosomes with FreeBayes (v1.3.4) using default settings [93] and filtered for Depth ≥ 10 and Qual ≥ 20.

#### De novo* identification of editing sites in X. laevis and X. tropicalis*

We used REDItools2 to detect the editing sites [94]. RNA-seq reads were trimmed with fastp (v0.20.0) with the following parameters to remove adapters and low-quality reads: –detect_adapter_for_pe \ –qualified_quality_phred 25 \ –unqualified_percent_limit 10 \ –length_required 50 \ –trim_poly_x \ –correction. Trimmed reads that passed were mapped to the UCSC xenLae2 (for *X. laevis*) or xenTro9 (for *X. tropicalis*) genome with STAR aligner (v2.7.8a) using the following parameters: –genomeLoad NoSharedMemory \ –outReadsUnmapped Fastx \ –outSAMtype BAM SortedByCoordinate \ –outSAMstrandField intronMotif \ –outSAMattributes All \ –readFilesCommand zcat \ –outFilterType BySJout \ –outFilterMultimapNmax 1 \ –alignSJoverhangMin 8 \ –alignSJDBoverhangMin 1 \ –outFilterMismatchNmax 999 \ –outFilterMismatchNoverLmax 0.04 \ –alignIntronMin 20 \ –alignIntronMax 1,000,000 \ –alignMatesGapMax 1,000,000. Only reads that mapped to the chromosomes were taken for downstream analysis. Library strandedness was verified with infer_experiment.py from RSeQC. The BAM files were then analyzed using REDItools2 with the following parameters: -q 30 -bq 30 -s (0 for unstranded, 1 for second stranded, and 2 for first stranded). For the separate samples pipeline, the BAM files of each sample in a study were parsed by REDItools2 independently. For the pooled samples pipeline, the BAM files of all samples in a study were combined into a single file before parsing by REDItools2. For both pipelines, genomic positions must have at least 10 × coverage, 3 variant reads, and a minimally 1% mismatch rate (number of variant reads divided by total coverage multiplied by 100) from the REDItools2 output table to be further analyzed. The following filters were applied sequentially to extract high-confidence editing sites: (1) remove SNP positions identified from WGS, (2) remove sites where another nearby (± 20 nt window) candidate site is of a different mismatch type, and (3) require that sites be accompanied by at least one other same-mismatch-type site (± 20 nt window). To rescue isolated sites (i.e., sites that did not have at least one other same-mismatch-type site within the ± 20 nt window), mismatches that passed the second filter were required to have at least 5 variant reads and appear in at least two studies.

#### Detection of hyper-editing sites

Unmapped reads were extracted for detection using a previously published computational pipeline [56]. Default parameters were used. BAM files generated by the pipeline were subsequently parsed by REDItools2.

#### Editing level of sites in developmental processes

Cleavage comprises stages 0 to 8 or 0 to 6.5 hpf; gastrulation comprises stages 8.5 to 13 or 7 to 16 hpf; neurulation comprises stages 14 to 20 or 16.5 to 22 hpf; and organogenesis comprises stages 21 to 40 or 22.5 to 66 hpf. For *X. laevis*, the editing rate of a site within each developmental stage was first determined by taking the average editing rate across all studies (i.e., MHT, MK, and DR). A minimum coverage of 10 reads was required for an editing level measurement to be counted. The modification rate of a site for each developmental process was then determined by taking the average across all stages within that process. For *X. tropicalis*, samples in the MKK study were collected by hpf, while those in the JBL study were collected by developmental stages. Hence, the editing rate of a site within each stage was not calculated. Instead, the editing level for each developmental process was calculated by taking the average editing rate from all relevant stages or hpf within that process. Again, a minimum coverage of 10 reads was required for an editing level measurement to be counted.

#### Gene expression analysis

Gene expression levels were quantified with Salmon with default parameters. The transcriptome reference sequence was generated by GffRead [95] from genome reference and annotations, which were retrieved from the UCSC Genome Browser. The R package tximport was used to convert transcript-level to gene-level abundance [96]. Raw TPM values were transformed using rv transformation [97], while batch correction was performed with limma [98]. Inverse transformation of post-batch corrected and transformed TPM values was then performed to obtain gene expression values. To determine the expression of genes containing process-specific sites, we averaged their expression values across all developmental stages within that process.

#### Alternative splicing analysis

MAJIQ software was used to identify alternative splicing events using default parameters [61]. Any alternative splicing events with percent spliced in (PSI) less than 1% were not considered.

#### Gene Ontology analysis

The topGO R package was used to perform enrichment analysis. GO terms were retrieved from Xenbase [99]. Mapping of mRNA accession numbers to GO terms was performed using db2db from bioDBnet [100]. For all GO queries, the background used was the set of genes expressed in the same biological context being analyzed.

#### RNA structure analysis

RNAfold in the ViennaRNA Package 2.0 was utilized to predict RNA secondary structures [101]. Subsequently, the VARNA applet (version 3–93) was used to draw and edit the predicted structures [102].

### Analysis of PacBio long-read sequencing data

Consensus reads were obtained from the isoseq3 workflow, after which they were mapped to the UCSC xenLae2 reference genome using minimap2 -ax map-pb. Isoforms from the Pacbio reads were also detected using the default isoseq3 workflow. Subsequently, Sam2Tsv and in-house scripts were used to extract total coverage and variant coverage for positions in each isoform. The same filters as described above (for REDItools2) were applied to obtain editing sites.

### Cross-species analysis of editing

#### X. laevis versus X. tropicalis

CrossMap [103] was used to lift over editing sites between *X. laevis* (XL) and *X. tropicalis* (XT) using chain files obtained from the UCSC Genome Browser [104]. Only sites that appeared as “A” in the genome of both species were considered for downstream analysis. For XL-to-XT mapping, we restricted one unique XL site to one unique XT site. For XT-to-XL mapping, we allowed up to two XL sites from one XT site, but both XL sites must come from the same chromosome, one.L and one.S. Additionally, different gene names between XL and XT were double-checked against NCBI gene database and Xenbase using geneSynonym (https://github.com/oganm/geneSynonym) to reassign genes with different annotations but were likely to be orthologous. Gene names that contained LOC/XB/MGC/XM/NM/XR/c_orf_ were classified as general names.

#### Comparison of X. laevis/X. tropicalis with X. andrei

Since a genome reference is unavailable for *X. andrei*, a previously published Trinity-assembled transcriptome reference of *X. andrei* [80] was mapped to xenLae2 (for *X. laevis*) or xenTro9 (for *X. tropicalis*) using minimap2 -ax splice. Only transcripts with a uniquely mapped region in the *X. laevis* or *X. tropicalis* genome were used for downstream analysis. The RNA-seq reads of *X. andrei* were also mapped to the Trinity-assembled transcriptome reference using bowtie2 with default parameter. Sam2Tsv was then used to extract the total coverage and base coverage of each position that corresponded to an editing site in the two model *Xenopus* species.

#### Evaluation of mammalian editing sites in Xenopus

Human and mouse editing sites were converted to coordinates in the *X. laevis* and *X. tropicalis* genome assemblies using UCSC LiftOver3 utility. Reference genomes (hg19, hg38, mm9, mm10, xenLae2, and xenTro9) and RefSeq annotations were obtained from the UCSC Genome Browser [104]. All sites were checked for their reference base in the various genome sequences using the faidx command from SAMtools (version 1.17) [105]. Those without A as reference base were discarded. Lifted over coordinates were annotated with their respective gene and gene type using the intersect command from the BEDTools-2.30.0 suite [106] against the respective RefSeq annotations for each species. To ascertain if sites were transferred between orthologous genes, gene symbols were appended with their respective full gene descriptions sourced from the NCBI database using ESearch [107], which were then compared using in-house R scripts and manual inspection.

### Supplementary Information

**Additional file 1: Fig. S1 **Identification of RNA editing sites in *X. laevis* using publicly available Illumina RNA-seq data and our regular separate samples pipeline.** Fig. S2 **Distribution of mismatch types for recovered isolated sites in *X. laevis* identified using our regular separate samples pipeline.** Fig. S3 **Identification of RNA editing sites in *X. laevis* using publicly available Illumina RNA-seq data and our regular pooled samples pipeline.** Fig. S4 **Distribution of mismatch types for recovered isolated sites in *X. laevis* identified using our regular pooled samples pipeline.** Fig. S5 **Number of potential A-to-I editing sites in non-coding or coding regions after each step of filtering for all the *X. laevis* studies.** Fig. S6 **Identification of RNA editing sites in *X. laevis* using publicly available Illumina RNA-seq data and the hyper-editing pipeline.** Fig. S7 **Examples of dsRNA structures in repetitive regions of the *X. laevis* transcriptome.** Fig. S8 **Number of ADAR target sites per gene in *X. laevis*.** Fig. S9 **Individual A-to-I editing sites in our embryogenesis data. **Fig. S10 **ADAR expression and activity in embryogenesis data from the MK study.** Fig. S11 **Individual A-to-I editing sites in embryogenesis data from the MK study.** Fig. S12 **ADAR expression and activity in embryogenesis data from the DR study.** Fig. S13 **Individual A-to-I editing sites in embryogenesis data from the DR study.** Fig. S14 **Comparison of gene expression levels in *X. laevis* across studies. **Fig. S15 **Comparison of editing levels in *X. laevis* across studies. **Fig. S16 **Evaluation of different fold changes.** Fig. S17 **Correlation between editing levels of developmental process-specific sites and expression levels of the host genes.** Fig. S18 **Examples illustrating positive and negative relationships between A-to-I editing and gene expression. **Fig. S19 **Individual A-to-I editing sites in tissue data from the DR study.** Fig. S20 **Correlation between editing levels of tissue-specific sites and expression levels of the host genes.** Fig. S21 **Structure of the 3’UTR of cdc27. **Fig. S22 **Hierarchical clustering of genes containing two sites whose editing was significantly associated with each other (P < 0.01, χ^2^-test).** Fig. S23 **Identification of A-to-I editing sites in *X. tropicalis* using RNA-seq data from the JBL study, where a total of 40 samples were analysed.** Fig. S24 **Identification of A-to-I editing sites in *X. tropicalis* using RNA-seq data from the MKK study, where a total of 193 samples were analysed. **Fig. S25 **Identification of A-to-I editing sites in *X. tropicalis* using RNA-seq data from the KW study, where a total of 6 samples were analysed.** Fig. S26 **Distribution of mismatch types for recovered isolated sites in *X. tropicalis* identified using our regular (A) separate samples or (B) pooled samples pipeline. **Fig. S27 **Number of potential A-to-I editing sites in non-coding or coding regions after each step of filtering for all the *X. tropicalis* studies.** Fig. S28 **Number of ADAR target sites per gene in *X. tropicalis*.** Fig. S29 **Examples of dsRNA structures in repetitive regions of the *X. tropicalis* transcriptome. **Fig. S30 **Individual A-to-I editing sites in embryogenesis data from the JBL study. **Fig. S31 **Gene expression analysis of embryogenesis data from the MKK study.** Fig. S32 **ADAR activity in embryogenesis data from the MKK study. **Fig. S33 **Individual A-to-I editing sites in embryogenesis data from the MKK study.** Fig. S34 **Hierarchical clustering of editing levels from the MKK study.** Fig. S35 **Examination of *X. laevis* editing sites in *X. tropicalis*. **Fig. S36 **Examination of *X. tropicalis *editing sites in *X. laevis*.** Fig. S37 **Cross-species comparison of editing rates for all matched sites. **Fig. S38 **Cross-species comparison of editing rates, omitting matched sites in genes with conflicting symbols.** Fig. S39 **Evaluation of *X. laevis* or *X. tropicalis* editing sites in *X. andrei*.** Fig. S40 **Distribution of mismatch types for coding regions in *X. laevis*.** Fig. S41 **Distribution of mismatch types for coding regions in *X. tropicalis*. **Fig. S42 **Types of coding mismatches identified by both regular read alignment and REDItools and the hyper-editing pipeline in *X. laevis*.** Fig. S43 **Types of coding mismatches identified by both regular read alignment and REDItools and the hyper-editing pipeline in *X. tropicalis*.** Fig. S44 **Developmental editing profiles of coding sites in *Xenopus. ***Fig. S45. **Profiles of high-confidence coding sites edited at 5% or higher in *Xenopus*.** Fig. S46 **High-confidence coding sites in *X. laevis*. **Fig. S47 **High-confidence coding sites in *X. tropicalis*.** Fig. S48 **Reasons for sites found in one species not detected in the other species.** Fig. S49 **Scatterplot showing the editing levels of high-confidence conserved coding sites in *Xenopus*.** Fig. S50 **Examination of human CDS sites in *Xenopus*.** Fig. S51 **Developmental editing profiles of conserved coding sites in *Xenopus*. **Fig. S52 **Editing profile of conserved coding sites in adult tissues of *X. laevis*.

**Additional file 2. **Expression values of ADAR1, ADAR2, and ADAR3 in all analyzed samples.

**Additional file 3. **Protein-coding sites that are conserved between *X. laevis* and *X. tropicalis*.

**Additional file 4. **Conserved mammalian sites that are also edited in *Xenopus*.

**Additional file 5. **Human coding sites that are also edited in *Xenopus*.

## Data Availability

All data generated or analyzed during this study are included in this published article, its supplementary information files, and publicly available repositories. The Illumina and PacBio sequencing data generated in this study have been deposited in the NCBI Sequence Read Archive (SRA) under BioProject accession number PRJNA952800 [108]. Additionally, the following RNA-seq datasets were downloaded from NCBI SRA: PRJNA296953 [109] and PRJNA296921 [110] for the DR study, PRJNA298254 [111] and PRJNA298393 [112] for the MK study, PRJNA160141 [113] for the JBL study, PRJNA275011 [114] for the MKK study, PRJNA846095 [115] for the KW study, as well as PRJNA437641 [116] for *X. andrei*. Furthermore, the following WGS datasets were downloaded from NCBI SRA: PRJNA313213 [117] for *X. laevis* as well as PRJNA357404 [118], PRJNA577946 [119], and PRJNA642750 [120] for *X. tropicalis*. Individual ADAR expression values are provided in Additional File 2.

## Abbreviations

A: Adenosine
ADAR: Adenosine deaminase acting on RNA
AEI: Alu editing index
C: Cytidine
CDS: Coding DNA sequence
dsRNA: Double-stranded RNA
ER: Endoplasmic reticulum
FDR: False discovery rate
G: Guanosine
GO: Gene ontology
I: Inosine
miRNA: MicroRNA
PCA: Principal component analysis
SINE: Short interspersed nuclear element
SNP: Single nucleotide polymorphism
T: Thymidine
UTR: Untranslated region
WGS: Whole genome sequencing
