# Supplementary material for: Deep transcriptome profiling reveals limited conservation of A-to-I RNA editing in Xenopus
Source: BMC Biol. 2023 Nov 9;21:251. doi: 10.1186/s12915-023-01756-2 (PMC10636886; doi:10.1186/s12915-023-01756-2)
Supplement: Supplementary file 1 — Additional file 1: Fig. S1 Identification of RNA editing sites in X. laevis using publicly available Illumina RNA-seq data and our regular separate samples pipeline. Fig. S2 Distribution of mismatch types for recovered isolated sites in X. laevis identified using our regular separate samples pipeline. Fig. S3 Identification of RNA editing sites in X. laevis using publicly available Illumina RNA-seq data and our regular pooled samples pipeline. Fig. S4 Distribution of mismatch types for recovered isolated sites in X. laevis identified using our regular pooled samples pipeline. Fig. S5 Number of potential A-to-I editing sites in non-coding or coding regions after each step of filtering for all the X. laevis studies. Fig. S6 Identification of RNA editing sites in X. laevis using publicly available Illumina RNA-seq data and the hyper-editing pipeline. Fig. S7 Examples of dsRNA structures in repetitive regions of the X. laevis transcriptome. Fig. S8 Number of ADAR target sites per gene in X. laevis. Fig. S9 Individual A-to-I editing sites in our embryogenesis data. Fig. S10 ADAR expression and activity in embryogenesis data from the MK study. Fig. S11 Individual A-to-I editing sites in embryogenesis data from the MK study. Fig. S12 ADAR expression and activity in embryogenesis data from the DR study. Fig. S13 Individual A-to-I editing sites in embryogenesis data from the DR study. Fig. S14 Comparison of gene expression levels in X. laevis across studies. Fig. S15 Comparison of editing levels in X. laevis across studies. Fig. S16 Evaluation of different fold changes. Fig. S17 Correlation between editing levels of developmental process-specific sites and expression levels of the host genes. Fig. S18 Examples illustrating positive and negative relationships between A-to-I editing and gene expression. Fig. S19 Individual A-to-I editing sites in tissue data from the DR study. Fig. S20 Correlation between editing levels of tissue-specific sites and expression levels of the hos [file 12915_2023_1756_MOESM1_ESM.pdf]

Supplementary Information

Deep transcriptome profiling reveals limited conservation of A-to-I RNA editing in *Xenopus*

Tram Anh Nguyen[1,2,12], Jia Wei Joel Heng[1,2,12], Yan Ting Ng[1,3], Rui Sun[1,2], Shira Fisher[4], Gokce Oguz[2], Pornchai Kaewsapsak[2,5], Shifeng Xue[6,7], Bruno Reversade[2,6,8,9], Adaikalavan Ramasamy[2], Eli Eisenberg[10], Meng How Tan[1,2,11,*]

[1]School of Chemistry, Chemical Engineering and Biotechnology, Nanyang Technological University, Singapore, Singapore

[2]Genome Institute of Singapore, Agency for Science Technology and Research, Singapore, Singapore

[3]School of Biological Sciences, Nanyang Technological University, Singapore, Singapore

[4]The Mina and Everard Goodman Faculty of Life Sciences, Bar-Ilan University, Ramat Gan, Israel,

[5]Department of Biochemistry, Faculty of Medicine, Chulalongkorn University, Bangkok, Thailand

[6]Institute of Molecular and Cell Biology, Agency for Science Technology and Research, Singapore, Singapore

[7]Department of Biological Sciences, National University of Singapore, Singapore, Singapore

[8]Yong Loo Lin School of Medicine, National University of Singapore, Singapore, Singapore

[9]Department of Medical Genetics, School of Medicine (KUSoM), Koç University, Istanbul, Turkey

[10]Raymond and Beverly Sackler School of Physics and Astronomy, Tel Aviv University, Tel Aviv, Israel

[11]HP-NTU Digital Manufacturing Corporate Lab, Nanyang Technological University, Singapore, Singapore

[12]These authors contributed equally to this work.

[*]Correspondence: mh.tan@ntu.edu.sg

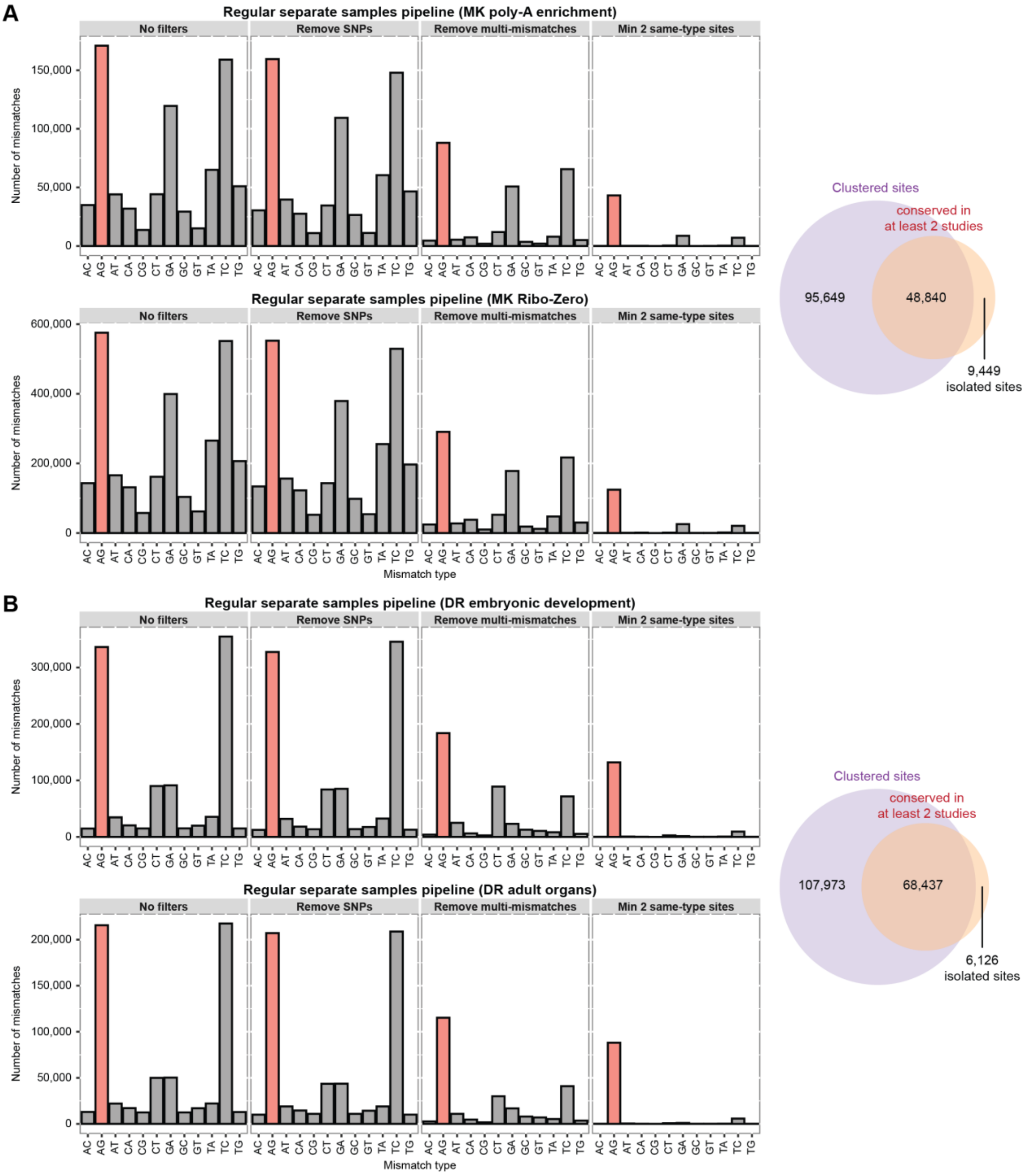
A
Regular separate samples pipeline (MK poly-A enrichment)
No filters
Remove SNPs
Remove multi-mismatches
Min 2 same-type sites
Number of mismatches
150,000
100,000
50,000
0
AC AG AT CA CG CT GA GC GT TA TC TG
Regular separate samples pipeline (MK Ribo-Zero)
600,000
400,000
200,000
Mismatch type
Clustered sites
conserved in at least 2 studies
95,649
48,840
9,449 isolated sites
B
Regular separate samples pipeline (DR embryonic development)
300,000
200,000
100,000
Regular separate samples pipeline (DR adult organs)
200,000
150,000
100,000
50,000
107,973
68,437
6,126 isolated sites

Supp. Fig. S1 Identification of RNA editing sites in *X. laevis* using publicly available Illumina RNA-seq data and our regular separate samples pipeline.

(A) In the MK study, poly(A)-selected RNA and rRNA-depleted libraries were sequenced. After variant calling with REDItools, we removed genomic SNPs and variants with other types of mismatches in their vicinity. To further enhance detection accuracy of ADAR targets, we selected for clustered sites. Nevertheless, to recover isolated sites, we required them to be present in at least two different studies. The Venn diagram shows the number of A-to-I sites identified from the MK study that are in clusters or detected in at least two studies. A total of 18 poly(A)-selected RNA samples and 18 rRNA-depleted samples were analysed.

(B) In the DR study, 28 embryonic samples at different developmental stages and 28 adult tissue samples were analysed. The sequencing datasets were processed in the same manner as those from the other studies.

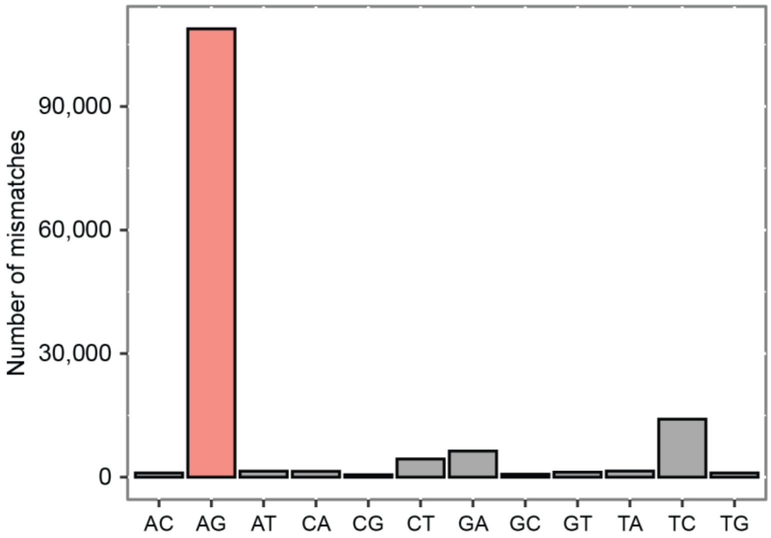

Supp. Fig. S2 Distribution of mismatch types for recovered isolated sites in *X. laevis* identified using our regular separate samples pipeline.

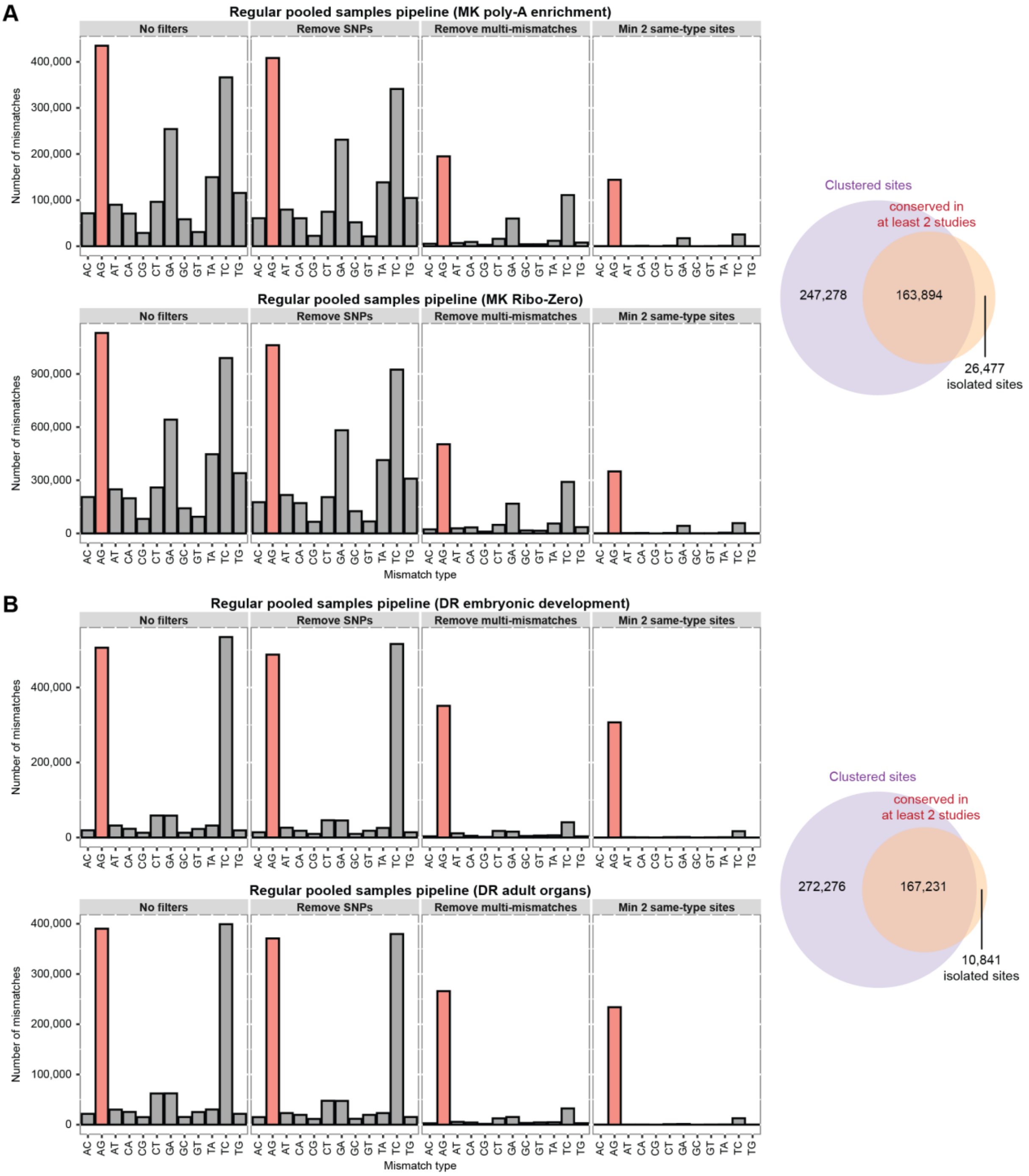

Supp. Fig. S3 Identification of RNA editing sites in *X. laevis* using publicly available Illumina RNA-seq data and our regular pooled samples pipeline.

(A) Analysis of poly(A)-selected RNA and rRNA-depleted libraries from the MK study.

(B) Analysis of data on embryos and adult tissues from the DR study.

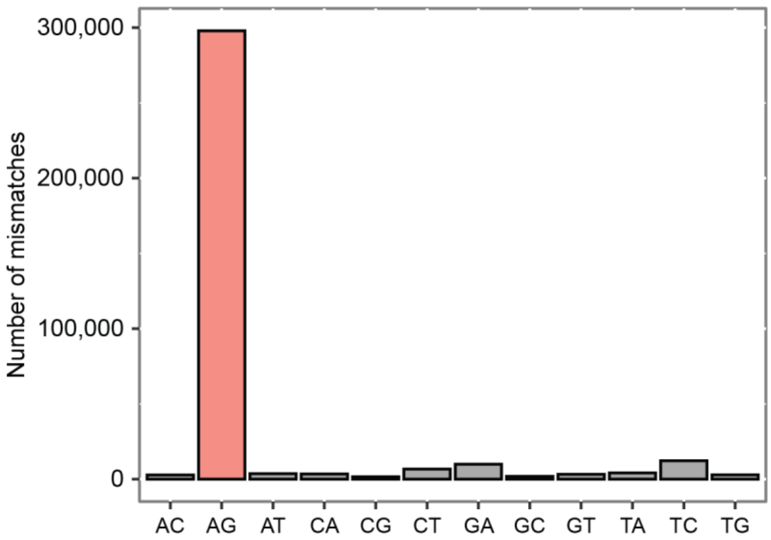

Supp. Fig. S4 Distribution of mismatch types for recovered isolated sites in *X. laevis* identified using our regular pooled samples pipeline.

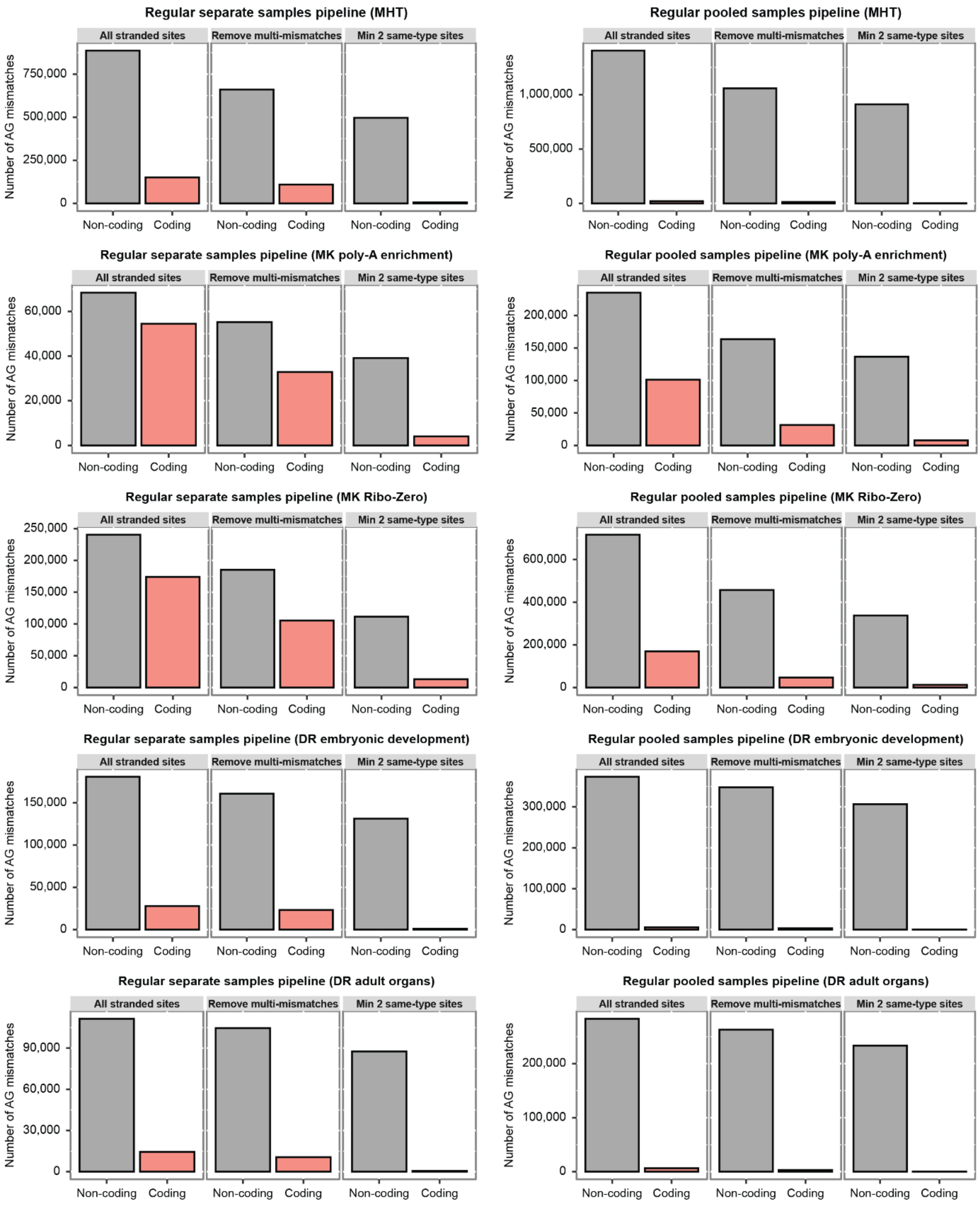

Supp. Fig. S5 Number of potential A-to-I editing sites in non-coding or coding regions after each step of filtering for all the *X. laevis* studies.

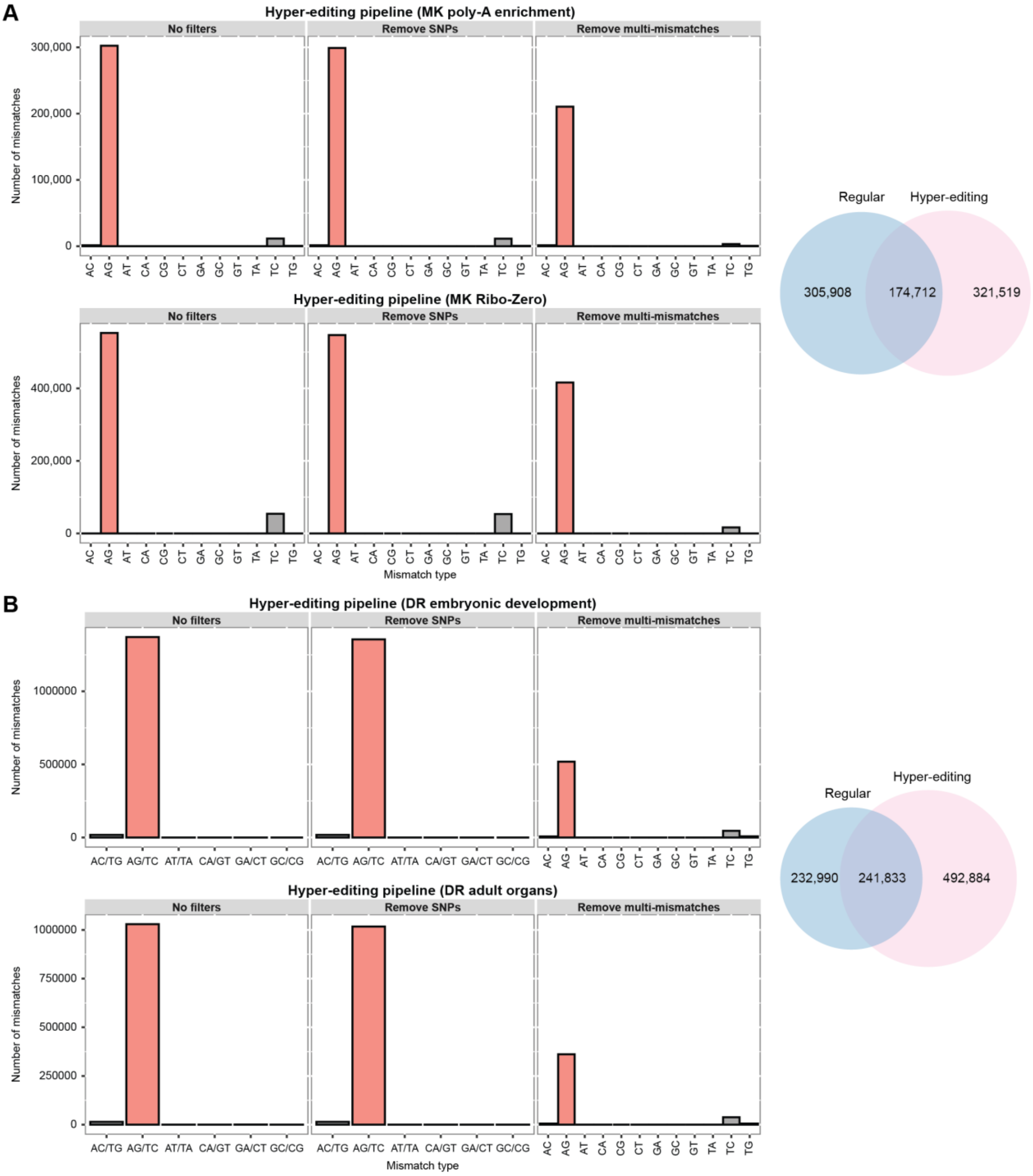

Supp. Fig. S6 Identification of RNA editing sites in *X. laevis* using publicly available Illumina RNA-seq data and the hyper-editing pipeline.

(A) Analysis of poly(A)-selected RNA and rRNA-depleted libraries from the MK study.

(B) Analysis of data on embryos and adult tissues from the DR study.

Venn diagrams show the number of editing events detected in each study using regular read alignment and REDItools or the hyper-editing pipeline where mapping was done with all As converted to Gs.

A B

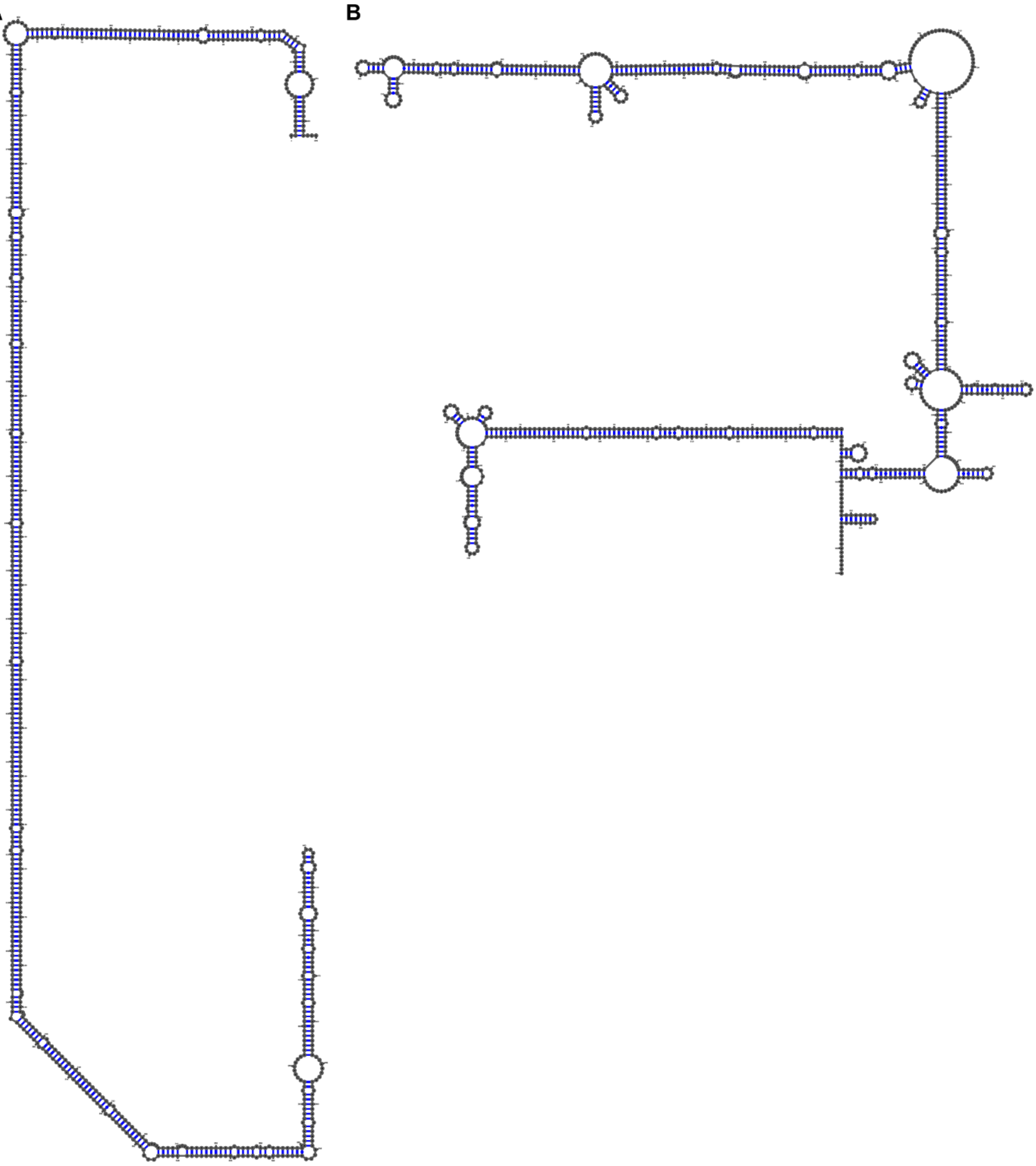

Supp. Fig. S7 Examples of dsRNA structures in repetitive regions of the *X. laevis* transcriptome.

RNAfold was used to predict the secondary structures of Kolobok elements in the **(A)** ndufc2 and **(B)** cox6a genes in *X. laevis*.

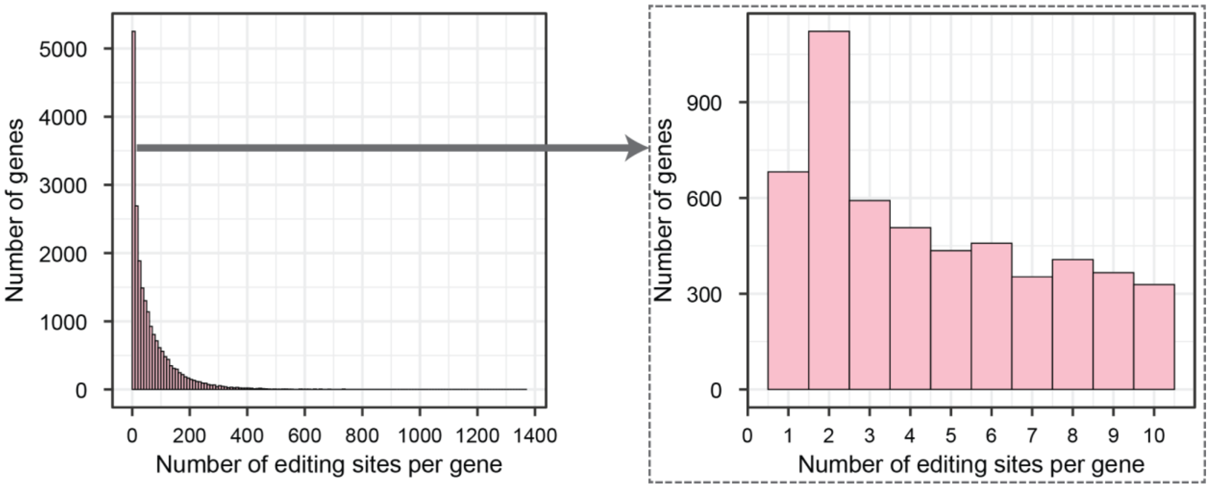

Supp. Fig. S8 Number of ADAR target sites per gene in *X. laevis*.

Histograms showing the distribution of editing site counts per gene in *X. laevis*. The left histogram has a bin size of 10, while the right histogram is a zoomed-in graph of the first bin.

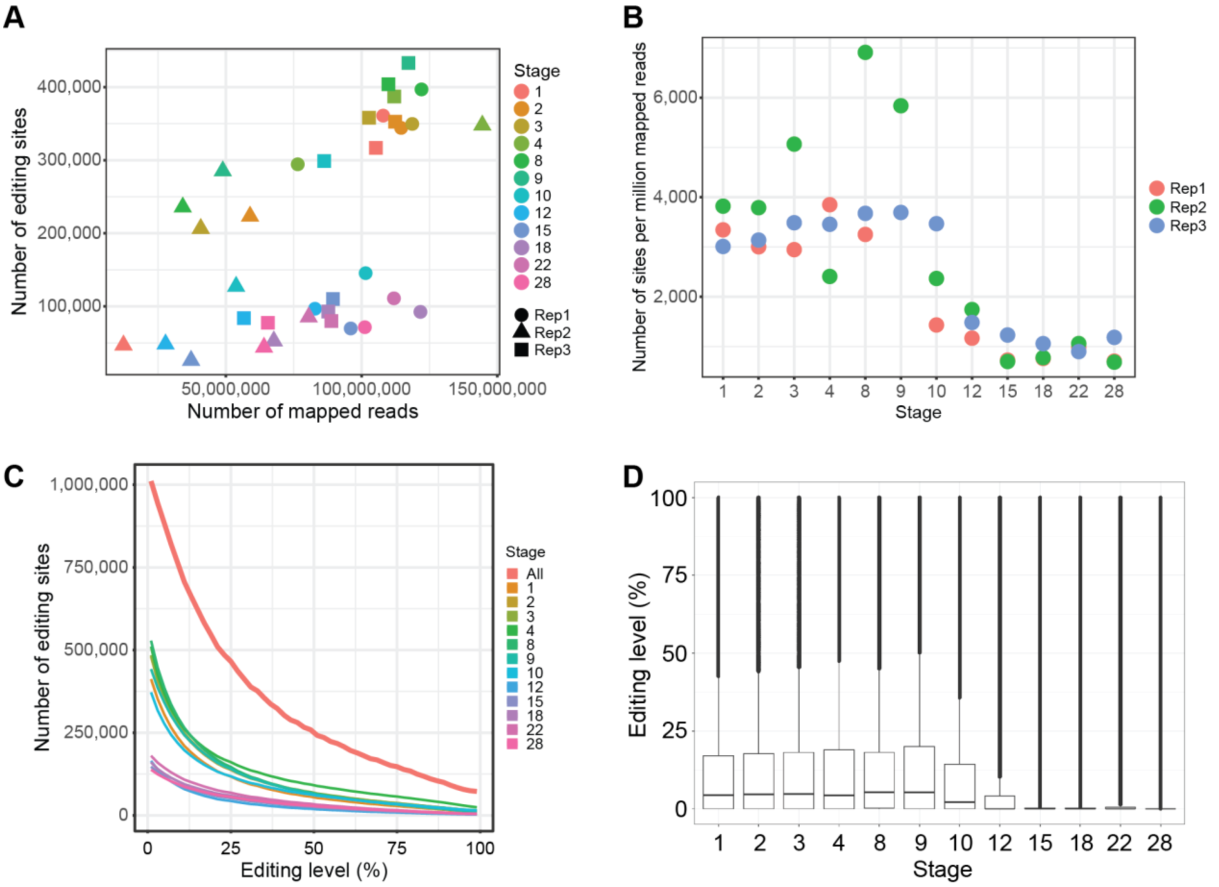

Supp. Fig. S9 Individual A-to-I editing sites in our embryogenesis data.

(A) Scatterplot showing the number of editing events detected in and the sequencing depth of each dataset.

(B) Graph showing the number of editing events detected at each developmental stage normalized by the sequencing depth.

(C) Dependence of the number of sites detected on the editing level cutoff.

(D) Boxplot showing the range of editing rates observed at each developmental stage. The box depicts the first to last quartiles, whiskers indicate 1.5 times the interquartile range, the center line represents the median, and points represent the outliers.

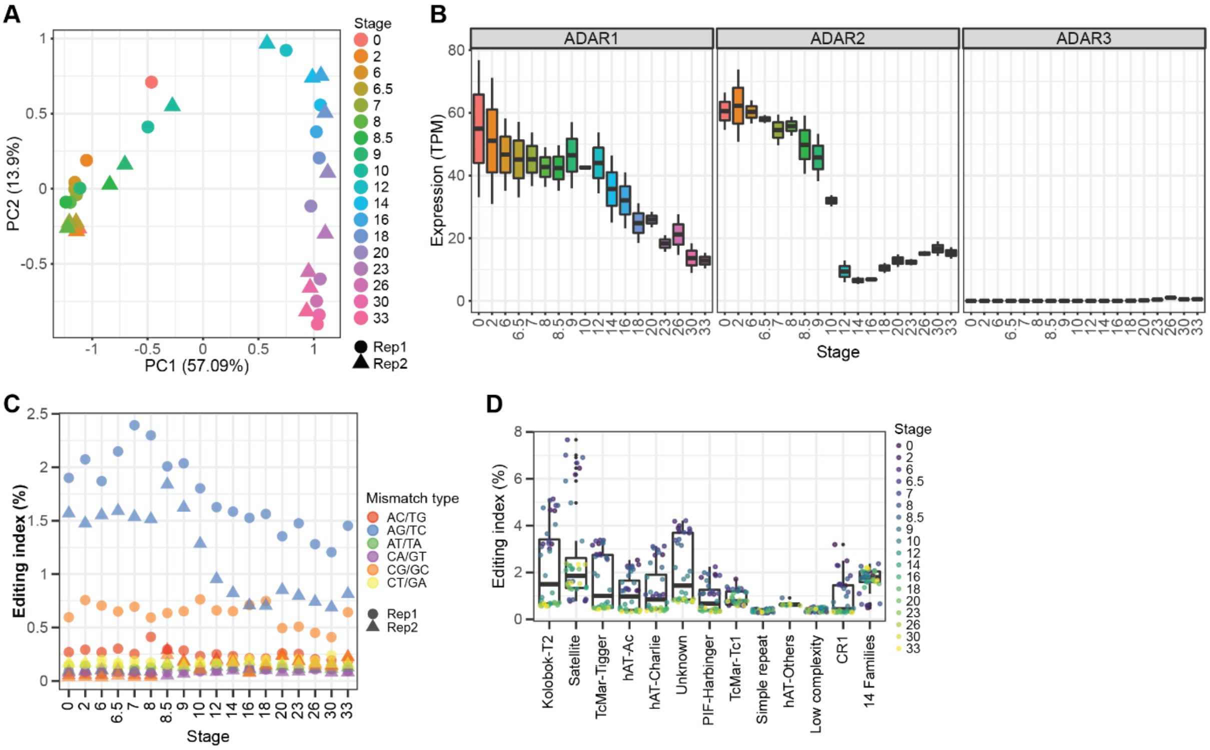

Supp. Fig. S10 ADAR expression and activity in embryogenesis data from the MK study.

(A) PCA plot based on gene expression values showing segregation of embryonic samples according to developmental stages.

(B) Transcript levels of ADAR enzymes across development in the MK study. The ADAR expression values are provided in Additional File 2.

(C) Global editing index measured across all repeat families in the MK study.

(D) Editing index for each individual repeat family in the MK study. 14 annotated repeat families contained comparatively few editing events and thus were grouped together for calculation of the index.

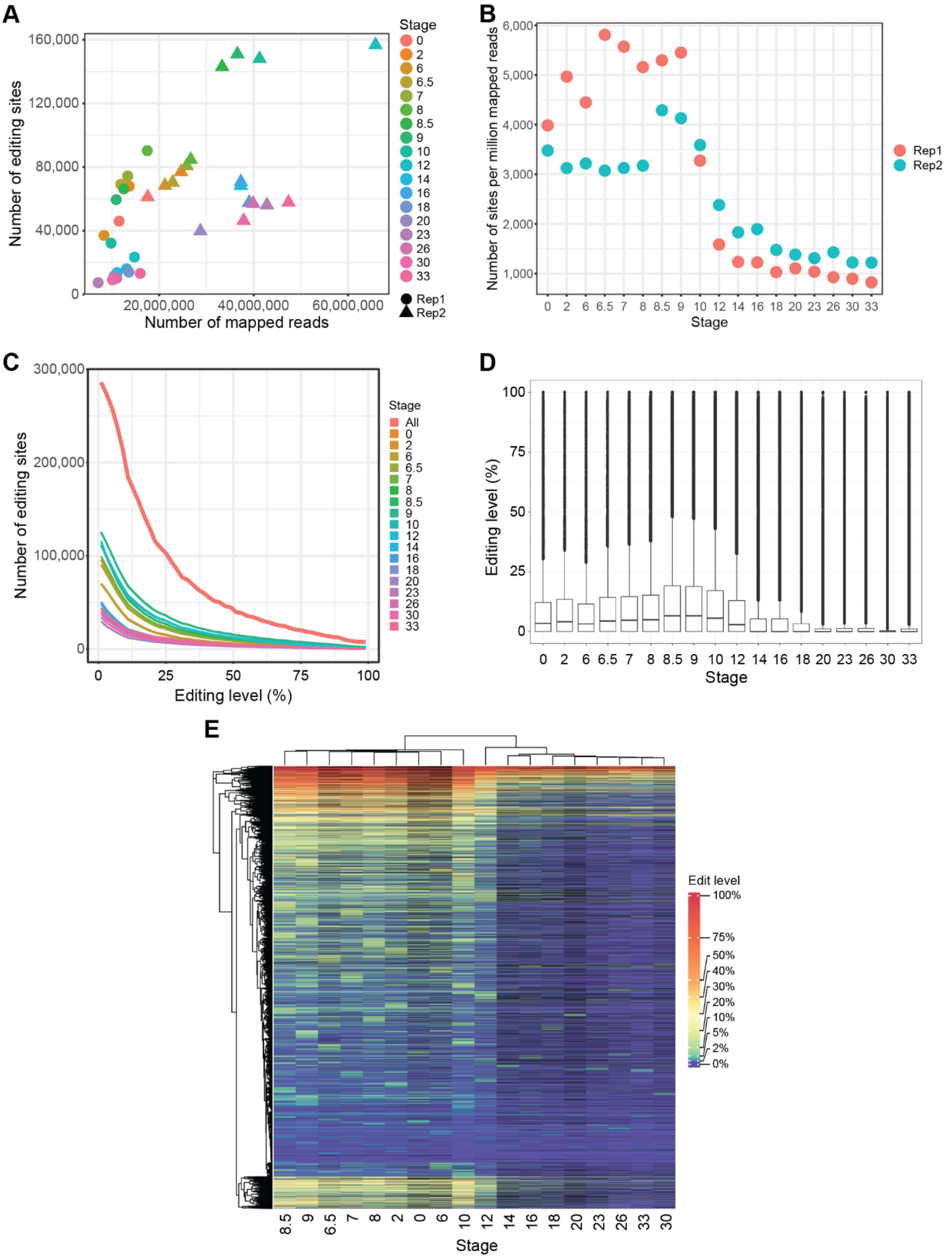
A
Number of editing sites
Number of mapped reads
Stage
0
2
6
6.5
7
8
8.5
9
10
12
14
16
18
20
23
26
30
33
Rep1
Rep2
B
Number of sites per million mapped reads
Stage
Rep1
Rep2
C
Number of editing sites
Editing level (%)
Stage
All
D
Editing level (%)
Stage
E
Edit level
100%
75%
50%
40%
30%
20%
10%
5%
2%
0%
Stage

Supp. Fig. S11 Individual A-to-I editing sites in embryogenesis data from the MK study.

(A) Scatterplot showing the number of editing events detected in and the sequencing depth of each dataset.

(B) Graph showing the number of editing events detected at each developmental stage normalized by the sequencing depth.

(C) Dependence of the number of sites detected on the editing level cutoff.

(D) Boxplot showing the range of editing rates observed at each developmental stage. The box depicts the first to last quartiles, whiskers indicate 1.5 times the interquartile range, the center line represents the median, and points represent the outliers.

(E) Hierarchical clustering of editing levels. Each row is a different editing site, while each column is a different developmental stage interrogated in the MK study.

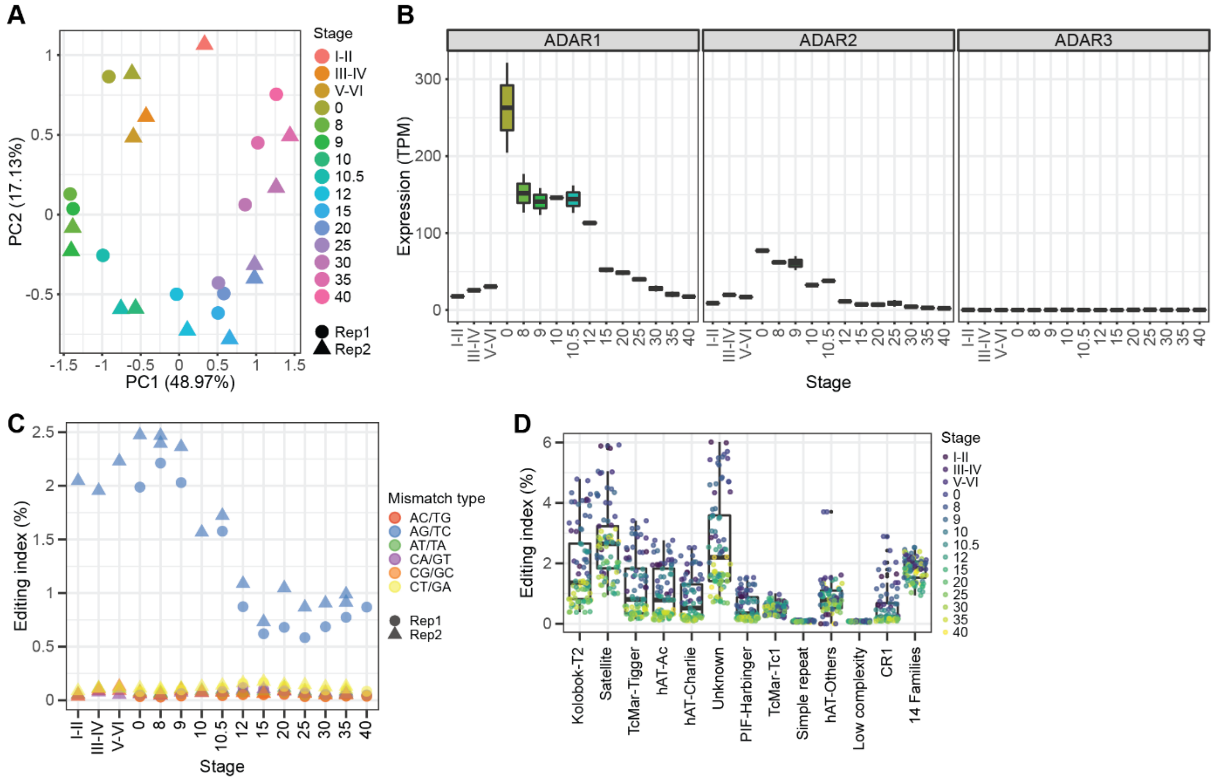

Supp. Fig. S12 ADAR expression and activity in embryogenesis data from the DR study.

(A) PCA plot based on gene expression values showing segregation of embryonic samples according to developmental stages.

(B) Transcript levels of ADAR enzymes across development in the DR study. The ADAR expression values are provided in Additional File 2.

(C) Global editing index measured across all repeat families in the DR study.

(D) Editing index for each individual repeat family in the DR study. 14 annotated repeat families contained comparatively few editing events and thus were grouped together for calculation of the index.

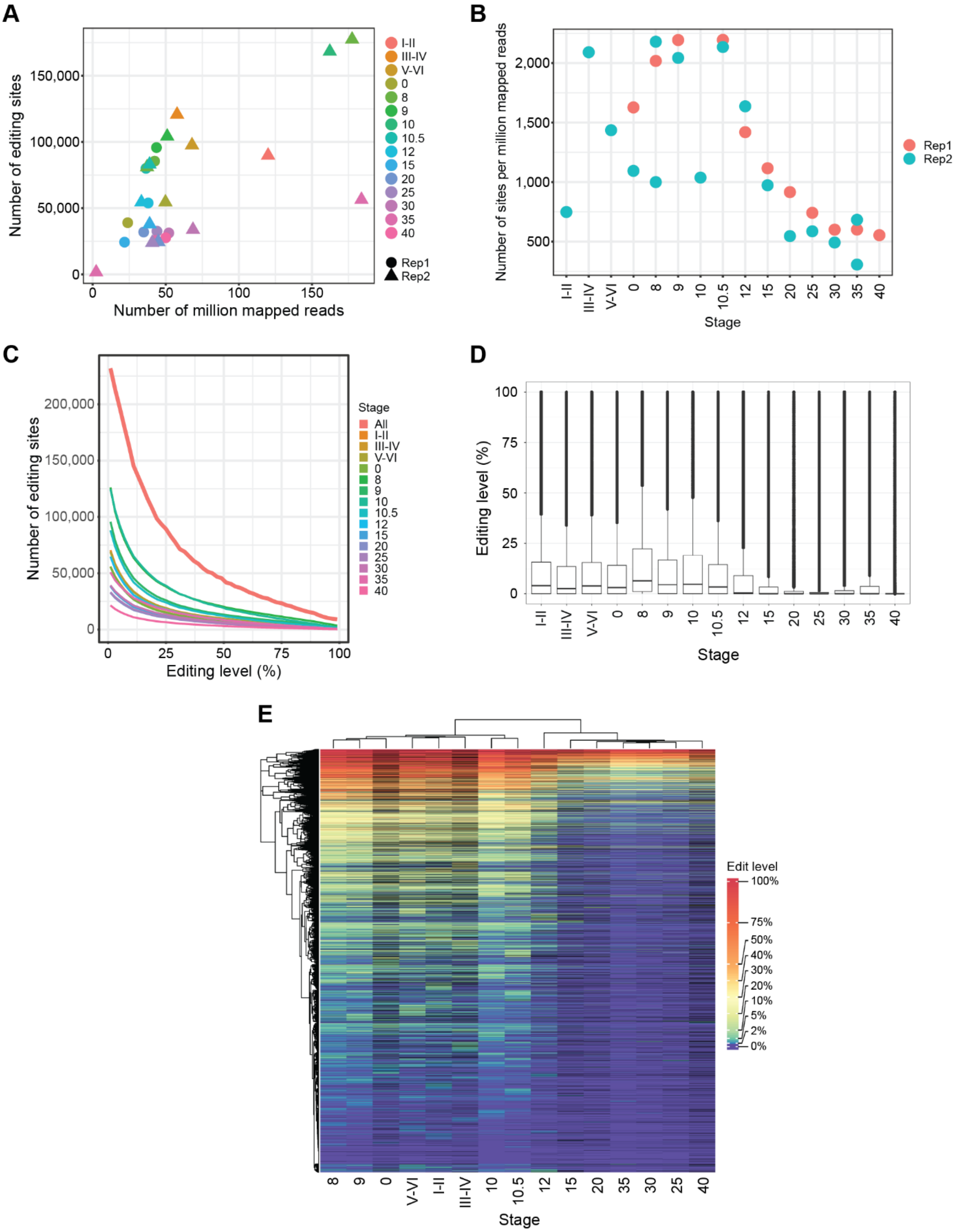
A
Number of editing sites
150,000
100,000
50,000
0
0
50
100
150
Number of million mapped reads
I-II
III-IV
V-VI
0
8
9
10
10.5
12
15
20
25
30
35
40
Rep1
Rep2
B
Number of sites per million mapped reads
2,000
1,500
1,000
500
Rep1
Rep2
Stage
C
Number of editing sites
200,000
150,000
100,000
50,000
0
0
25
50
75
100
Editing level (%)
Stage
All
D
Editing level (%)
100
75
50
25
0
Stage
E
Edit level
100%
75%
50%
40%
30%
20%
10%
5%
2%
0%
8
9
0
V-VI
I-II
III-IV
10
10.5
12
15
20
35
30
25
40
Stage

Supp. Fig. S13 Individual A-to-I editing sites in embryogenesis data from the DR study.

(A) Scatterplot showing the number of editing events detected in and the sequencing depth of each dataset.

(B) Graph showing the number of editing events detected at each developmental stage normalized by the sequencing depth.

(C) Dependence of the number of sites detected on the editing level cutoff.

(D) Boxplot showing the range of editing rates observed at each developmental stage. The box depicts the first to last quartiles, whiskers indicate 1.5 times the interquartile range, the center line represents the median, and points represent the outliers.

(E) Hierarchical clustering of editing levels. Each row is a different editing site, while each column is a different developmental stage interrogated in the DR study.

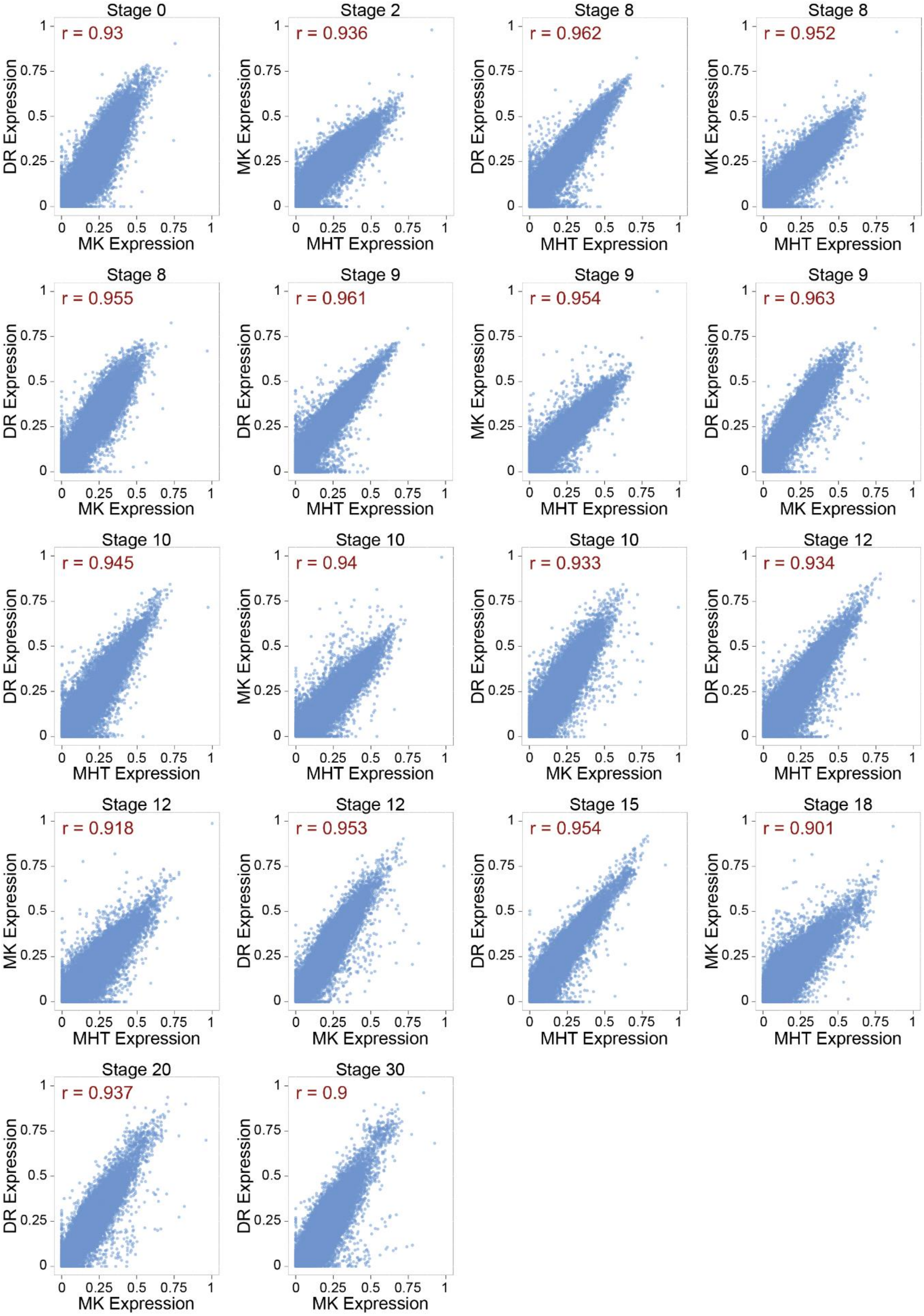

Supp. Fig. S14 Comparison of gene expression levels in *X. laevis* across studies.

High Pearson correlation coefficients of at least 0.9 were observed across all pairwise comparisons at matched developmental stages.

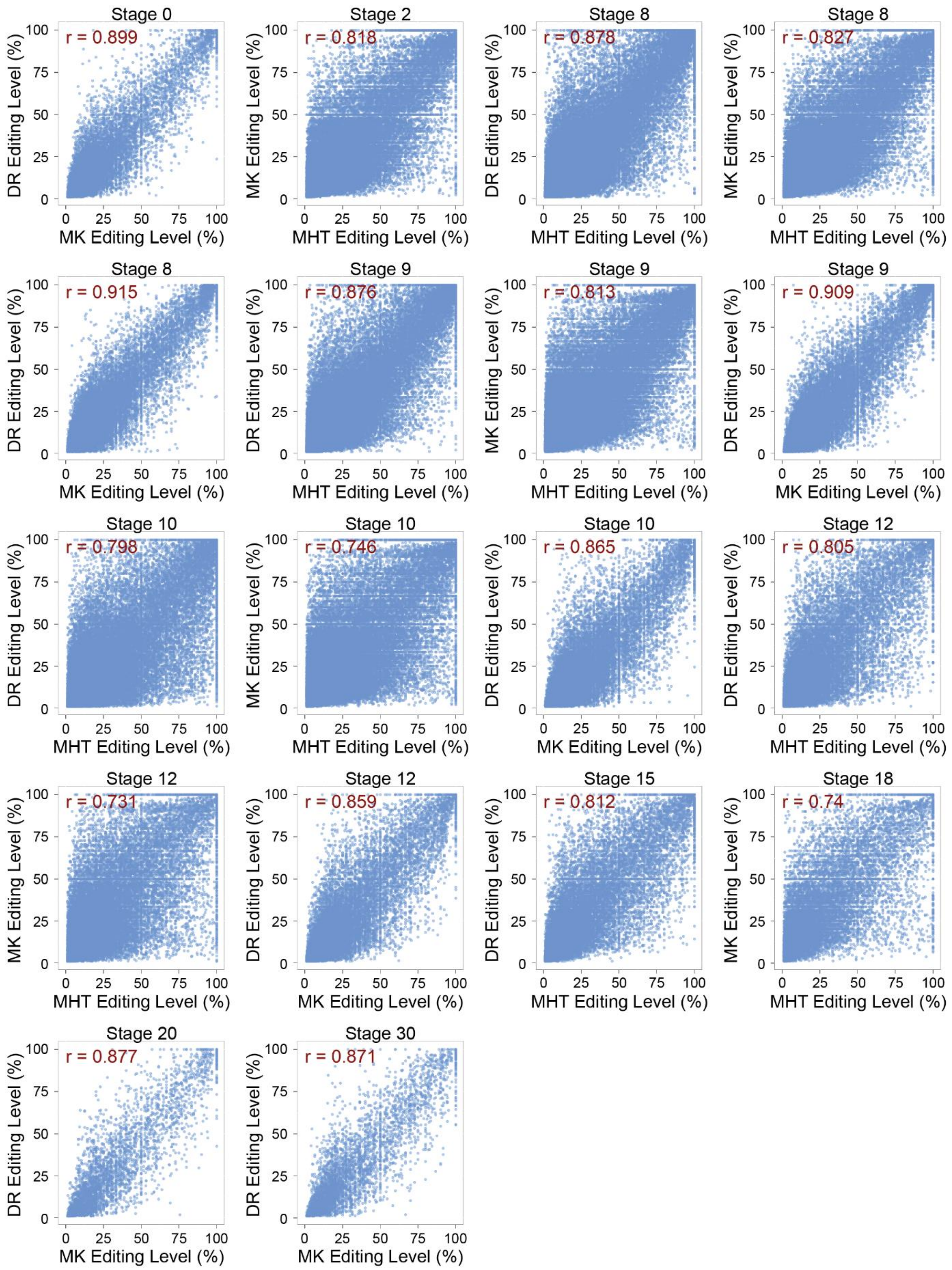

Supp. Fig. S15 Comparison of editing levels in *X. laevis* across studies.

Moderately high Pearson correlation coefficients of at least 0.7 were observed across all pairwise comparisons at matched developmental stages.

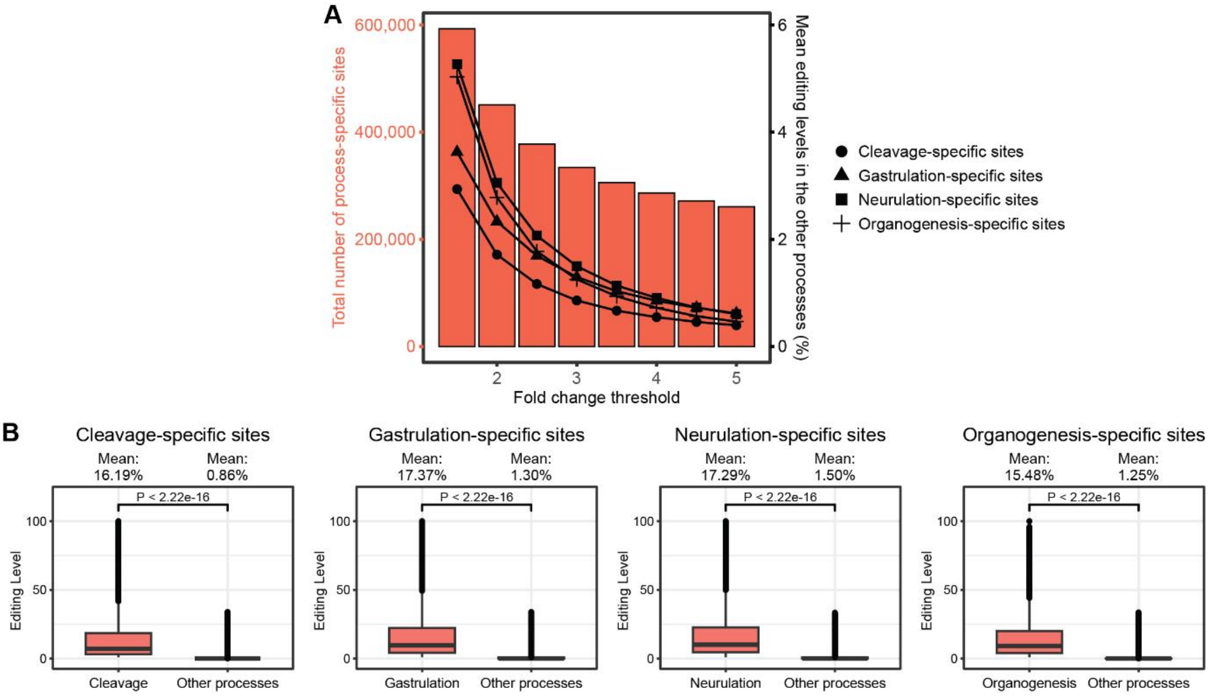

Supp. Fig. S16 Evaluation of different fold changes.

(A) To identify context-specific editing sites, we tested various thresholds for the fold change in editing between the developmental process-of-interest and the other processes. Expectedly, the total number of context-specific sites drops as the threshold becomes more stringent. The mean editing level of these sites in the other non-targeted processes also becomes lower.

(B) Boxplots showing the deamination rates of different groups of context-specific editing sites. The P-values were calculated using unpaired Wilcoxon rank sum test.

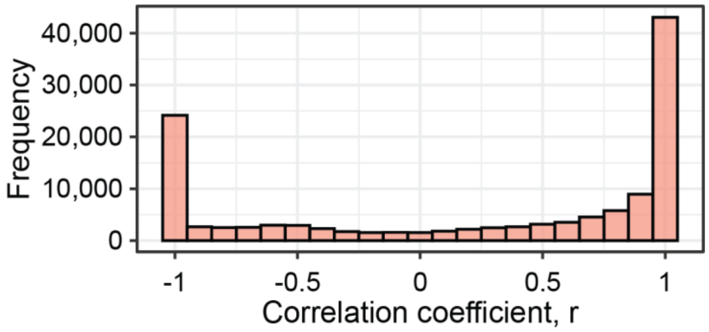

Supp. Fig. S17 Correlation between editing levels of developmental process-specific sites and expression levels of the host genes.

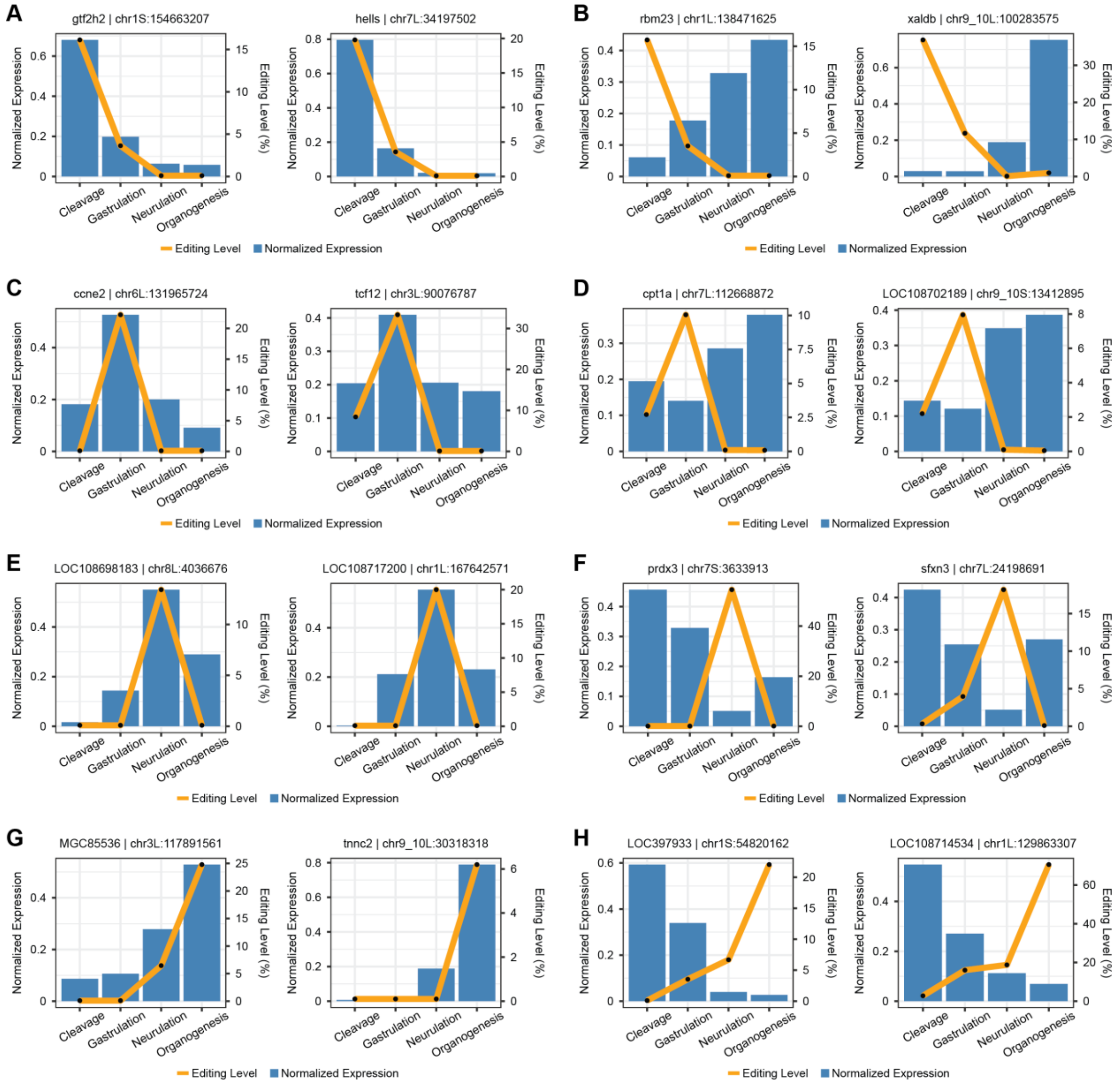

Supp. Fig. S18 Examples illustrating positive and negative relationships between A-to-I editing and gene expression.

(A) Genes whose editing of cleavage-specific sites correlated positively with expression.

(B) Genes whose editing of cleavage-specific sites correlated negatively with expression.

(C) Genes whose editing of gastrulation-specific sites correlated positively with expression.

(D) Genes whose editing of gastrulation-specific sites correlated negatively with expression.

(E) Genes whose editing of neurulation-specific sites correlated positively with expression.

(F) Genes whose editing of neurulation-specific sites correlated negatively with expression.

(G) Genes whose editing of organogenesis-specific sites correlated positively with expression.

(H) Genes whose editing of organogenesis-specific sites correlated negatively with expression.

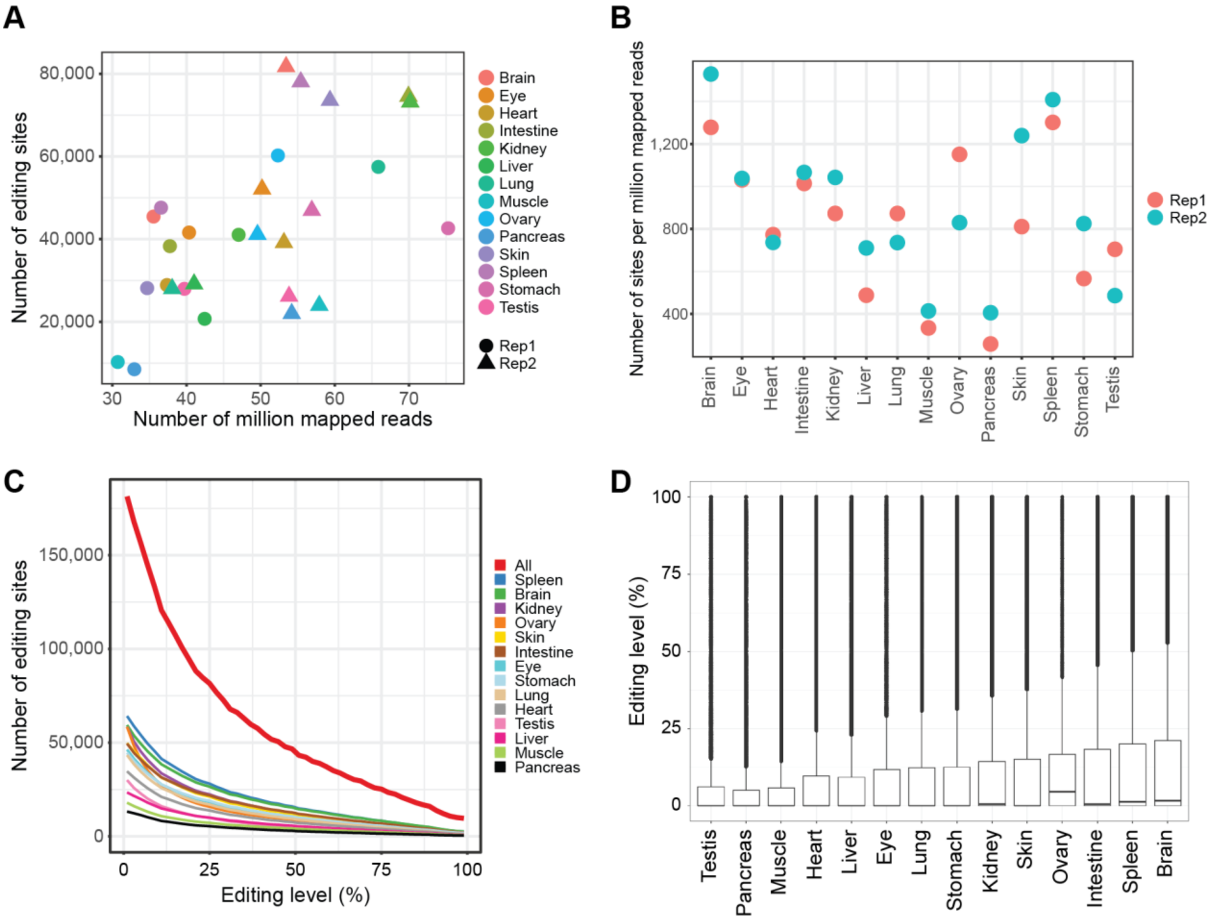

Supp. Fig. S19 Individual A-to-I editing sites in tissue data from the DR study.

(A) Scatterplot showing the number of editing events detected in and the sequencing depth of each dataset.

(B) Graph showing the number of editing events detected in each adult tissue normalized by the sequencing depth.

(C) Dependence of the number of sites detected on the editing level cutoff.

(D) Boxplot showing the range of editing rates observed in each adult tissue. The box depicts the first to last quartiles, whiskers indicate 1.5 times the interquartile range, the center line represents the median, and points represent the outliers.

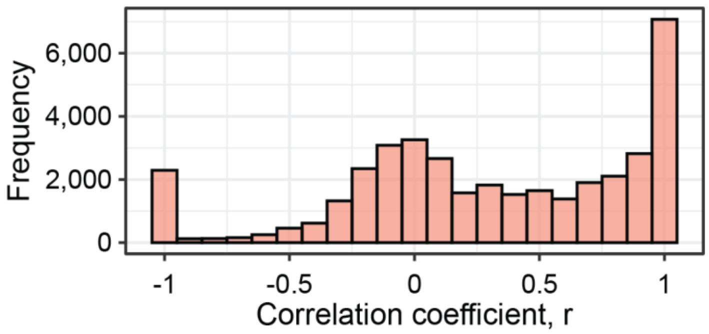

Supp. Fig. S20 Correlation between editing levels of tissue-specific sites and expression levels of the host genes.

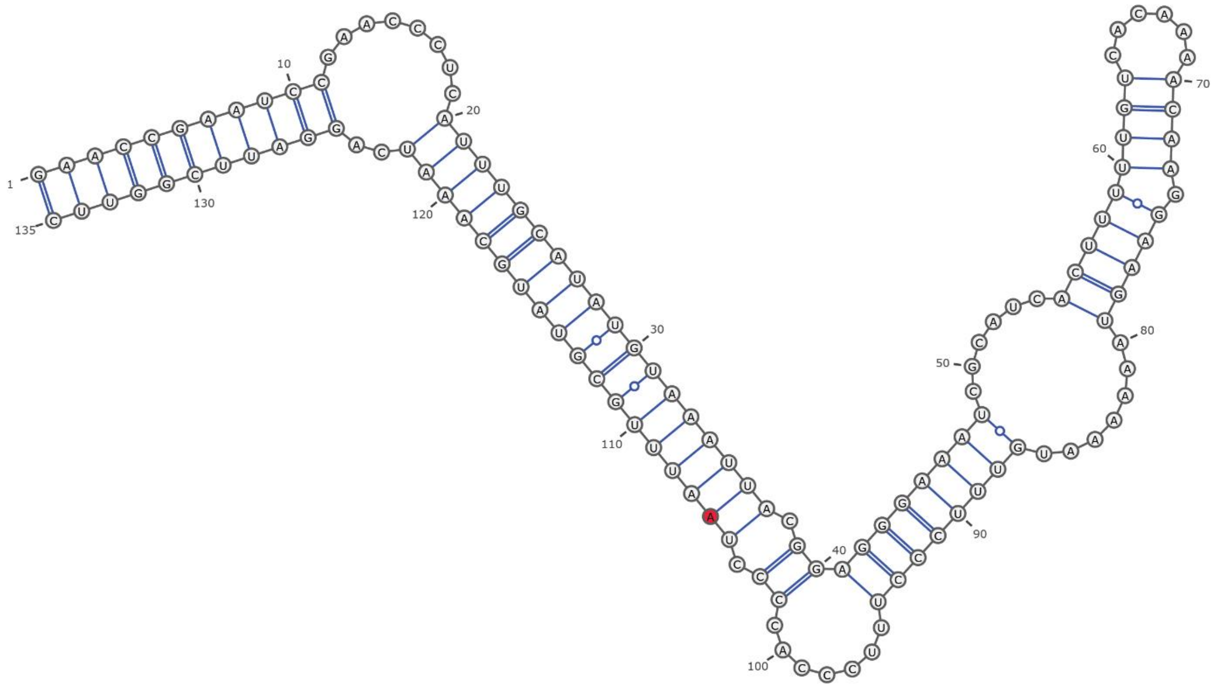

Supp. Fig. S21 Structure of the 3’UTR of cdc27.

RNAfold was used to predict the secondary structure of the primary transcript. The editing site, highlighted in red, is at chr9_10S:5524147 (xenLae2).

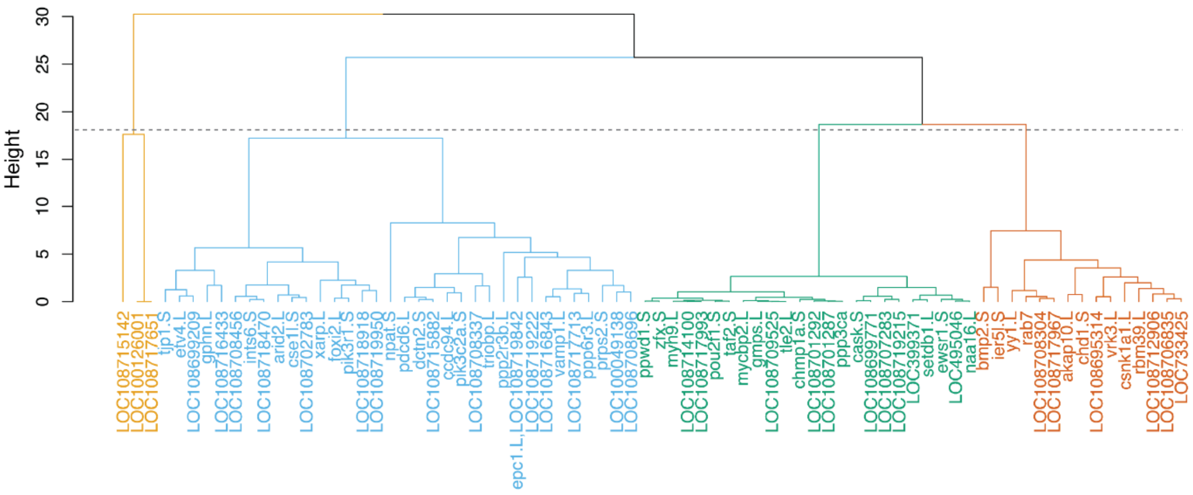

Supp. Fig. S22 Hierarchical clustering of genes containing two sites whose editing was significantly associated with each other (P < 0.01, χ^2-test).

No distance requirement was imposed between the two sites because we wanted to capture all potential *cis* effects. The dotted grey horizontal line separates the genes into four clusters, corresponding to Group 1 (3 genes in khaki), Group 2 (24 genes in green), Group 3 (15 genes in orange), and Group 4 (34 genes in blue) in **Fig. 4G**.

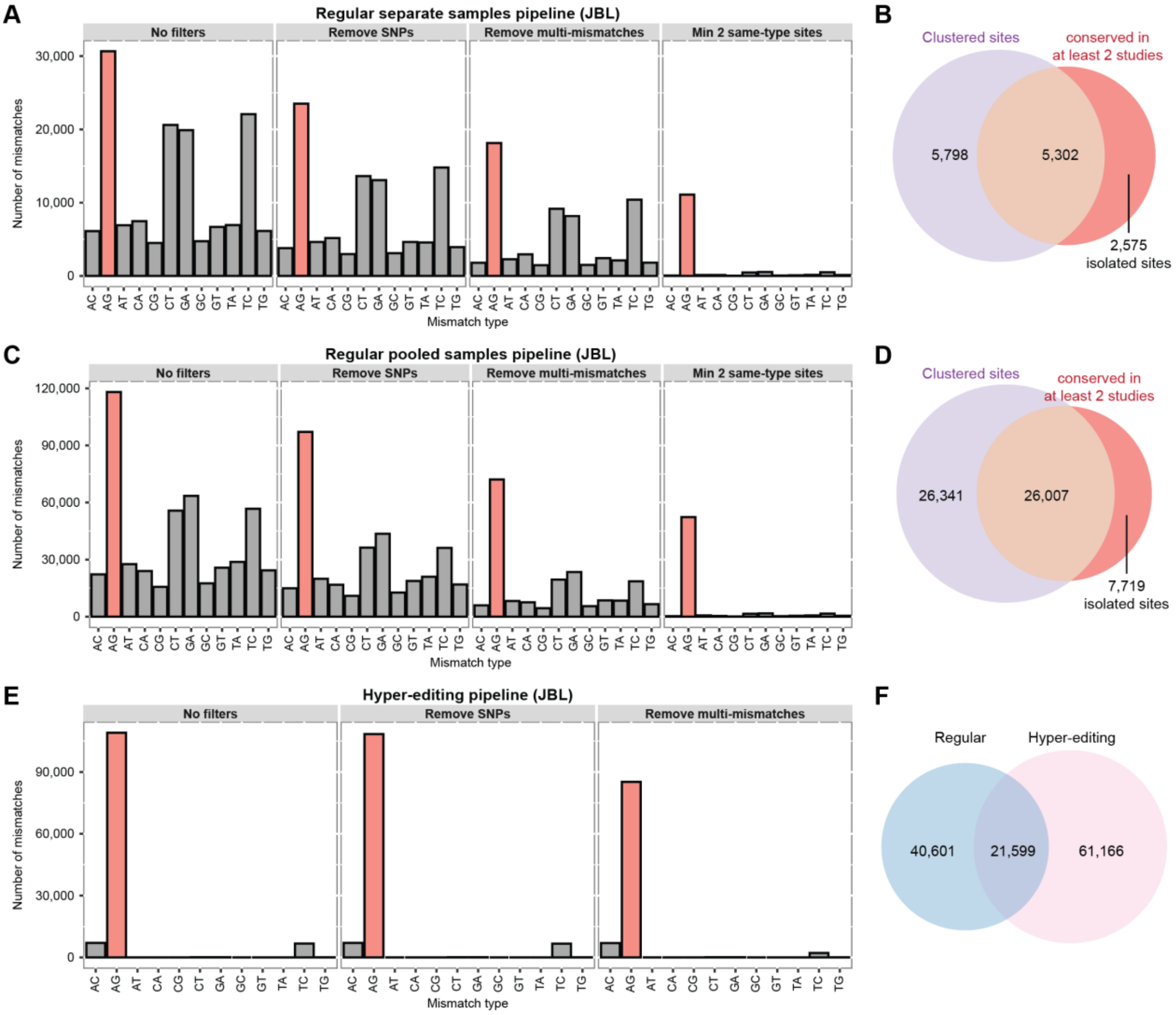

Supp. Fig. S23 Identification of A-to-I editing sites in *X. tropicalis* using RNA-seq data from the JBL study, where a total of 40 samples were analysed.

(A) Distribution of mismatch types at different steps of the separate samples analysis workflow. Each two-letter combination, XY, indicates X-to-Y mismatch.

(B) Venn diagram indicating the number of isolated editing sites recovered in the separate samples analysis workflow due to their detection in at least two different studies.

(C) Distribution of mismatch types at different steps of the pooled samples analysis workflow.

(D) Venn diagram indicating the number of isolated editing sites recovered in the pooled samples analysis workflow due to their detection in at least two different studies.

(E) Distribution of mismatch types at different steps of the hyper-editing analysis workflow.

(F) Venn diagram showing the number of editing sites found using regular read alignment and REDItools or the hyper-editing pipeline where mapping was done with all As converted to Gs.

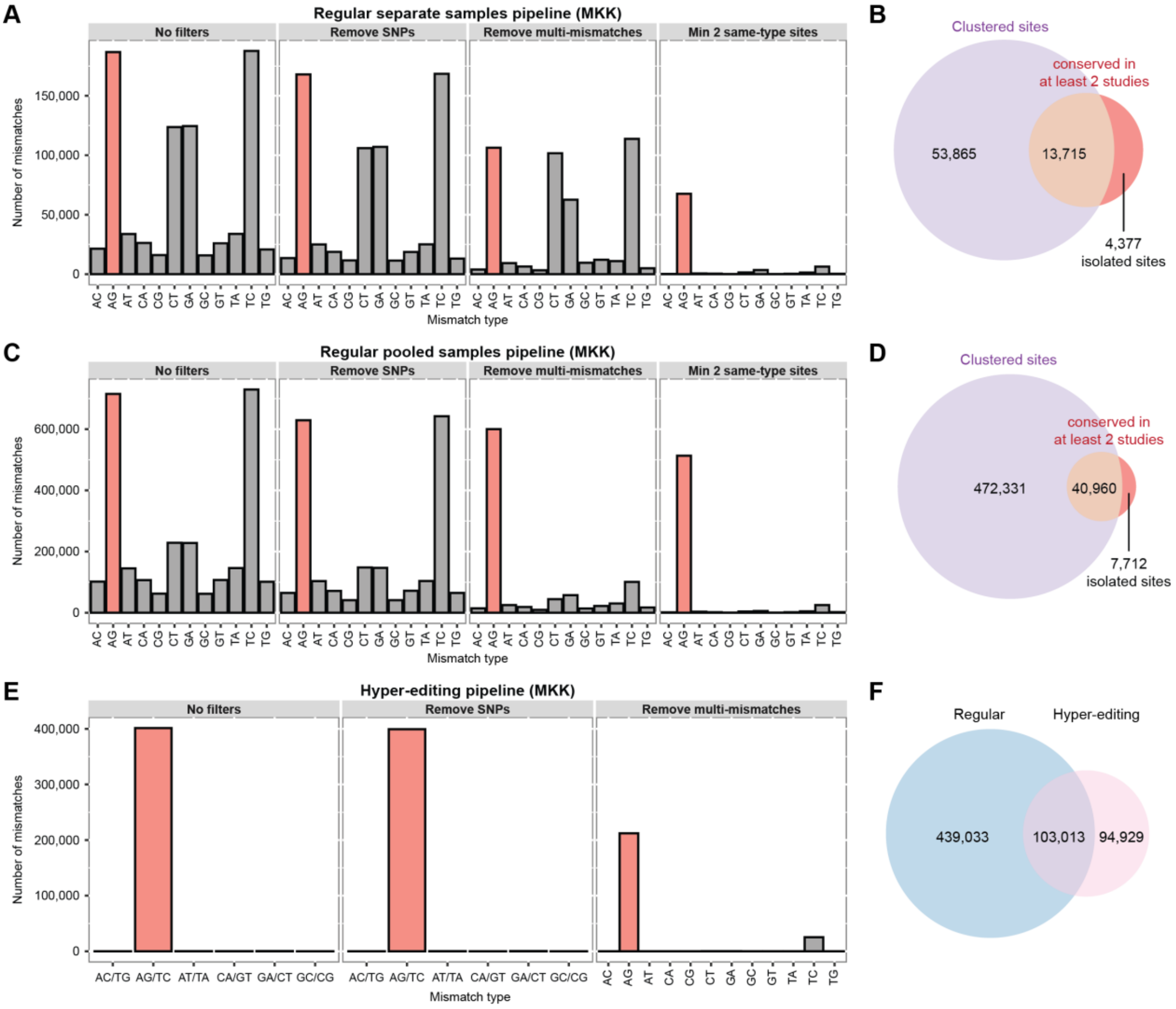

Supp. Fig. S24 Identification of A-to-I editing sites in *X. tropicalis* using RNA-seq data from the MKK study, where a total of 193 samples were analysed.

(A) Distribution of mismatch types at different steps of the separate samples analysis workflow. Each two-letter combination, XY, indicates X-to-Y mismatch.

(B) Venn diagram indicating the number of isolated editing sites recovered in the separate samples analysis workflow due to their detection in at least two different studies.

(C) Distribution of mismatch types at different steps of the pooled samples analysis workflow.

(D) Venn diagram indicating the number of isolated editing sites recovered in the pooled samples analysis workflow due to their detection in at least two different studies.

(E) Distribution of mismatch types at different steps of the hyper-editing analysis workflow.

(F) Venn diagram showing the number of editing sites found using regular read alignment and REDItools or the hyper-editing pipeline where mapping was done with all As converted to Gs.

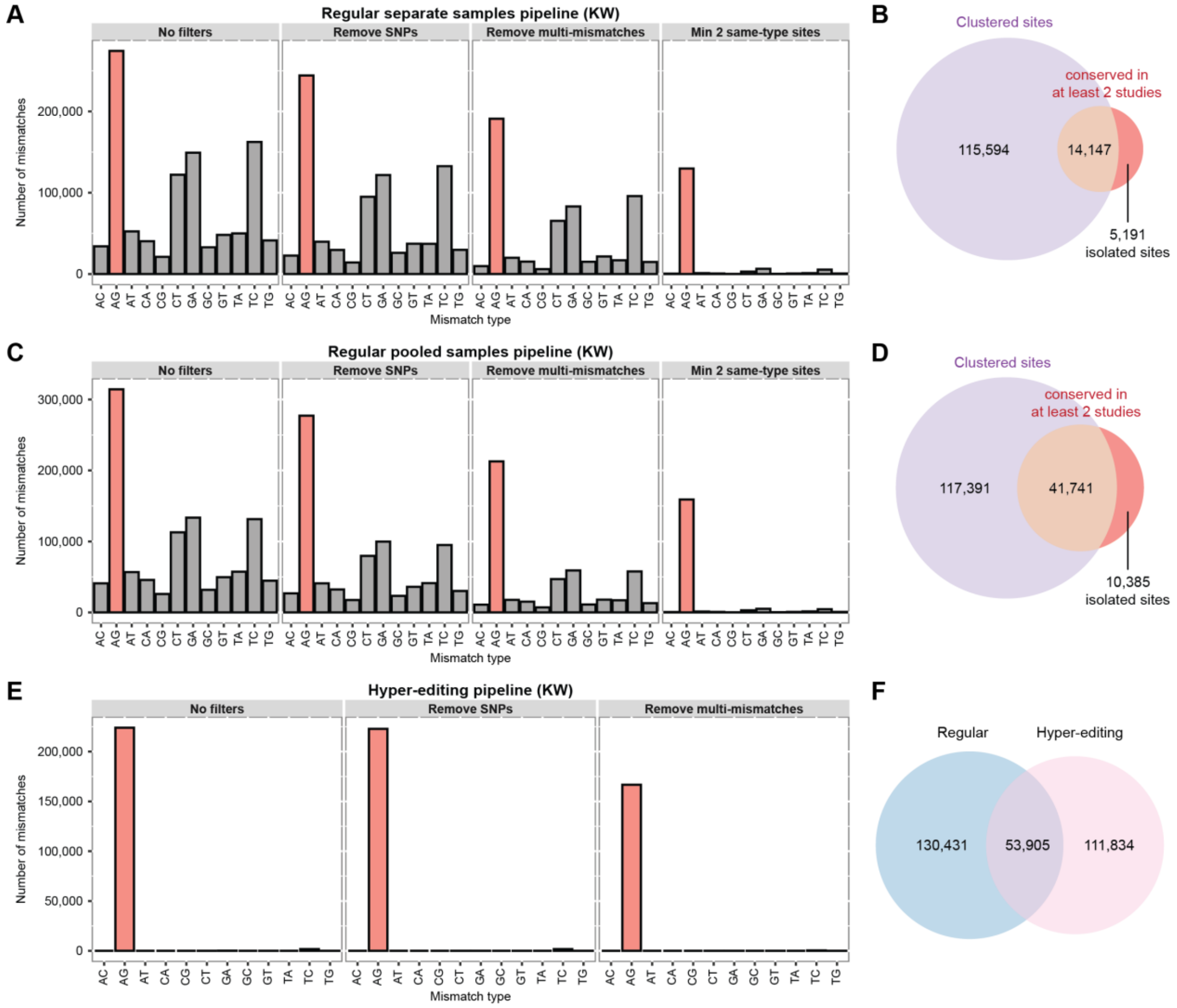

Supp. Fig. S25 Identification of A-to-I editing sites in *X. tropicalis* using RNA-seq data from the KW study, where a total of 6 samples were analysed.

(A) Distribution of mismatch types at different steps of the separate samples analysis workflow. Each two-letter combination, XY, indicates X-to-Y mismatch.

(B) Venn diagram indicating the number of isolated editing sites recovered in the separate samples analysis workflow due to their detection in at least two different studies.

(C) Distribution of mismatch types at different steps of the pooled samples analysis workflow.

(D) Venn diagram indicating the number of isolated editing sites recovered in the pooled samples analysis workflow due to their detection in at least two different studies.

(E) Distribution of mismatch types at different steps of the hyper-editing analysis workflow.

(F) Venn diagram showing the number of editing sites found using regular read alignment and REDItools or the hyper-editing pipeline where mapping was done with all As converted to Gs.

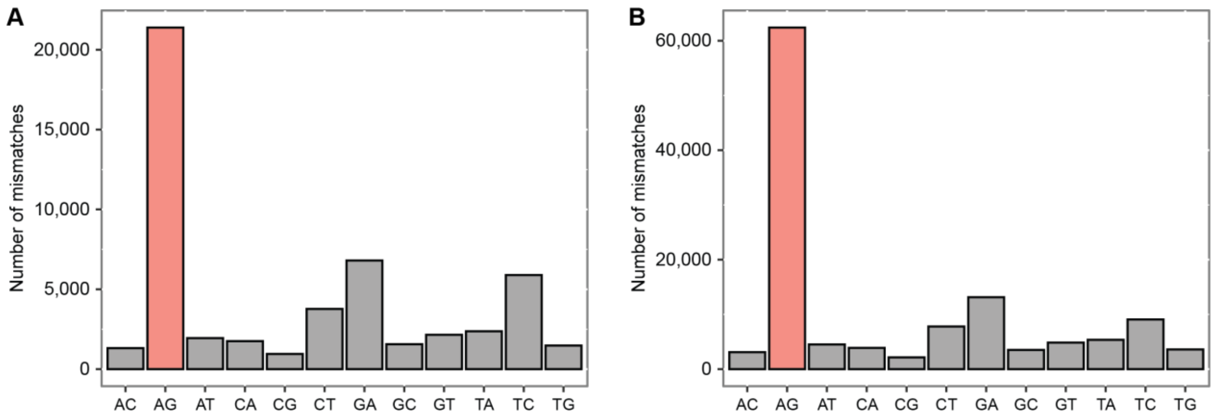

Supp. Fig. S26 Distribution of mismatch types for recovered isolated sites in *X. tropicalis* identified using our regular **(A)** separate samples or **(B)** pooled samples pipeline.

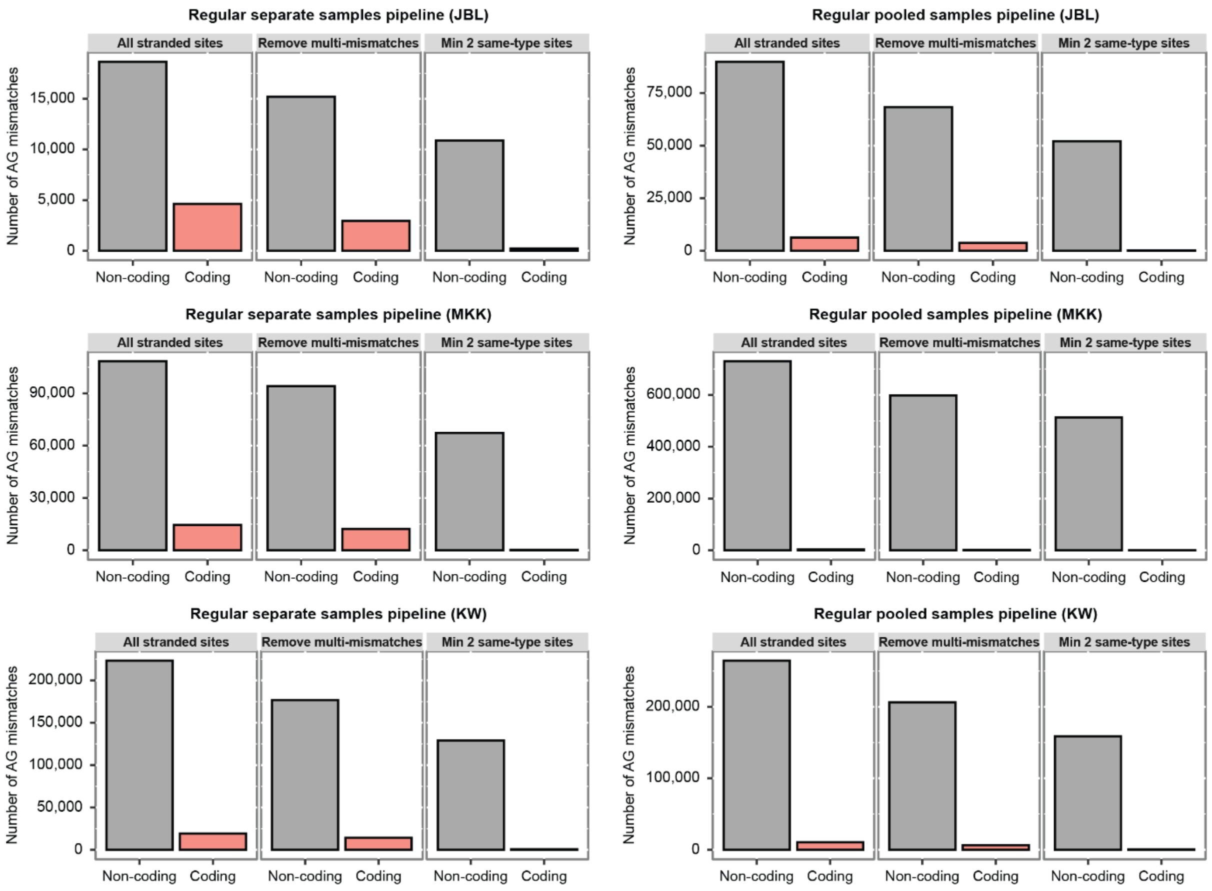

Supp. Fig. S27 Number of potential A-to-I editing sites in non-coding or coding regions after each step of filtering for all the *X. tropicalis* studies.

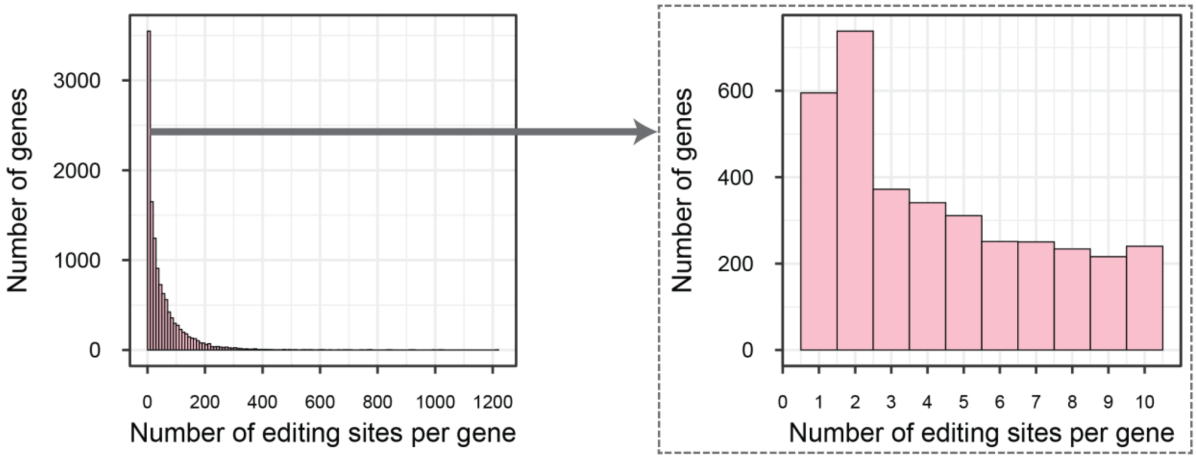

Supp. Fig. S28 Number of ADAR target sites per gene in *X. tropicalis*.

Histograms showing the distribution of editing site counts per gene in *X. tropicalis*. The left histogram has a bin size of 10, while the right histogram is a zoomed-in graph of the first bin.

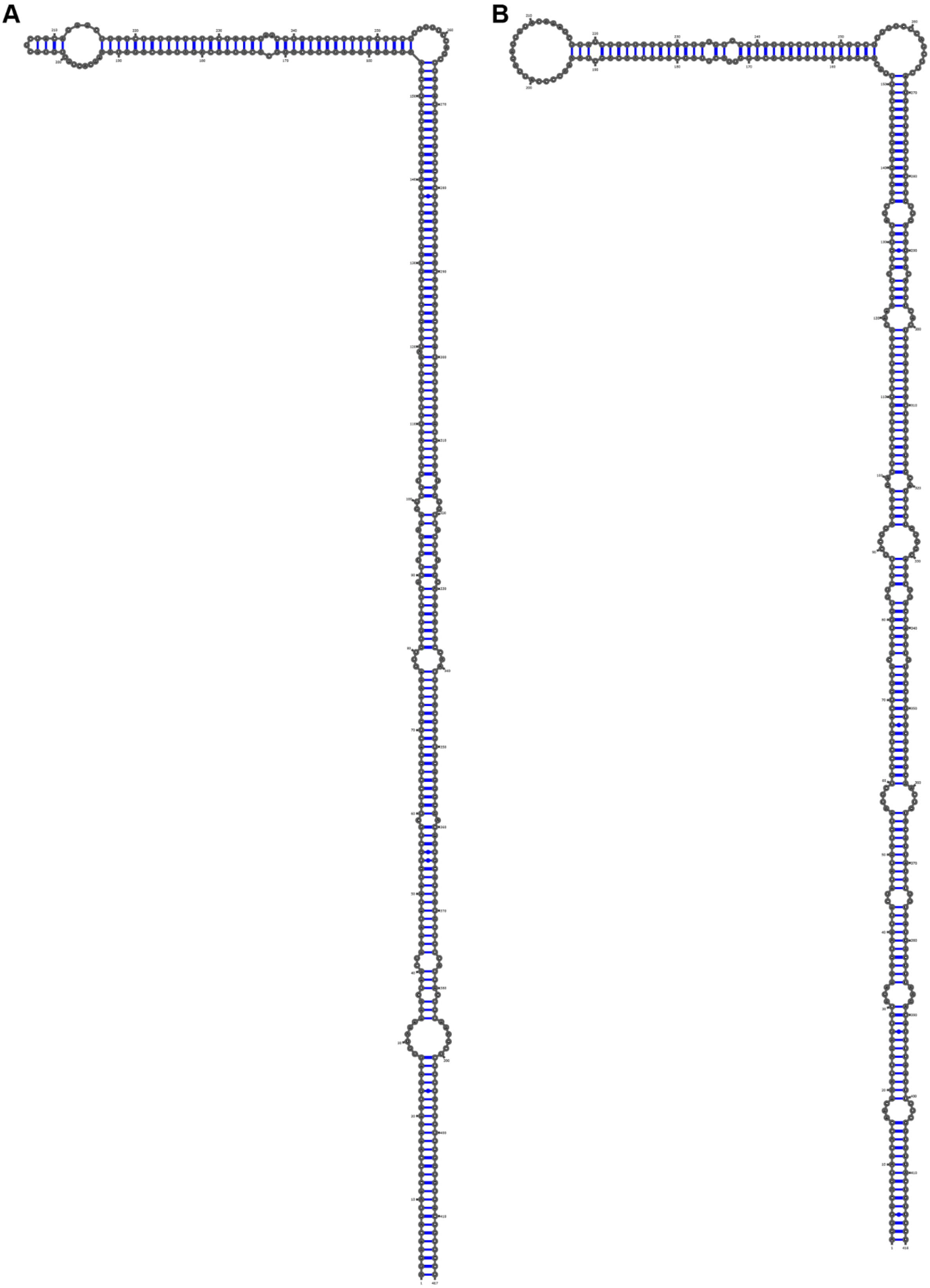

Supp. Fig. S29 Examples of dsRNA structures in repetitive regions of the *X. tropicalis* transcriptome.

RNAfold was used to predict the secondary structures of Kolobok elements in the **(A)** snrpf and **(B)** zak genes in *X. tropicalis*.

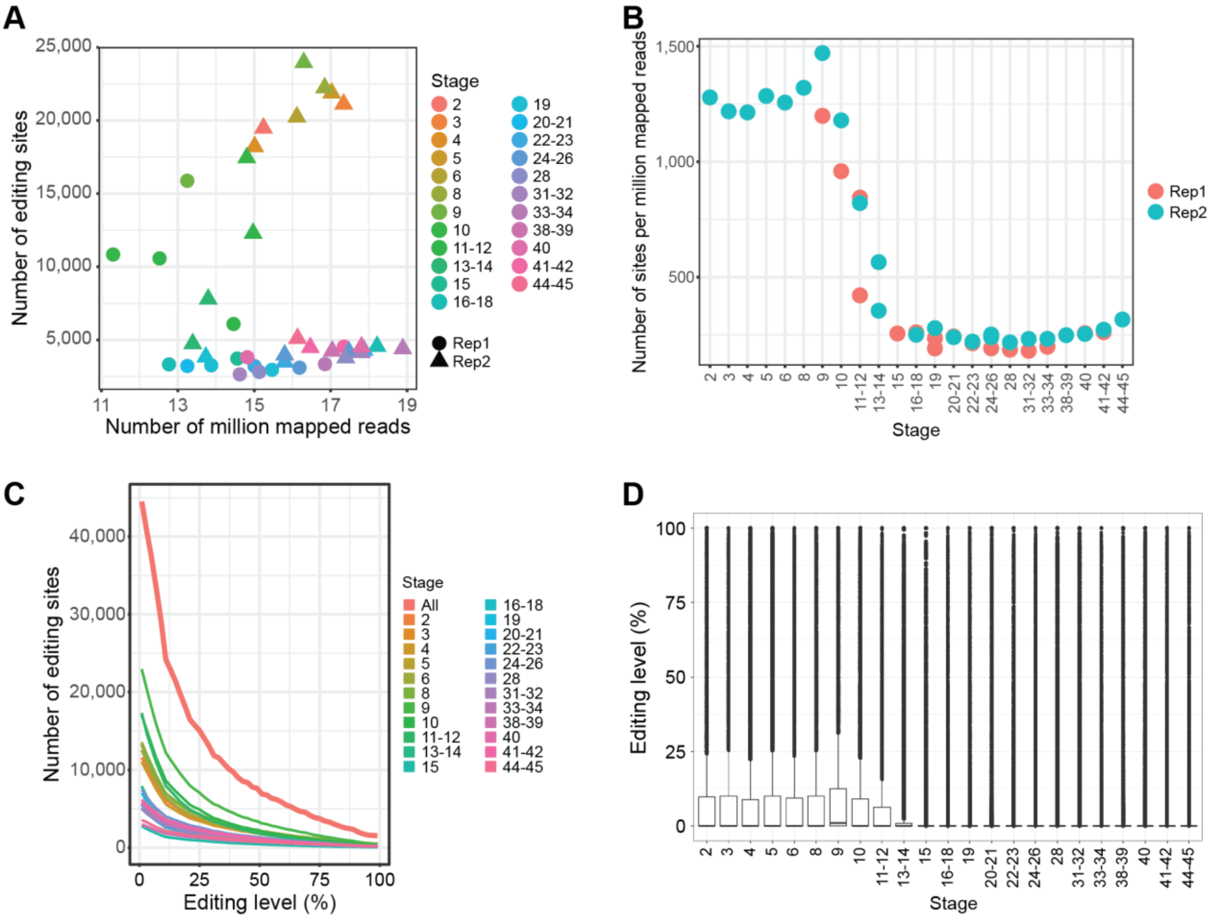

Supp. Fig. S30 Individual A-to-I editing sites in embryogenesis data from the JBL study.

(A) Scatterplot showing the number of editing events detected in and the sequencing depth of each dataset.

(B) Graph showing the number of editing events detected at each developmental stage normalized by the sequencing depth.

(C) Dependence of the number of sites detected on the editing level cutoff.

(D) Boxplot showing the range of editing rates observed at each developmental stage. The box depicts the first to last quartiles, whiskers indicate 1.5 times the interquartile range, the center line represents the median, and points represent the outliers.

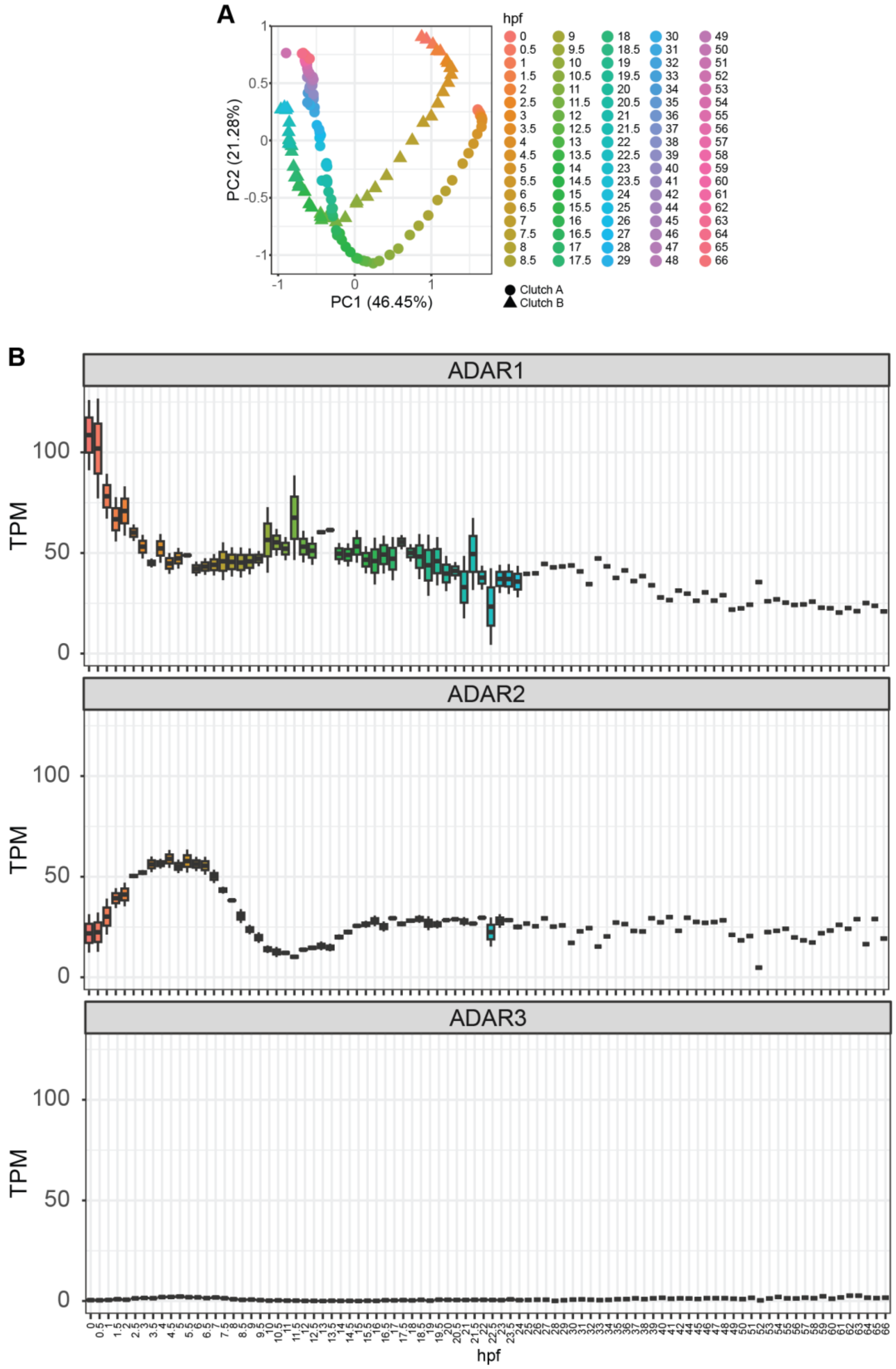

Supp. Fig. S31 Gene expression analysis of embryogenesis data from the MKK study.

(A) PCA plot based on gene expression values showing segregation of embryonic samples according to developmental stages.

(B) Transcript levels of ADAR enzymes across development in the MKK study. The ADAR expression values are provided in Additional File 2.

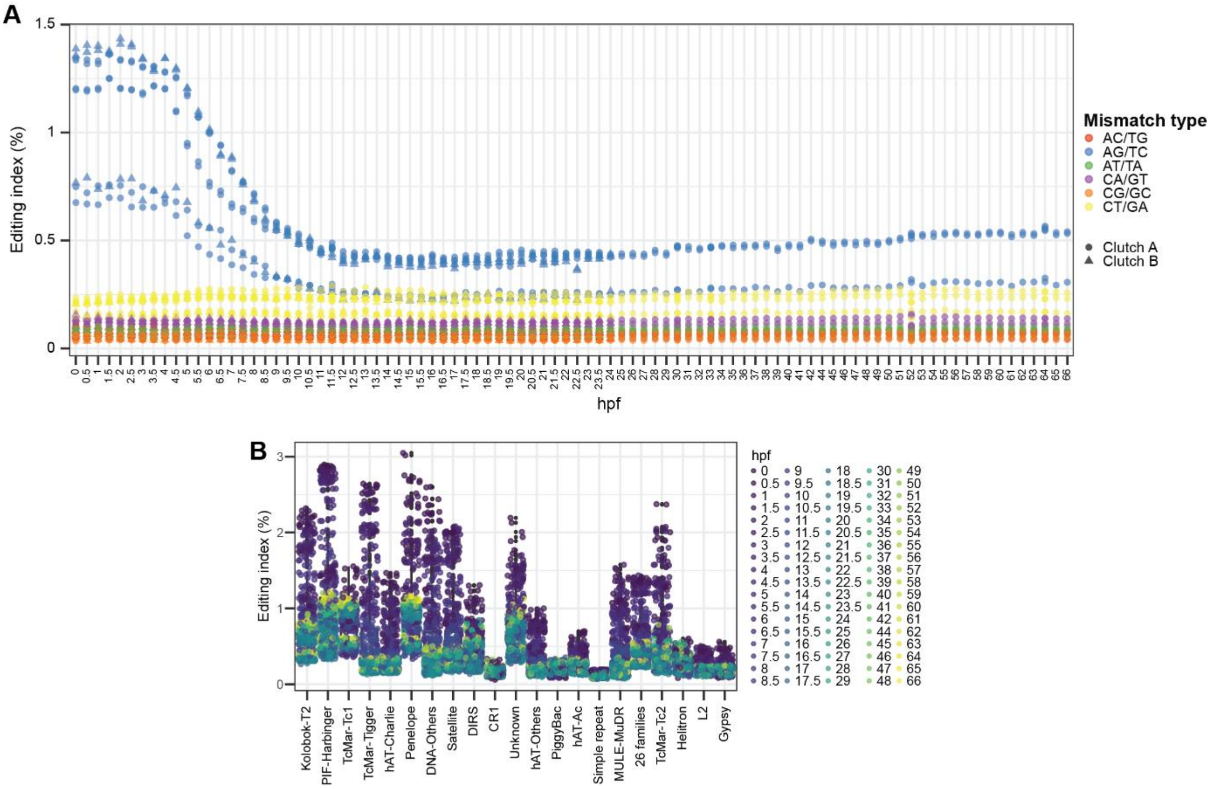

Supp. Fig. S32 ADAR activity in embryogenesis data from the MKK study.

(A) Global editing index measured across all repeat families in the MKK study.

(B) Editing index for each individual repeat family in the MKK study. 26 annotated repeat families contained comparatively few editing events and thus were grouped together for calculation of the index.

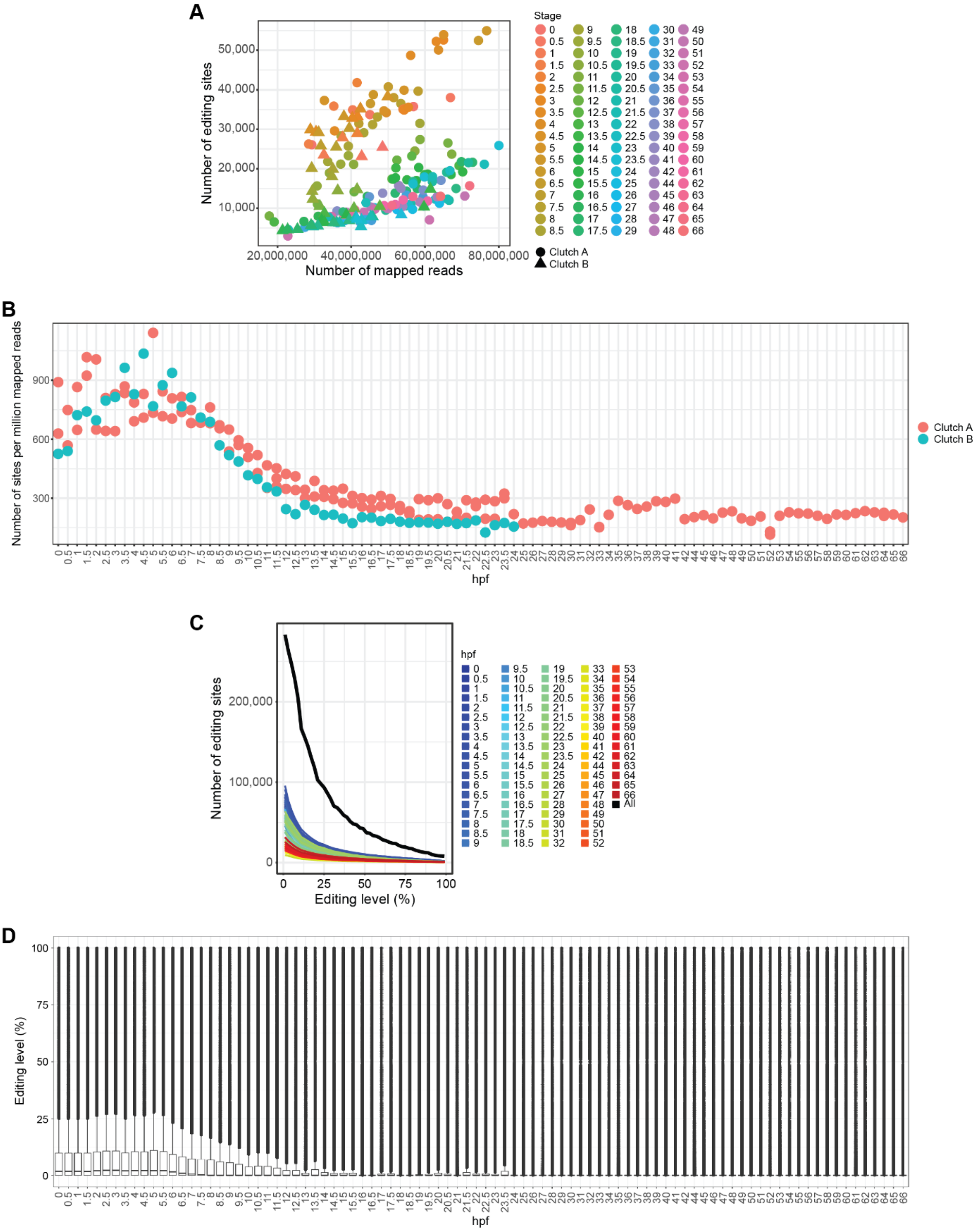
A
Number of editing sites
Number of mapped reads
Stage
Clutch A
Clutch B
B
Number of sites per million mapped reads
hpf
Clutch A
Clutch B
C
Number of editing sites
Editing level (%)
hpf
All
D
Editing level (%)
hpf

Supp. Fig. S33 Individual A-to-I editing sites in embryogenesis data from the MKK study.

(A) Scatterplot showing the number of editing events detected in and the sequencing depth of each dataset.

(B) Graph showing the number of editing events detected at each developmental stage normalized by the sequencing depth.

(C) Dependence of the number of sites detected on the editing level cutoff.

(D) Boxplot showing the range of editing rates observed at each developmental stage. The box depicts the first to last quartiles, whiskers indicate 1.5 times the interquartile range, the center line represents the median, and points represent the outliers.

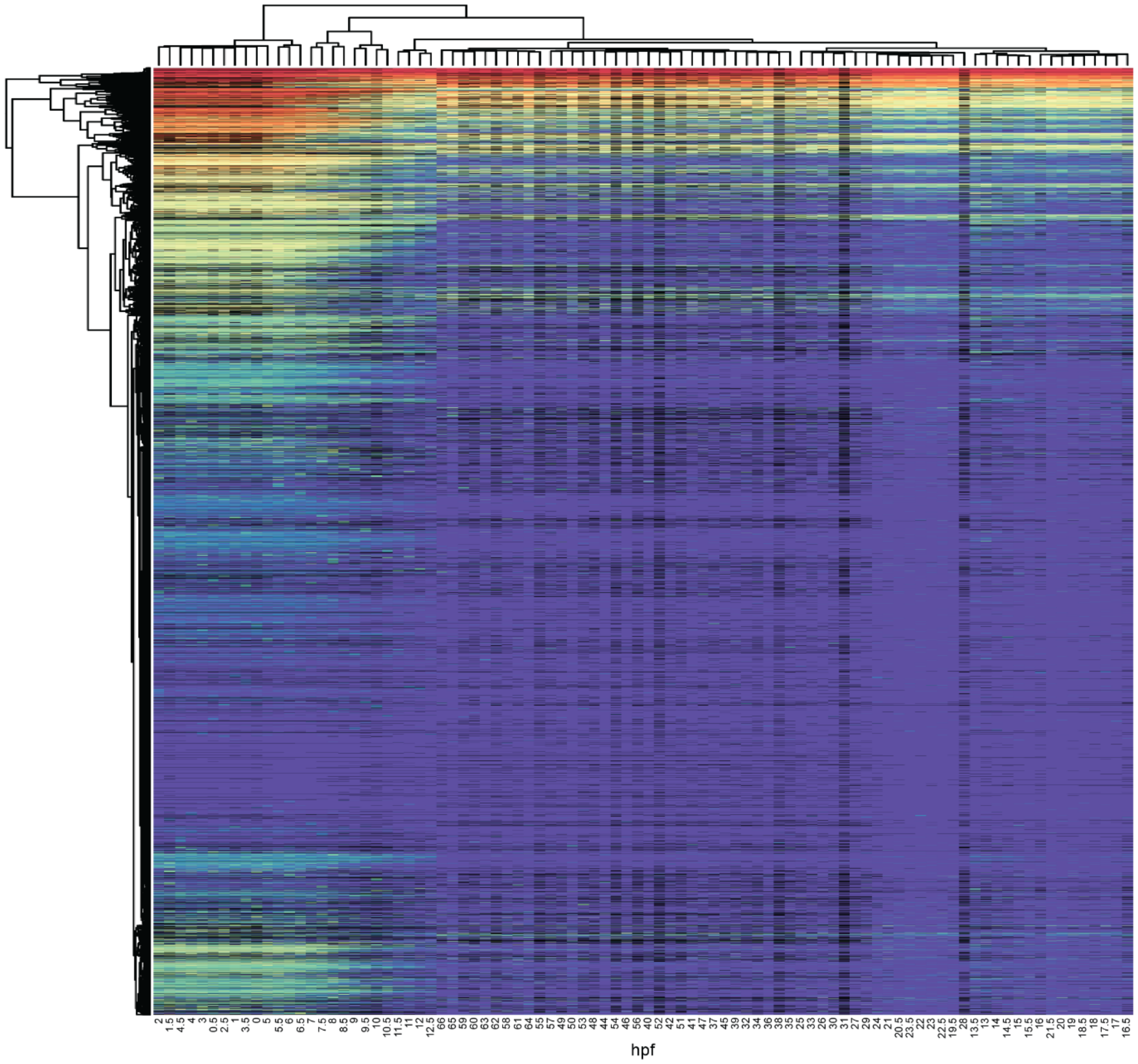

Supp. Fig. S34 Hierarchical clustering of editing levels from the MKK study.

Each row is a different editing site, while each column is a different developmental stage interrogated in the MKK study.

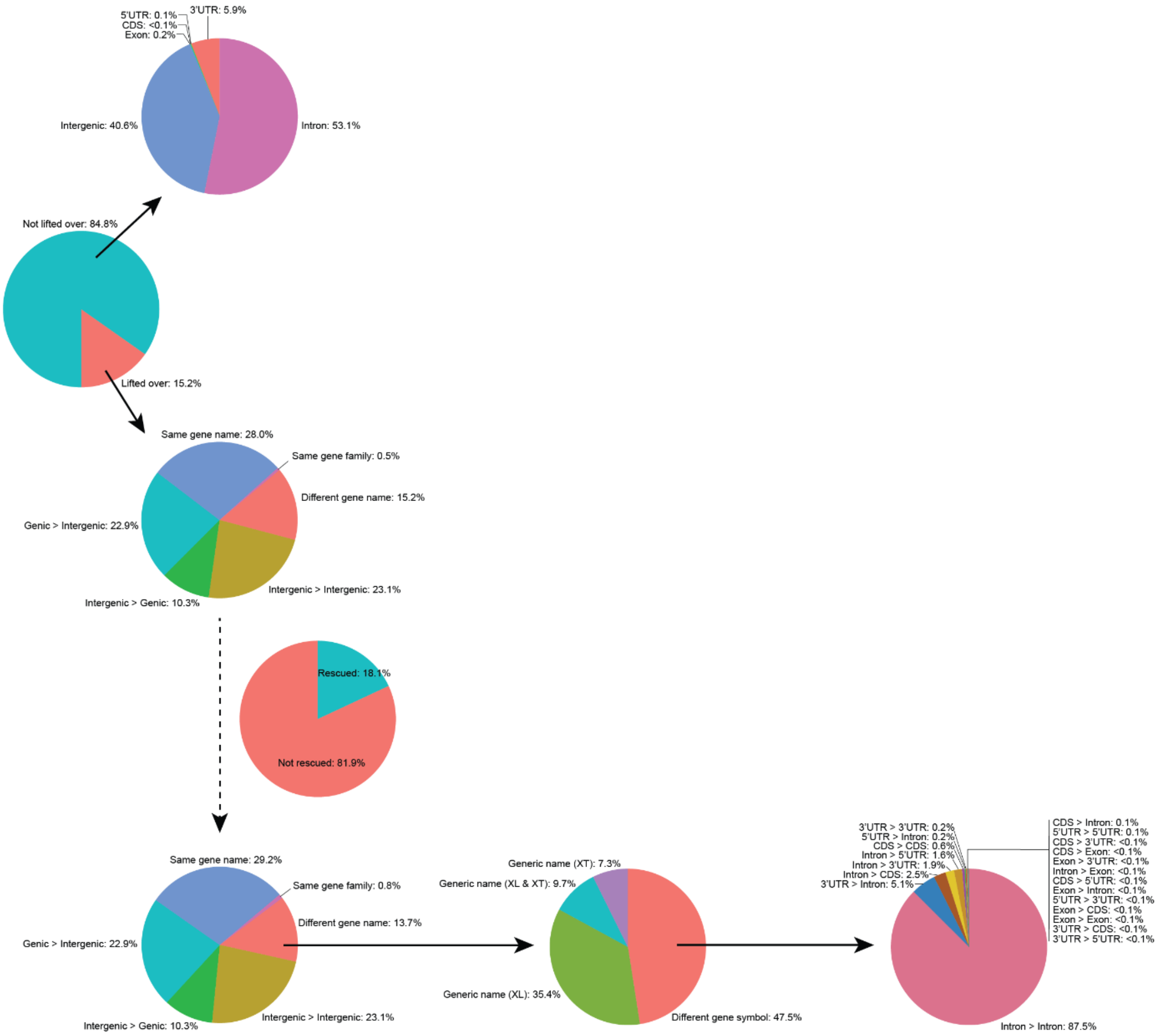

Supp. Fig. S35 Examination of *X. laevis* editing sites in *X. tropicalis*.

An overview of the analysis workflow. Majority of the sites could not be lifted over as many of them were in poorly conserved intronic or intergenic regions. In addition, under 10% of the matched sites were found in genes with conflicting symbols between the two frog species.

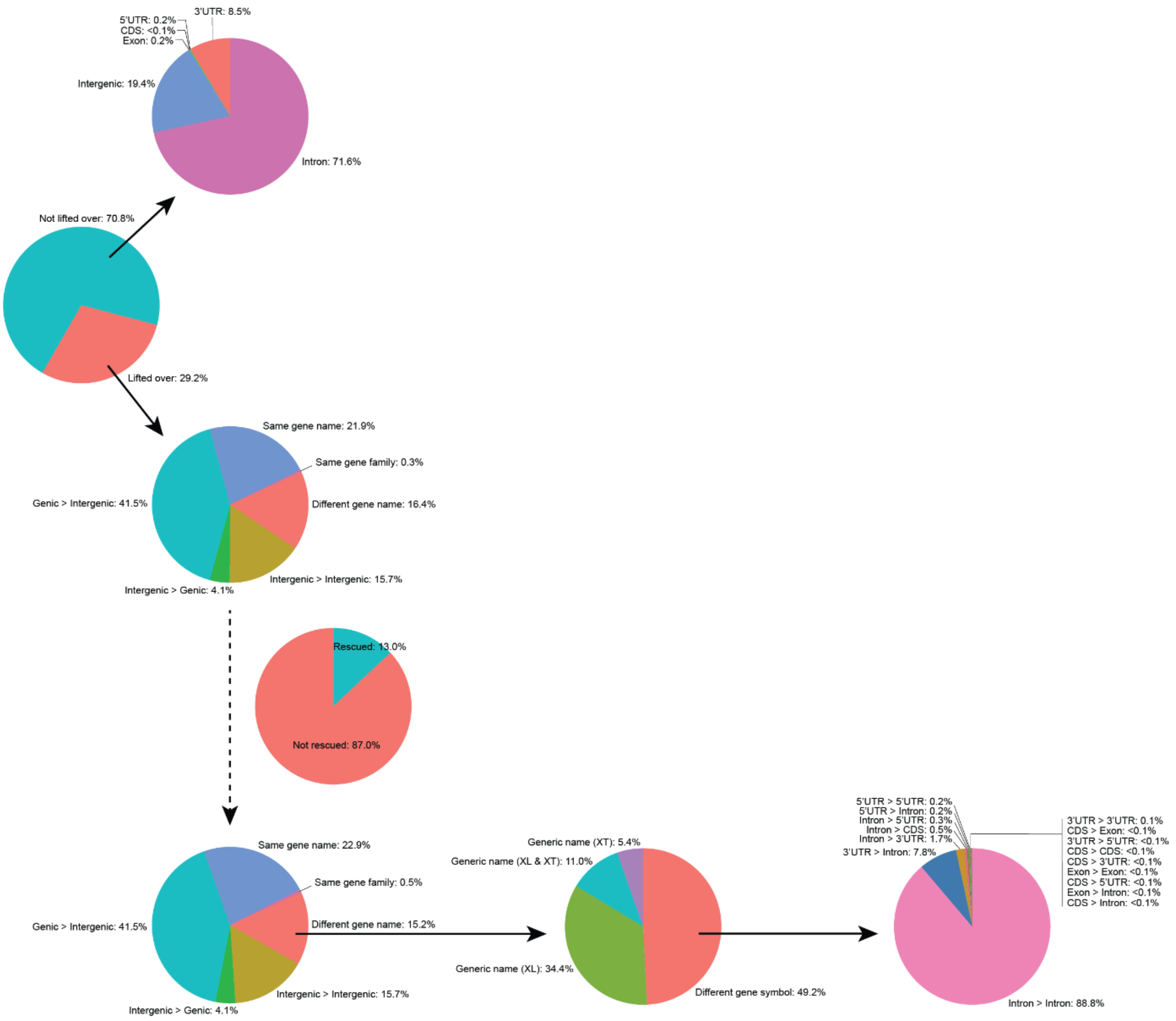

Supp. Fig. S36 Examination of *X. tropicalis* editing sites in *X. laevis*.

An overview of the analysis workflow. Majority of the sites could not be lifted over as many of them were in poorly conserved intronic or intergenic regions. In addition, under 10% of the matched sites were found in genes with conflicting symbols between the two frog species.

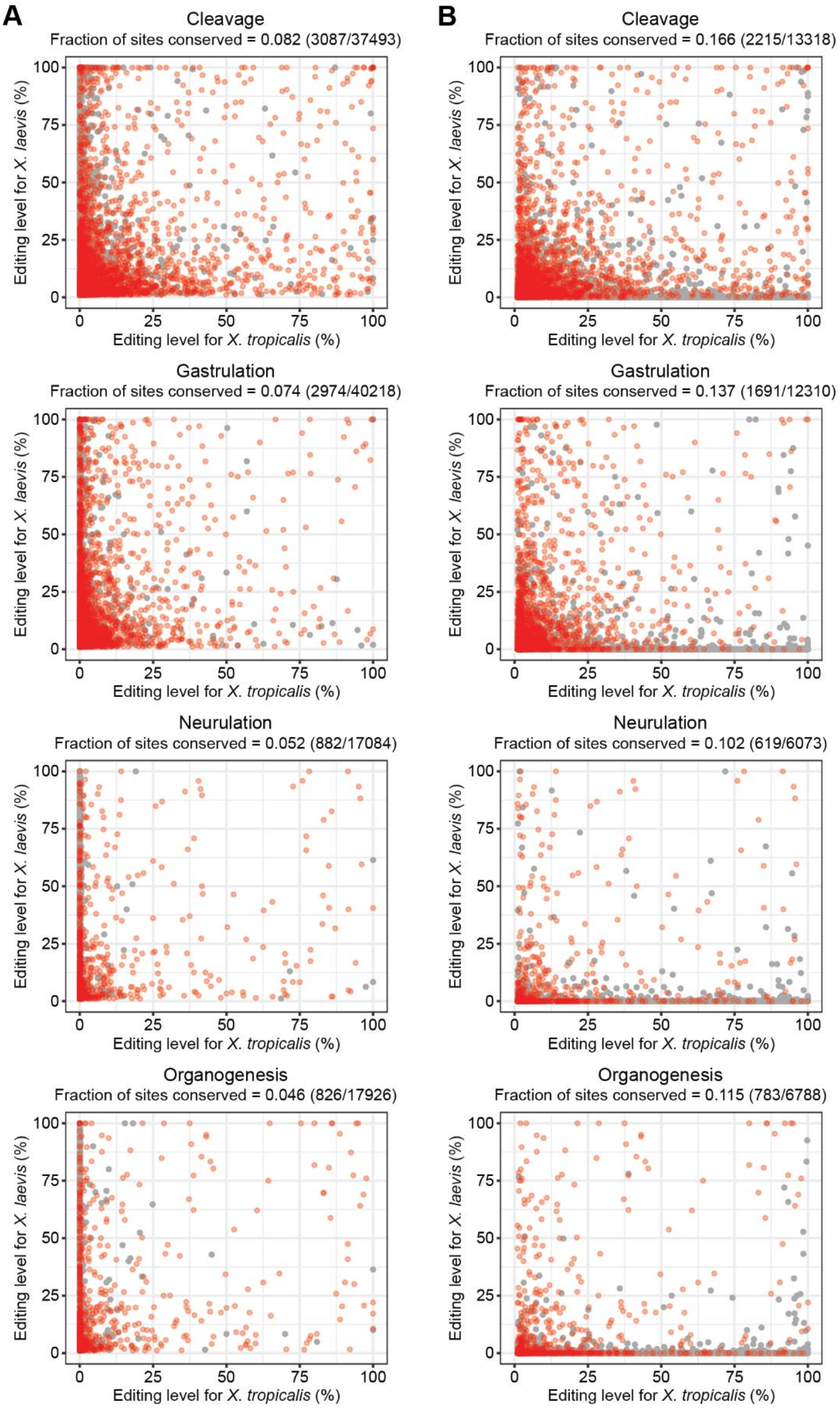

Supp. Fig. S37 Cross-species comparison of editing rates for all matched sites.

(A) Scatterplots showing the modification rates of curated *X. laevis* editing sites and the corresponding lifted over positions in *X. tropicalis*.

(B) Scatterplots showing the modification rates of curated *X. tropicalis* editing sites and the corresponding lifted over positions in *X. laevis*.

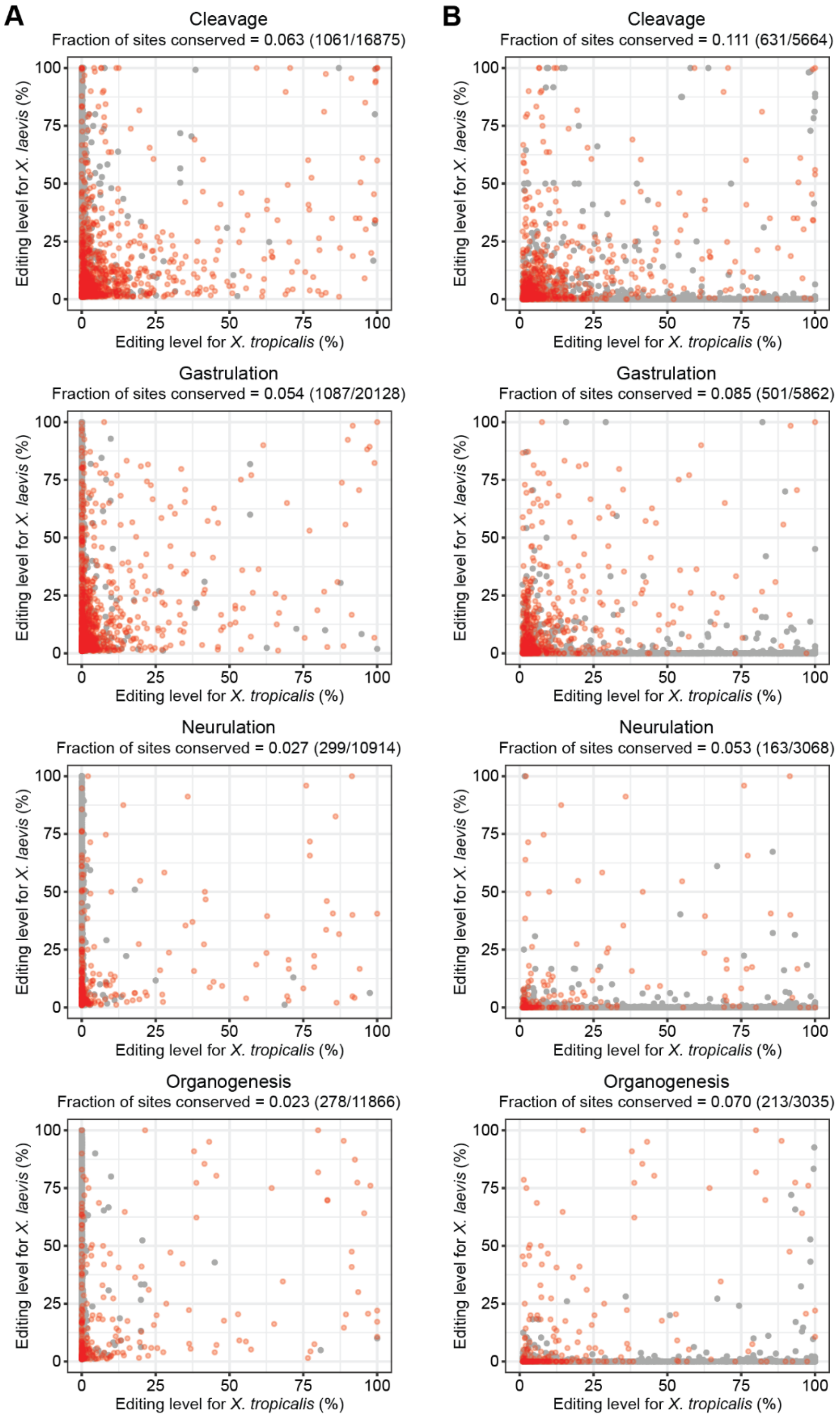

Supp. Fig. S38 Cross-species comparison of editing rates, omitting matched sites in genes with conflicting symbols.

(A) Scatterplots showing the modification rates of curated *X. laevis* editing sites and the corresponding lifted over positions in *X. tropicalis*.

(B) Scatterplots showing the modification rates of curated *X. tropicalis* editing sites and the corresponding lifted over positions in *X. laevis*.

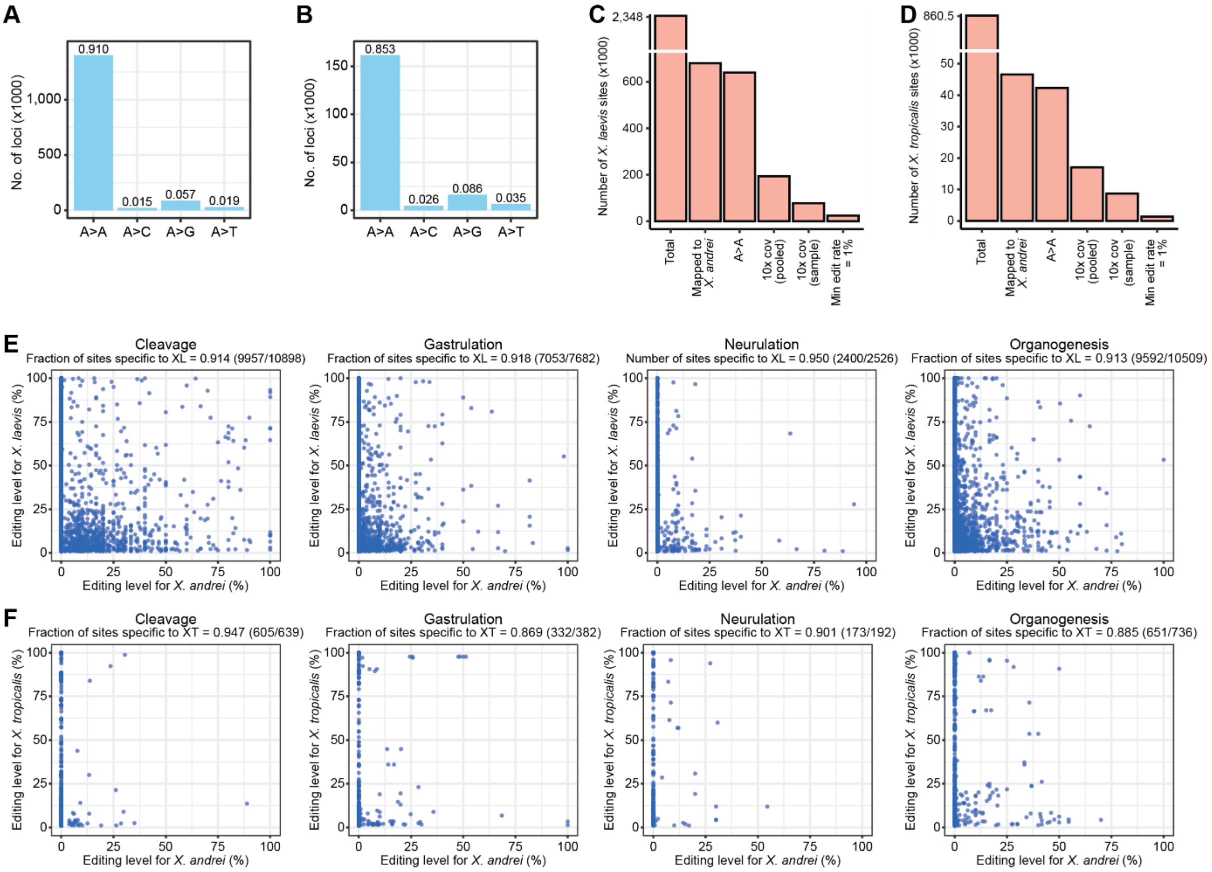

Supp. Fig. S39 Evaluation of *X. laevis* or *X. tropicalis* editing sites in *X. andrei*.

(A) Nucleotide identity of genomic loci in *X. andrei* that had been lifted over from our list of editing sites in *X. laevis*.

(B) Nucleotide identity of genomic loci in *X. andrei* that had been lifted over from our list of editing sites in *X. tropicalis*.

(C) Number of *X. laevis* editing sites that could be analysed in the *X. andrei* transcriptome. Majority of the sites either could not be converted or lacked sufficient sequencing coverage.

(D) Number of *X. tropicalis* editing sites that could be analysed in the *X. andrei* transcriptome. Majority of the sites either could not be converted or lacked sufficient sequencing coverage.

(E) Scatterplots showing the modification rates of curated *X. laevis* editing sites and the corresponding lifted over positions in *X. andrei*. All successfully matched sites are shown.

(F) Scatterplots showing the modification rates of curated *X. tropicalis* editing sites and the corresponding lifted over positions in *X. andrei*. All successfully matched sites are shown.

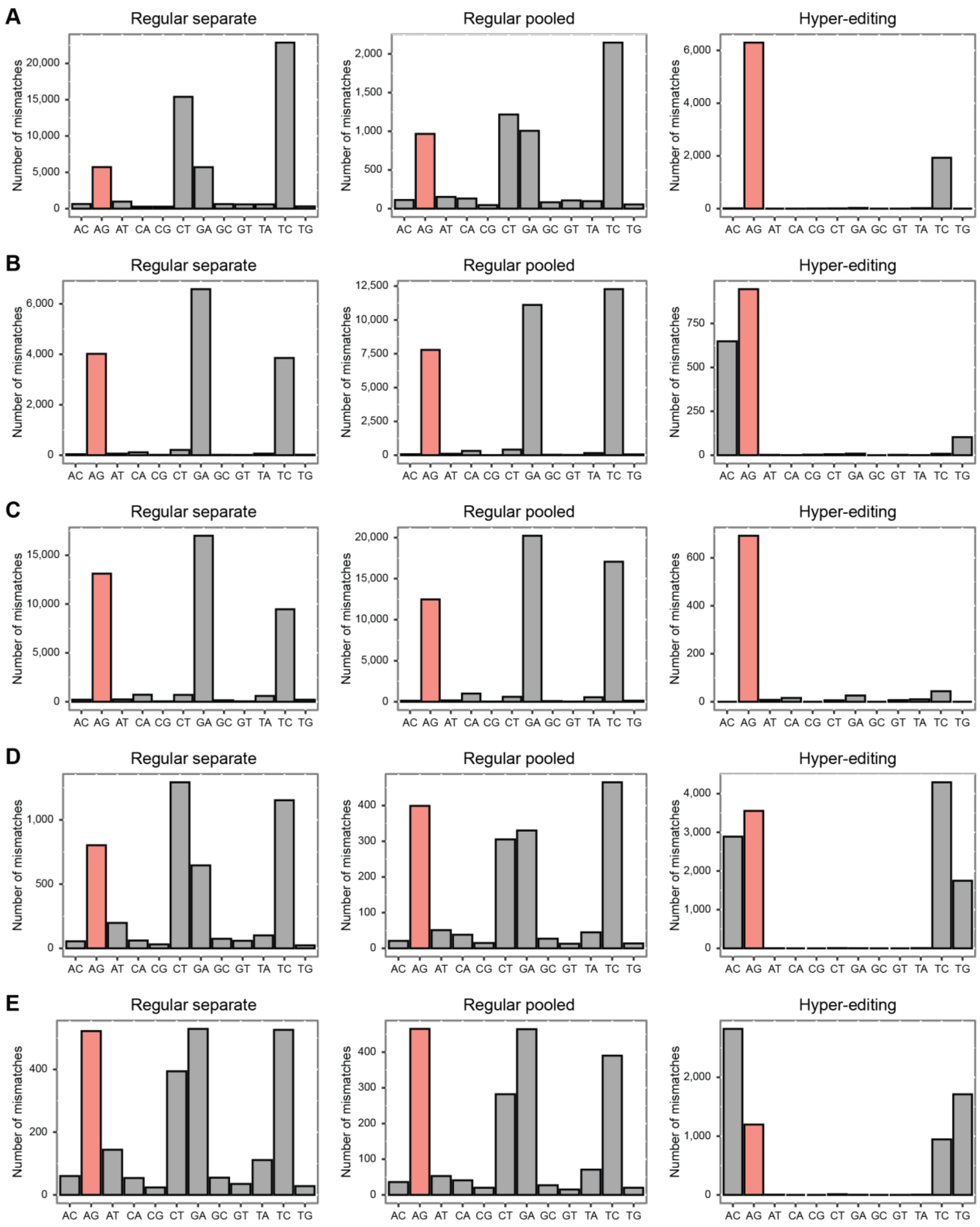

Supp. Fig. S40 Distribution of mismatch types for coding regions in *X. laevis*.

The various graphs show the types of mismatches obtained in the coding regions after the final step of the separate samples analysis workflow, the pooled samples analysis workflow, and the hyper-editing analysis workflow for **(A)** this study (MHT), **(B)** the MK study (poly-A enriched libraries), **(C)** the MK study (Ribo-Zero libraries), **(D)** the DR study (embryonic development), and **(E)** the DR study (adult organs).

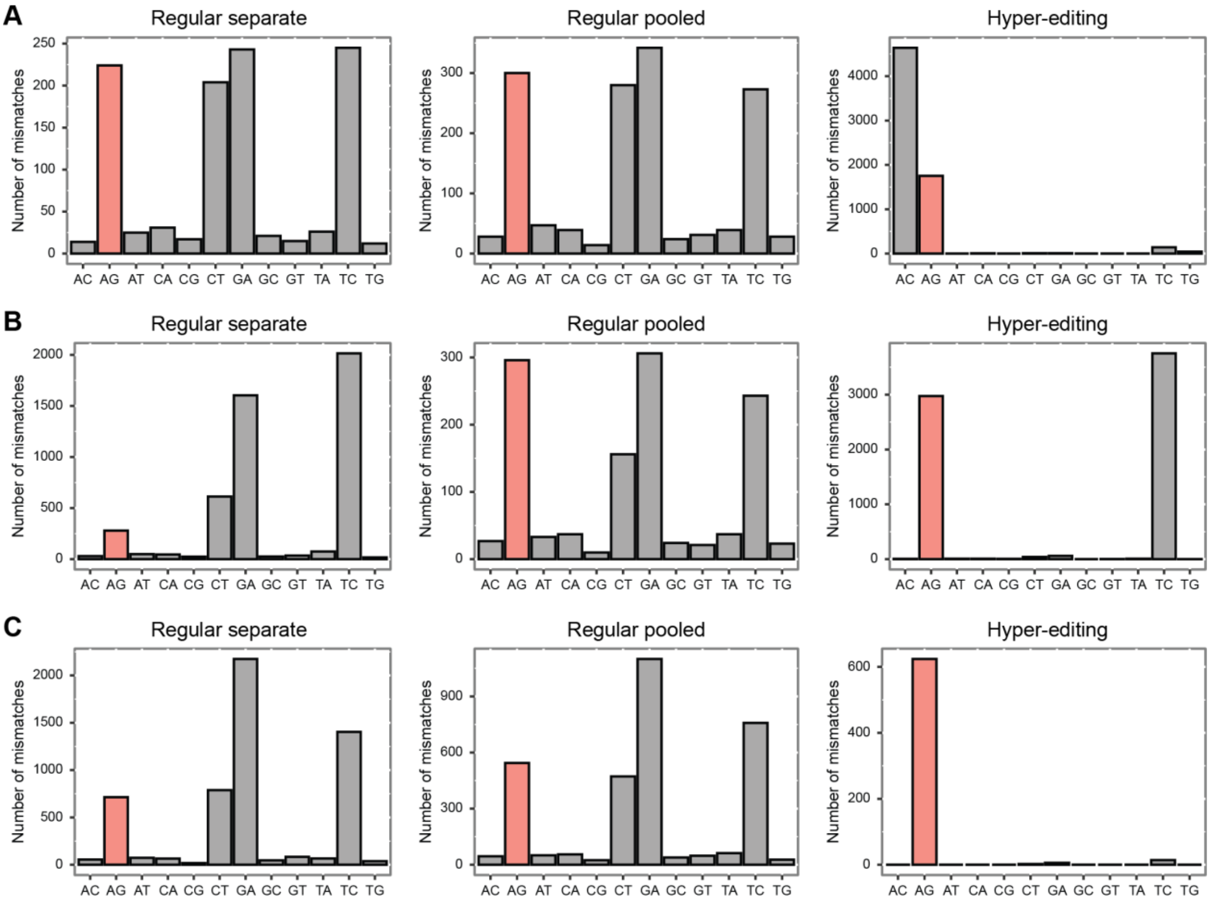

Supp. Fig. S41 Distribution of mismatch types for coding regions in *X. tropicalis*.

The various graphs show the types of mismatches obtained in the coding regions after the final step of the separate samples analysis workflow, the pooled samples analysis workflow, and the hyper-editing analysis workflow for the **(A)** JBL, **(B)** MKK, and **(C)** KW studies.

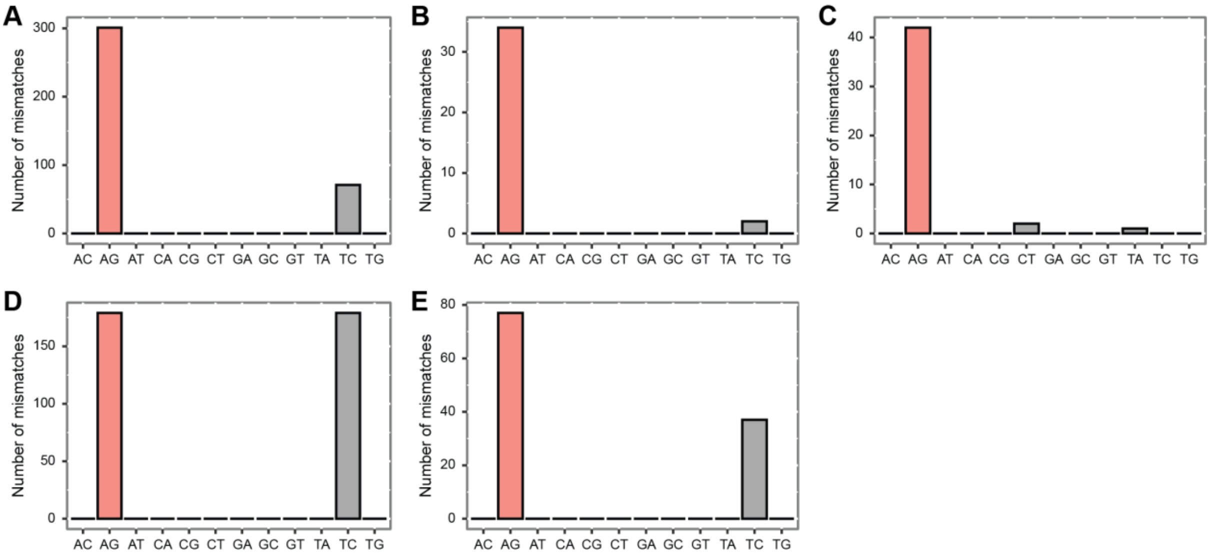

Supp. Fig. S42 Types of coding mismatches identified by both regular read alignment and REDItools and the hyper-editing pipeline in *X. laevis*.

The graphs show the distributions of mismatch types for **(A)** this study (MHT), **(B)** the MK study (poly-A enriched libraries), **(C)** the MK study (Ribo-Zero libraries), **(D)** the DR study (embryonic development), and **(E)** the DR study (adult organs).

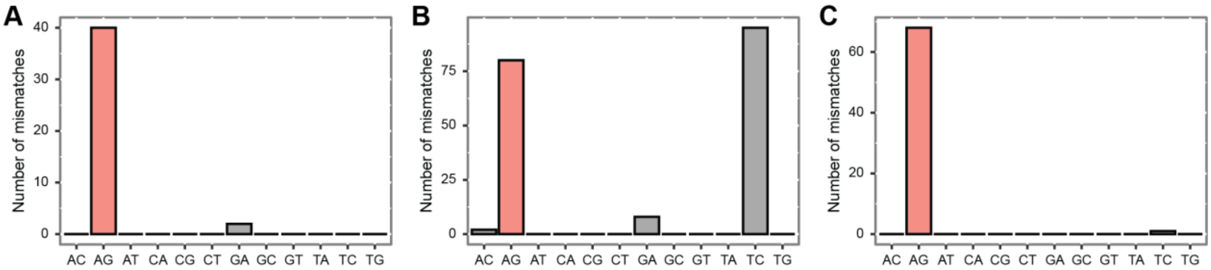

Supp. Fig. S43 Types of coding mismatches identified by both regular read alignment and REDItools and the hyper-editing pipeline in *X. tropicalis*.

The graphs show the distributions of mismatch types for the **(A)** JBL, **(B)** MKK, and **(C)** KW studies.

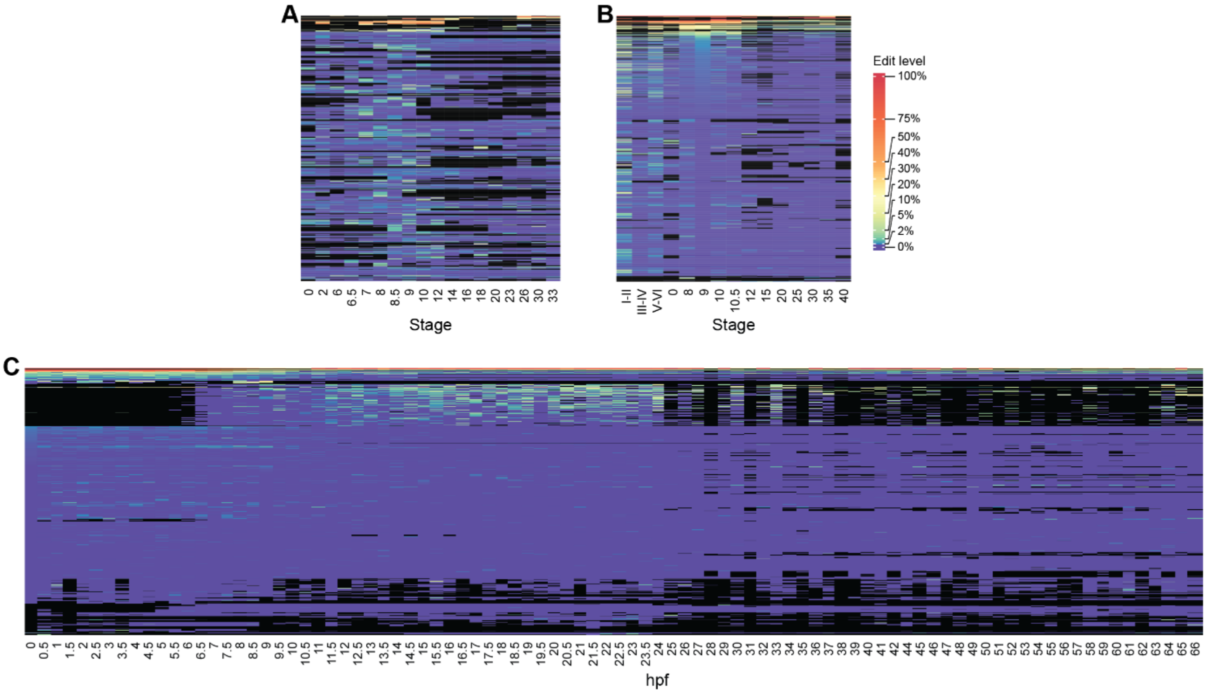

Supp. Fig. S44 Developmental editing profiles of coding sites in *Xenopus*.

Heatmaps showing the editing rates of all the high-confidence coding sites over development for the **(A)** MK (*X. laevis*), **(B)** DR (*X. laevis*), and **(C)** MKK (*X. tropicalis*) studies.

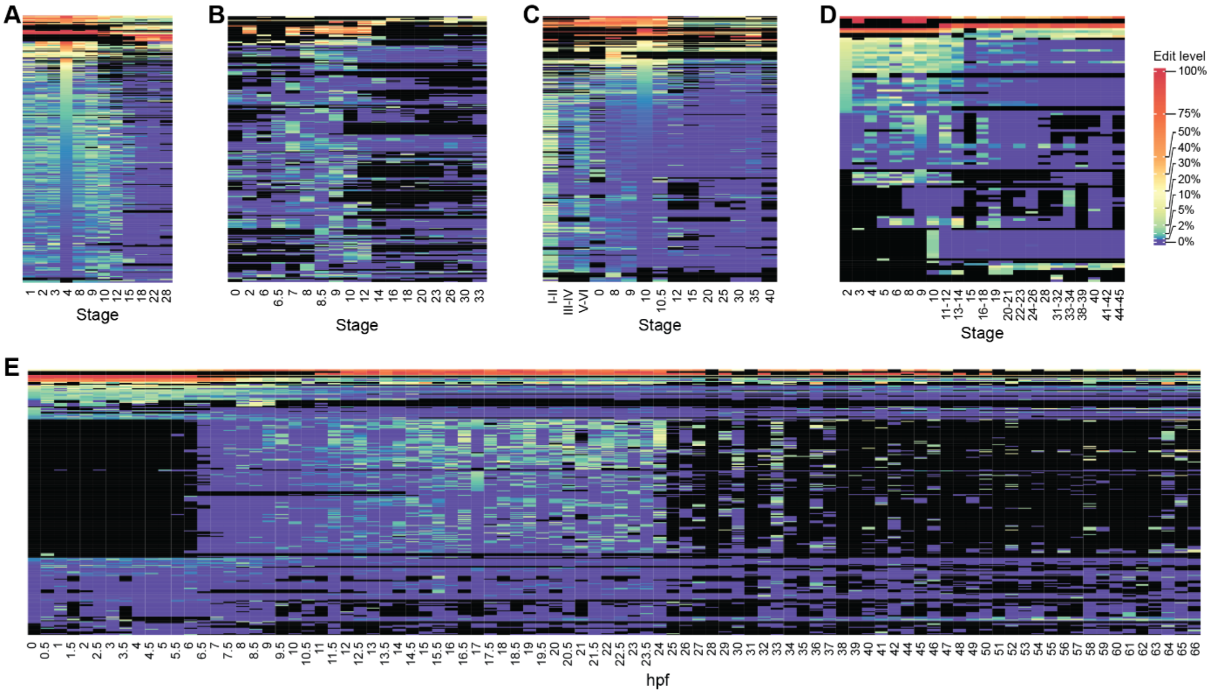

Supp. Fig. S45 Profiles of high-confidence coding sites edited at 5% or higher in *Xenopus*. Heatmaps showing the developmental editing profiles of the subset of coding sites targeted at a minimal rate of 5% for **(A)** this study (*X. laevis*), **(B)** the MK study (*X. laevis*), **(C)** the DR study (*X. laevis*), **(D)** the JBL study (*X. tropicalis*), and **(E)** the MKK study (*X. tropicalis*).

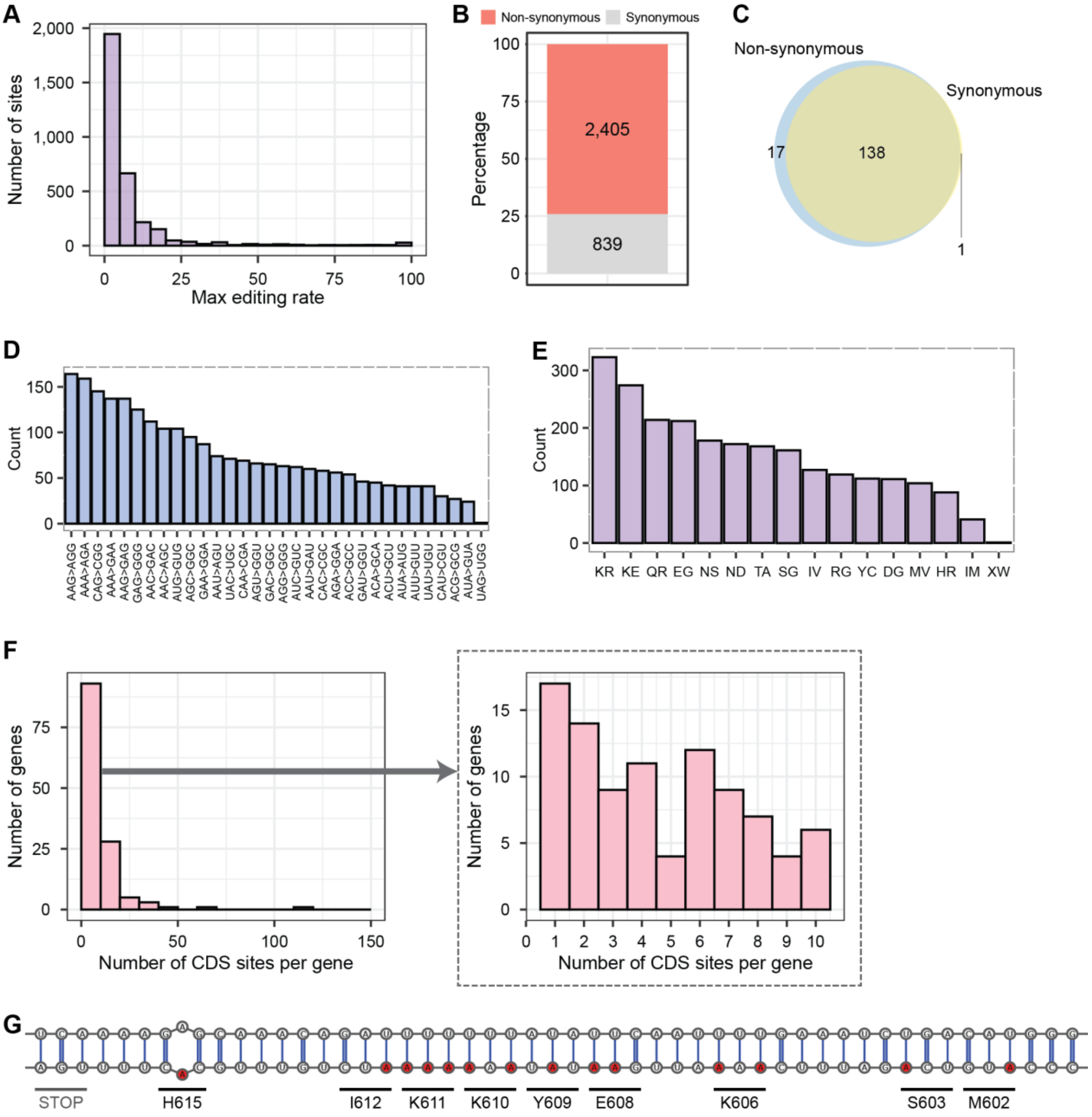

A
Number of sites
2,000
1,500
1,000
500
0
0
25
50
75
100
Max editing rate
B
Non-synonymous
Synonymous
Percentage
100
75
50
25
0
2,405
839
C
Non-synonymous
Synonymous
17
138
1
D
Count
150
100
50
0
AAG>AGG
AAA>AGA
CAG>CGG
AAA>GAA
AAG>GAG
GAG>GGG
AAC>GAC
AAC>AGC
AUG>GUG
AGC>GGC
GAA>GGA
AAU>AGU
UAC>UGC
CAA>CGA
AGU>GGU
GAC>GGC
AGG>GGG
AUC>GUC
AAU>GAU
CAC>CGC
AGA>GGA
ACC>GCC
GAU>GGU
ACU>GCU
ACA>GCA
AUA>AUG
AUU>GUU
UAU>UGU
CAU>CGU
ACG>GCG
AUA>GUA
UAG>UGG
E
Count
300
200
100
0
KR KE QR EG NS ND TA SG IV RG YC DG MV HR IM XW
F
Number of genes
75
50
25
0
0
50
100
150
Number of CDS sites per gene
Number of genes
15
10
5
0
0 1 2 3 4 5 6 7 8 9 10
Number of CDS sites per gene
G
STOP
H615
I612
K611
K610
Y609
E608
K606
S603
M602

Supp. Fig. S46 High-confidence coding sites in *X. laevis*.

(A) Histogram showing the distribution of deamination rates of the coding sites in *X. laevis*.

(B) Extent of recoding by RNA editing in *X. laevis*. Non-synonymous A-to-I editing is more prevalent than synonymous A-to-I editing in the frog.

(C) Venn diagram showing the number of genes containing non-synonymous or synonymous editing events in *X. laevis*.

(D) Histogram showing the distribution of codon changes due to A-to-I editing.

(E) Histogram showing the distribution of amino acid substitutions due to A-to-I editing.

(F) Histograms showing the distribution of coding site counts per gene in *X. laevis*. The left histogram has a bin size of 10, while the right histogram is a zoomed-in graph of the first bin.

(G) Example of a gene with multiple coding sites. The coding region of tmem62, which encodes a putative transmembrane protein, contains a cluster of 14 editing sites (highlighted in red). Based on the xenLae2 genome, the coordinates of these ADAR targets are chr8S: 5280508, 5280503, 5280496, 5280494, 5280489, 5280488, 5280486, 5280484, 5280482, 5280481, 5280480, 5280479, 5280478, and 5280467. Importantly, the editing sites are located within a predicted long dsRNA stem.

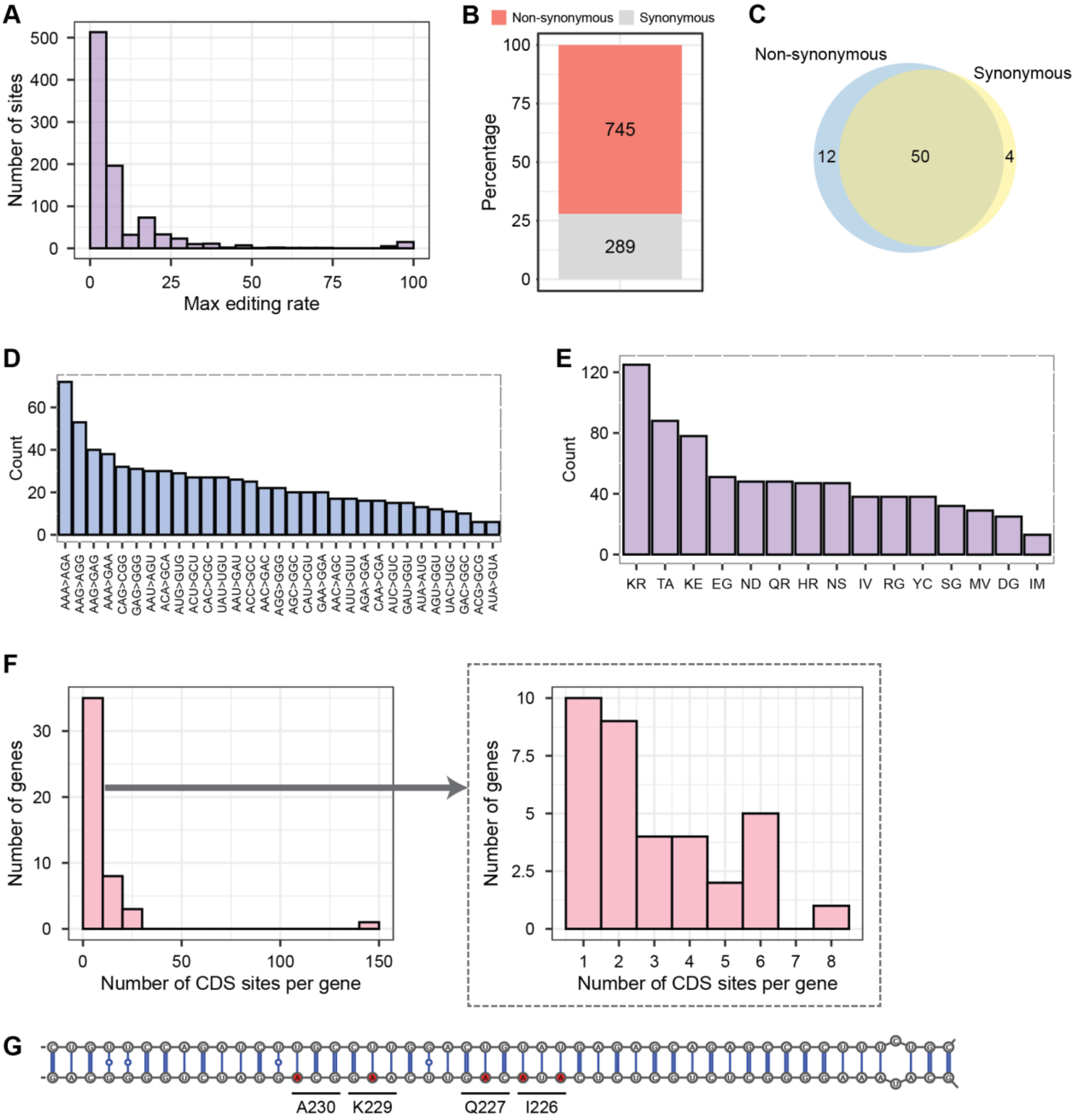
A
Number of sites
500
400
300
200
100
0
0
25
50
75
100
Max editing rate
B
Non-synonymous
Synonymous
Percentage
100
75
50
25
0
745
289
C
Non-synonymous
Synonymous
12
50
4
D
Count
60
40
20
0
AAA>AGA
AAG>AGG
AAG>GAG
AAA>GAA
CAG>CGG
GAG>GGG
AAU>AGU
ACA>GCA
AUG>GUG
ACU>GCU
CAC>CGC
UAU>UGU
AAU>GAU
ACC>GCC
AAC>GAC
AGG>GGG
AGC>GGC
CAU>CGU
GAA>GGA
AAC>AGC
AUU>GUU
AGA>GGA
CAA>CGA
AUC>GUC
GAU>GGU
AUA>AUG
AGU>GGU
UAC>UGC
GAC>GGC
ACG>GCG
AUA>GUA
E
Count
120
80
40
0
KR
TA
KE
EG
ND
QR
HR
NS
IV
RG
YC
SG
MV
DG
IM
F
Number of genes
30
20
10
0
0
50
100
150
Number of CDS sites per gene
Number of genes
10
7.5
5
2.5
0
1
2
3
4
5
6
7
8
Number of CDS sites per gene
G
A230
K229
Q227
I226

Supp. Fig. S47 High-confidence coding sites in *X. tropicalis*.

(A) Histogram showing the distribution of deamination rates of the coding sites in *X. tropicalis*.

(B) Extent of recoding by RNA editing in *X. tropicalis*. Non-synonymous A-to-I editing is more prevalent than synonymous A-to-I editing in the frog.

(C) Venn diagram showing the number of genes containing non-synonymous or synonymous editing events in *X. tropicalis*.

(D) Histogram showing the distribution of codon changes due to A-to-I editing.

(E) Histogram showing the distribution of amino acid substitutions due to A-to-I editing.

(F) Histograms showing the distribution of coding site counts per gene in *X. tropicalis*. The left histogram has a bin size of 10, while the right histogram is a zoomed-in graph of the first bin.

(G) Example of a gene with multiple coding sites. The coding region of crkl gene, which encodes a signal transduction factor, contains a cluster of five major editing sites with maximum deamination rates greater than 10% (highlighted in red). Based on the xenTro9 genome, the coordinates of these ADAR targets are chr1:144359885, 144359887, 144359889, 144359895, and 144359899. Importantly, the editing sites are located within a predicted long dsRNA stem.

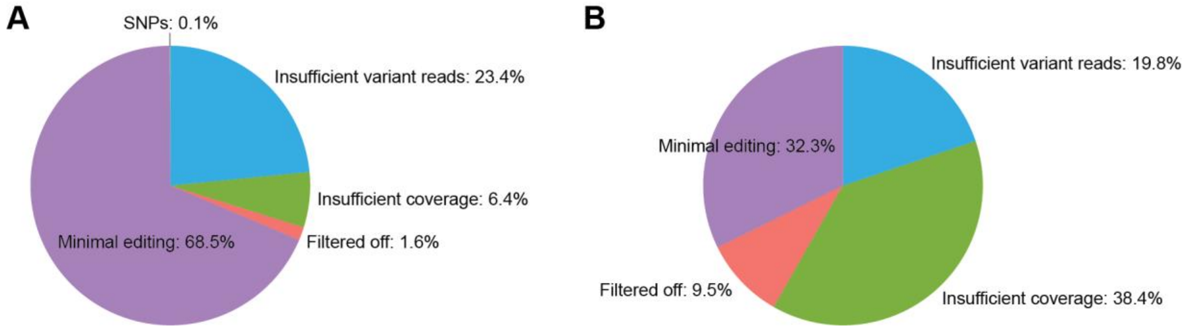

Supp. Fig. S48 Reasons for sites found in one species not detected in the other species. **(A)** Pie chart summarizing why *X. laevis* editing sites could not be detected in *X. tropicalis*. **(B)** Pie chart summarizing why *X. tropicalis* editing sites could not be detected in *X. laevis*.

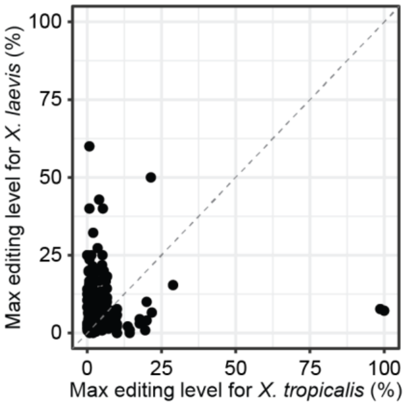

Supp. Fig. S49 Scatterplot showing the editing levels of high-confidence conserved coding sites in *Xenopus*.

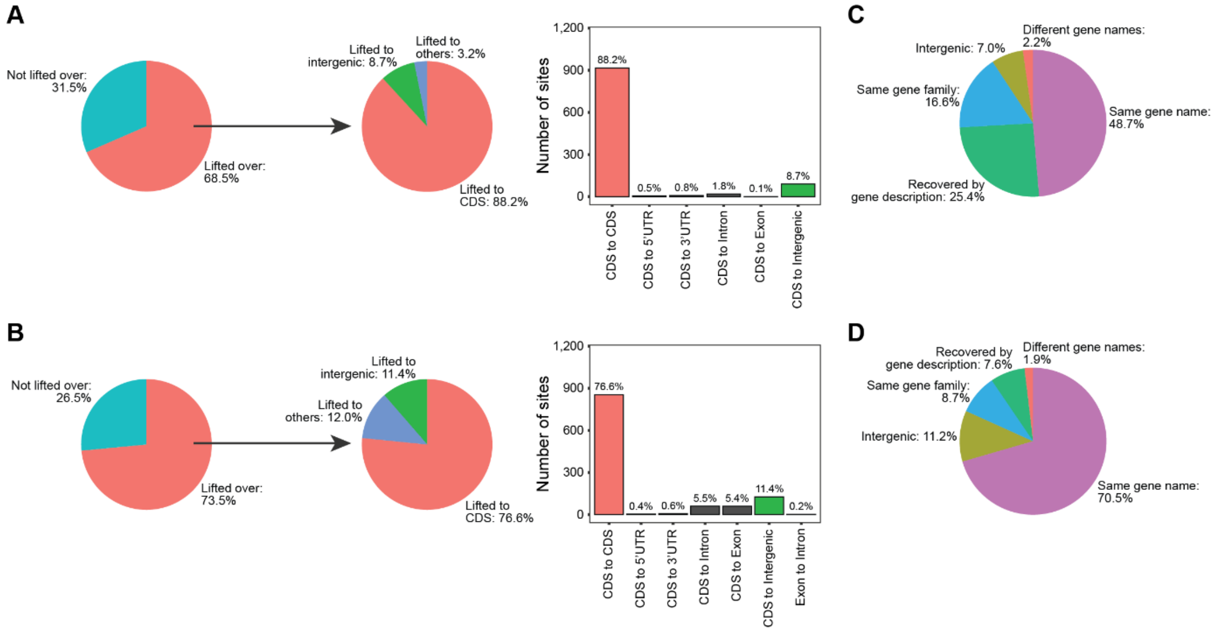

Supp. Fig. S50 Examination of human CDS sites in *Xenopus*.

(A) Most of the 1,517 human CDS sites could be lifted over to *X. laevis* coding regions. A sizeable percentage also mapped to intergenic regions, which may contain unannotated exons.

(B) Most of the 1,517 human CDS sites could be lifted over to *X. tropicalis* coding regions. A sizeable percentage also mapped to intergenic regions, which may contain unannotated exons.

(C) Comparison of successfully converted *X. laevis* loci with the original human genes.

(D) Comparison of successfully converted *X. tropicalis* loci with the original human genes.

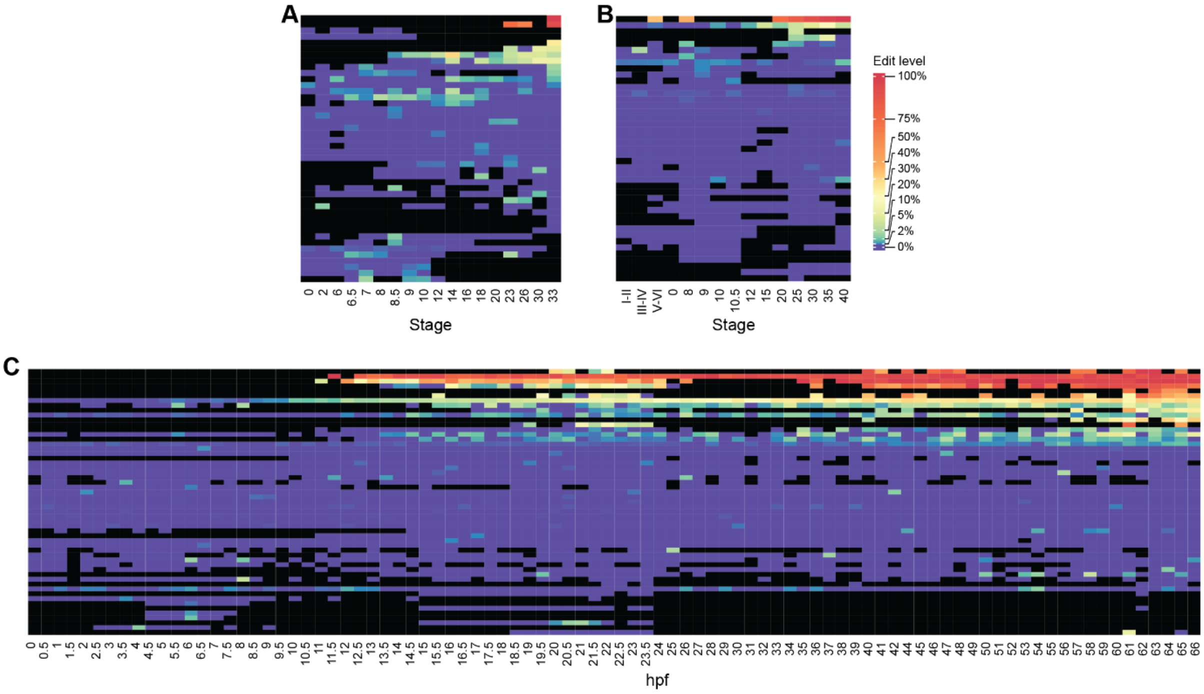

Supp. Fig. S51 Developmental editing profiles of conserved coding sites in *Xenopus*. Heatmaps showing the editing rates of vertebrate conserved coding sites over development for the **(A)** MK (*X. laevis*), **(B)** DR (*X. laevis*), and **(C)** MKK (*X. tropicalis*) studies.

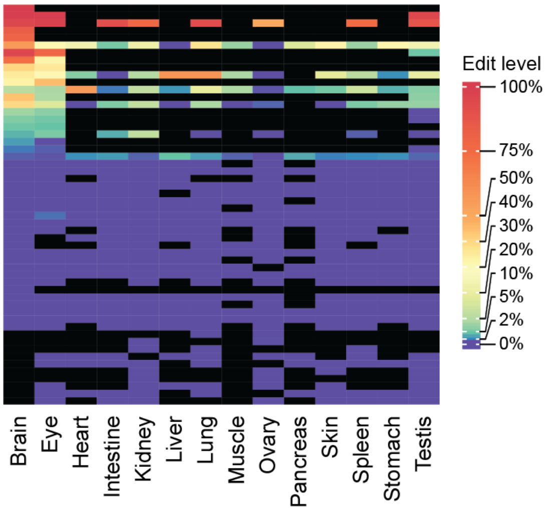

Supp. Fig. S52 Editing profile of conserved coding sites in adult tissues of *X. laevis*.
